# Intestinal microbiota and tuberculosis: Insights from Mendelian randomization

**DOI:** 10.1097/MD.0000000000038762

**Published:** 2024-07-05

**Authors:** Peijun Liu, Yaomei Luo, Minghua Zhang

**Affiliations:** a Department of Respiratory and Critical Care Medicine, The Central Hospital of Enshi Tujia and Miao Autonomous Prefecture, Enshi, China.

**Keywords:** genome-wide association study, gut microbiota, Mendelian randomization, respiratory tuberculosis

## Abstract

Respiratory tuberculosis (RTB), a global health concern affecting millions of people, has been observationally linked to the gut microbiota, but the depth and nature of this association remain elusive. Despite these findings, the underlying causal relationship is still uncertain. Consequently, we used the Mendelian randomization (MR) method to further investigate this potential causal connection. We sourced data on the gut microbiota from a comprehensive genome-wide association study (GWAS) conducted by the MiBioGen Consortium (7686 cases, and 115,893 controls). For RTB, we procured 2 distinct datasets, labeled the Fingen R9 TBC RESP and Fingen R9 AB1 RESP, from the Finnish Genetic Consortium. To decipher the potential relationship between the gut microbiota and RTB, we employed MR on both datasets. Our primary mode of analysis was the inverse variance weighting (IVW) method. To ensure robustness and mitigate potential confounders, we meticulously evaluated the heterogeneity and potential pleiotropy of the outcomes. In the TBC RESP (RTB1) dataset related to the gut microbiota, the IVW methodology revealed 7 microbial taxa that were significantly associated with RTB. In a parallel vein, the AB1 RESP (RTB2) dataset highlighted 4 microbial taxa with notable links. Notably, Lachnospiraceae UCG010 was consistently identified across both datasets. This correlation was especially evident in the data segments designated Fingen R9 TBC RESP (OR = 1.799, 95% CI = 1.243−2.604) and Finngen R9 AB1 RESP (OR = 2.131, 95% CI = 1.088−4.172). Our study identified a causal relationship between particular gut microbiota and RTB at the level of prediction based on genetics. This discovery sheds new light on the mechanisms of RTB development, which are mediated by the gut microbiota.

## 1. Introduction

*Mycobacterium tuberculosis* is a persistent infectious disease known as tuberculosis. Two years ago, there were an estimated 10.6 million new cases. It is believed that nearly 25% of the global population has been exposed to this bacterium at some point in their life.^[1]^ Therefore, new methods for treating anti-tuberculosis and developing novel drugs are urgently needed. As symbiotic bacteria in the human body, the gut microbiota is crucial for maintaining a balance between health and the opposite. It aids in food digestion and breakdown to provide energy and nutrients to the body. Additionally, the microbiota and its metabolic byproducts can directly or indirectly regulate innate and adaptive immunity, influencing the immune system functionality.^[2]^ Moreover, respiratory tuberculosis (RTB) is the most common and contagious form of tuberculosis. The prolonged and extensive use of antibiotics in tuberculosis treatment of tuberculosis undoubtedly impacts the microbial community within the body.

RTB has long been associated with compromised immune function and a reduced ability to clear pathogens from the lungs. Recently, reports have suggested that the gut microbiota contributes to the resistance of the host to tuberculosis, including the reactivation of endogenous infection, the severity of active tuberculosis, and the occurrence of drug-resistant tuberculosis.^[3]^ RTB is likely the result of an immune response imbalance against *M tuberculosis*, as emerging research suggests a significant interaction between the metabolic byproducts of the gut microbiota and the immune system.^[4,5]^ In vitro experiments revealed that lactobacilli isolated from wild boar feces exhibited significant inhibitory effects on *Mycobacterium bovis*.^[6]^ When mice with gut microbiota dysbiosis were fed lactobacilli derived from plants, there was a substantial reduction in inflammatory factors (IFN-γ, IL-6, IL-12, and IL-17) and concurrent inhibition of *M tuberculosis* growth.^[7]^ Continuous intraperitoneal injection of sodium butyrate in mice before *M bovis* infection for 1 month was found to reduce the abundance of bacteria in the lungs and decrease the recruitment of inflammatory cells, thus demonstrating a significant preventive effect against bovine tuberculosis.^[8]^ Research has demonstrated that gut microbiota dysbiosis may result in a variety of inflammatory responses, hampering the capacity of the gut-lung axis to eradicate pathogens from the airways.^[9]^

Mendelian randomization (MR) is a robust analytical approach in observational research that leverages established functional genetic variants to elucidate causal associations between modifiable risk factors and disease outcomes.^[10]^ Nevertheless, MR studies have yet to be conducted to investigate the potential causal connection between the gut microbiota and RTB. In our research, we used MR to examine this potential link, identifying pathogenic bacterial taxa related to the disease.

## 2. Materials and methods

### 2.1. Study design

Throughout the research process, including planning, implementation, and documentation, we strictly followed the guidelines set by the Strengthening the Reporting of Observational Studies in Epidemiology for our epidemiological observations.^[11,12]^ The data for our MR analysis came from genome-wide association studies (GWAS) that were previously published. Before this study clinical tests, the subject provided informed consent, and the pertinent institutional and ethical review boards authorized the research protocol. Consequently, no additional ethical approval was necessary for our investigation.

**Figure 1.**
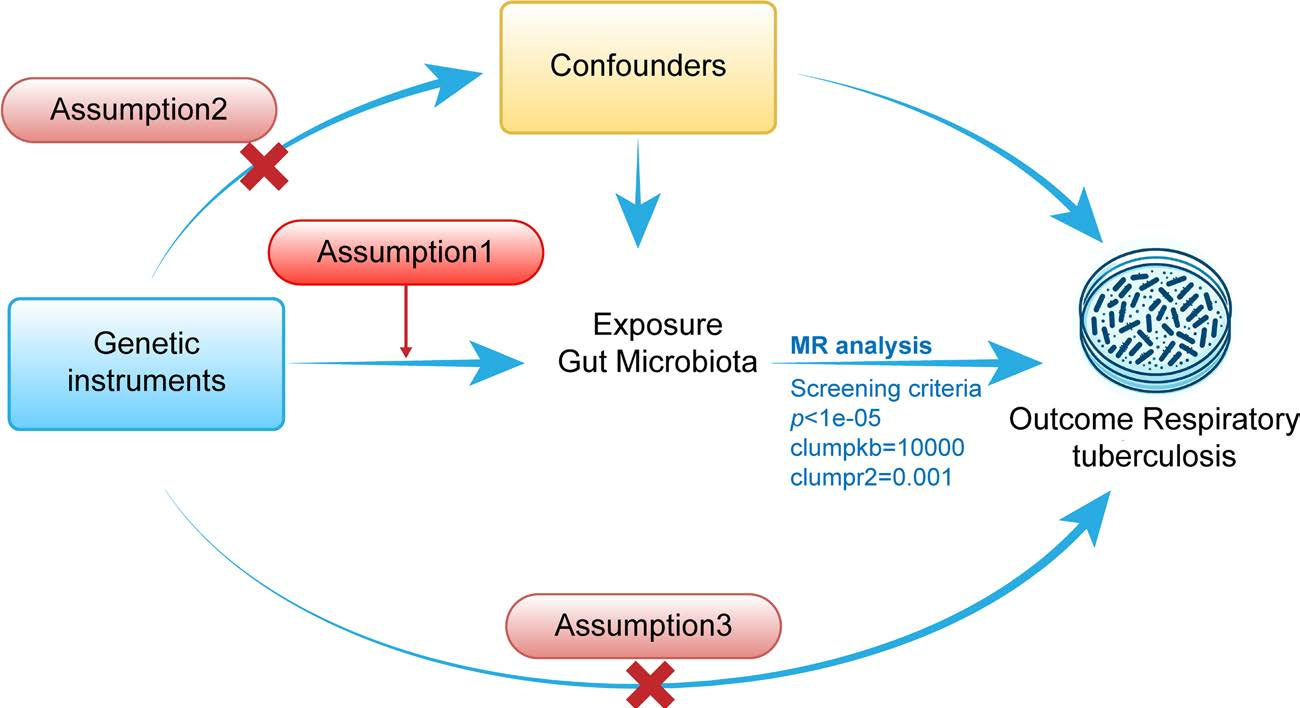
A schematic illustration of the Mendelian randomization analysis process examining the relationship between gut microbiota and respiratory tuberculosis.

As depicted in Figure 1, we chose suitable single nucleotide polymorphisms (SNPs) as instrumental variables (IVs) to examine the link between the gut microbiota and RTB. The MR method was utilized for this purpose. It is predicated on 3 primary presumptions: the chosen IVs have a substantial link with the concerned exposure; possible confounding factors do not affect IVs; and the effect of IVs on the result is mediated only by their linkage with the exposure, with the exception of other pathways.^[13]^

### 2.2. Sources of data

The primary dataset for microbiome information was obtained from the MiBioGen Consortium (www.mibiogen.org). This consortium includes data from 24 cohorts, encompassing 18,340 patients. Within this dataset, there are 211 taxonomic units distributed among 20 orders, 9 classes, 35 families, 131 genera, and 16 species, accompanied by 122,110 SNPs.^[14]^ The 2 RTB datasets were acquired from the consortium FinnGen, a large-scale biobank initiative in Finland that genetically profiles 500,000 cases. The 2 datasets for RTB were obtained through FinnGen Consortium access. FinnGen is a large biobank project conducted in Finland to achieve genetic profiling of 500,000 individuals.^[15]^ The FinnGen R9 TBC RESP dataset consists of 376,715 individuals, all of European descent, sourced from the Finnish National Health Register. This dataset included 1793 patients with RTB and 374,922 control participants. Additionally, the FinnGen R9 AB1 RESP dataset includes 543 instances of RTB and 374,845 controls. The FinnGen R9 TBC RESP and AB1 RESP datasets exhibit distinct characteristics in terms of participant composition, TB case types, and diagnostic criteria. The TBC RESP dataset covers a broader range of TB cases, while AB1 RESP specifically targets RTB cases, categorizing them based on bacteriological and histological confirmation. This contrast underscores the varied focus and methodologies employed in each dataset.

### 2.3. Selection of SNPs

In MR, the exposure was summarized by GWAS information related to the gut microbiota. To ensure adequate selection of IVs, SNPs with *P* values below the genome-wide significance threshold (1 × 10^-5^) were chosen. According to previous research,^[16,17]^ the LD parameter (r^2^) for SNPs was set at 0.001, with a genetic distance of 10000 kb, to examine the independence of variables and LD effects. The PhenoScanner website was subsequently utilized in January 2024 to check for associations between IVs and potential confounders, such as smoking and alcohol consumption.^[18]^ SNPs found to be related to these confounders were removed to avoid horizontal pleiotropy. SNPs with an F-statistic < 10 were not included to rule out mild instrument bias that violated the MR assumption.^[19]^ The F-statistic was calculated using the formula F = R^2^(n − k − 1)/k(1 − R^2^),where R^2^ is the proportion of variance explained by the selected SNPs, n is the sample size, and k is the number of IVs.

### 2.4. MR analyses

An MR analysis of 2 samples was performed using the inverse variance-weighted (IVW) method to investigate the potential causal relationship between the intestinal microbiota and RTB.^[20]^ Additional analysis techniques included MR–Egger, simple mode, weighted mode, and weighted median methods.^[21]^ Approximate odds ratios (OR) and 95% confidence intervals (CI) are provided, with exposure aggregated through the standard deviation. The nominal level of significance was established at *P* < .05. The gut microbiota showing the most significant association with RTB was identified by taking the intersection of the 2 RTB datasets in the final MR analysis.

### 2.5. Analysis of pleiotropy and heterogeneity

To assess heterogeneity, the IVW and MR-Egger methodologies were utilized, and the outcomes were quantified using Cochran Q statistic, which was employed to investigate the heterogeneity of the SNPs.^[22]^ A statistically significant result of this test would indicate considerable variation.^[23]^ Testing for MR–Egger intercepts were also conducted to examine the possibility of pleiotropy.^[24]^ The “leave-one-out” method was used to assess the causal genetic consequences of any outlier SNPs and to assess whether the MR estimates would be influenced by removing those outliers. If at least one of these outliers was determined to be the cause of the MR estimation changes, the outliers were disregarded, and the MR was repeated.

## 3. Results

### 3.1. Selection of IVs

Based on the criteria for selecting IVs, we screened a large-scale gut microbiota GWAS dataset. We extracted 1531 SNPs associated with the outcome (RTB) at a significance level of *P* < 1 × 10^−5^, with a genetic distance of kb = 10000 and an LD parameter (r^2^) of 0.001. All F-statistics for IVs were greater than 10, signifying that the chosen SNPs presented substantial IV effects and minimal instrument bias. The detailed characteristics of all SNPs were presented in Supplementary Table S1, http://links.lww.com/MD/N71.

### 3.2. MR analysis results

**Figure 2.**
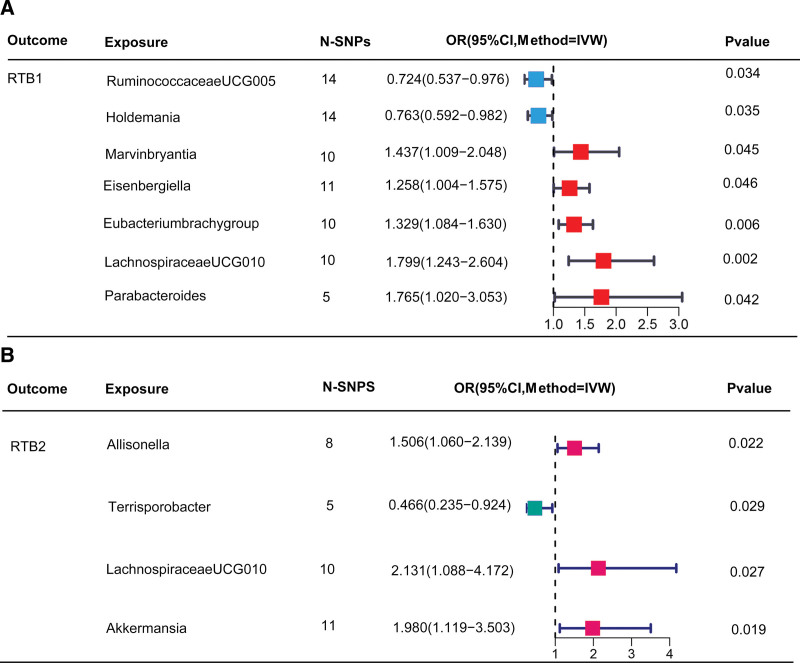
Associations between gut microbiota and RTB. (A) Positive correlations between specific microbial taxa and RTB1. (B) Association between gut microbiota and RTB2 analyzed using the IVW method. IVW = inverse variance-weighted, OR = odds ratios, RTB = respiratory tuberculosis, RTB1 = FinnGen R9 TBC RESP (RTB data), RTB2 = FinnGen R9 AB1 RESP (RTB data).

According to the gut microbiota and TBC RESP (RTB1) data, the IVW method revealed that 7 microbial taxa have a nominally significant association with RTB. Specifically, Ruminococcaceae UCG005 (OR = 0.724, 95% CI = 0.537−0.976, *P* = .034) and Holdemania (OR = 0.763, 95% CI = 0.592−0.982, *P* = .035) may be associated with a reduced risk of RTB onset. In contrast, Marvinbryantia (OR = 1.437, 95% CI: 1.009−2.048, *P* = .045), Eisenbergiella (OR = 1.258, 95% CI: 1.004−1.575, *P* = .046), the Eubacterium brachy group (OR = 1.329, 95% CI: 1.084−1.630, *P* = .006), LachnospiraceaeUCG010 (OR = 1.799, 95% CI: 1.243−2.604, *P* = .002), and Parabacteroides (OR = 1.765, 95% CI: 1.020−3.053, *P* = .042) demonstrated a positive correlation with RTB1 (Fig. 2A).

We further analyzed the relationship between the gut microbiota and the AB1 RESP(RTB2). The IVW method revealed that 4 microbial taxa had a nominally significant association with RTB. Specifically, Terrisporobacter (OR = 0.466, 95% CI = 0.235–0.924, *P* = .029) may be associated with a reduced risk of RTB onset. In contrast, Allisonella (OR = 1.506, 95% CI: 1.060–2.139, *P* = .022), LachnospiraceaeUCG010 (OR = 2.131, 95% CI = 1.088–4.172, *P* = .027), and Akkermansia (OR = 1.980, 95% CI = 1.119–3.503, *P* = .019) demonstrated a positive correlation with RTB (Fig. 2B). A comparison of the TBC RESP (RTB1) and AB1 RESP (RTB2) datasets indicated the existence of the “Lachnospiraceae UCG010” taxon in the gut microbiota.

### 3.3. Lachnospiraceae UCG010 is potentially associated with the advancement of RTB

**Figure 3.**
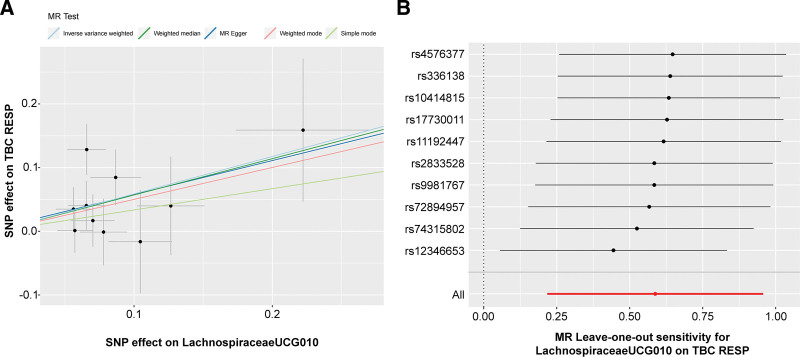
MR results visualizing the association of Lachnospiraceae UCG010 with RTB1. (A) Scatter plot representing the MR results of the relationship between Lachnospiraceae UCG010 and RTB1. (B) A graphical representation illustrating the leave-one-out analysis of the relationship between Lachnospiraceae UCG010 and RTB1. MR = Mendelian randomization.

**Figure 4.**
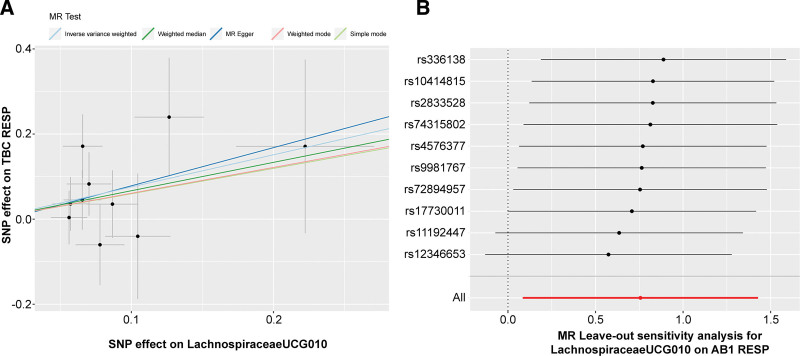
MR results visualizing the association of Lachnospiraceae UCG010 with RTB2. (A) Scatter plot representing the MR results of the relationship between Lachnospiraceae UCG010 and RTB2. (B) A visual depiction showcasing the leave-one-out analysis delineating the association between Lachnospiraceae UCG010 and RTB2. MR = Mendelian randomization.

The relationships between Lachnospiraceae UCG010 and RTB1 and RTB2 were initially examined using MR-Egger and IVW tests. The heterogeneity of the outcomes was consistently assessed, and all *P* values exceeded 0.05, indicating an absence of heterogeneity (Table S2, http://links.lww.com/MD/N72). The MR-Egger intercept test and the MR-PROSSE Global test were employed to assess horizontal pleiotropy among the IVs and the outcomes (RTB1, RTB2), revealing no evidence of pleiotropy (Table S3, http://links.lww.com/MD/N73). Scatter plots were generated to visualize the MR data for the association of Lachnospiraceae UCG010 with RTB1 (Fig. 3A) and RTB2 (Fig. 4A). A noticeable upward slope indicates that as the abundance of Lachnospiraceae UCG010 increases, the RTB incidence gradually increases. The leave-one-out examination revealed that no SNPs with substantial variation that could bias genetic predictions were identified (Figs. 3B and 4B). Funnel plot analysis of the relationships between Lachnospiraceae UCG010 and RTB1 and RTB2 revealed a uniformly scattered distribution of points with no discernible pattern, indicating a lack of positive associations or causal effects (Fig. S1, http://links.lww.com/MD/N69). The forest plot provides a graphical representation of the association between Lachnospiraceae UCG010 and RTB1, as well as between UCG010 and RTB2. Each data point on the plot corresponds to an estimated effect size, while the horizontal lines indicate the confidence intervals. The overall pattern observed in the plot suggested a positive causal relationship between Lachnospiraceae UCG010 and both RTB1 and RTB2 (Fig. S2, http://links.lww.com/MD/N70).

## 4. Discussion

We employed extensive GWAS summary statistics along with MR to explore the potential causal association between gut microbiota and RTB. Various elements of the gastrointestinal microbiota could either elevate the risk of RTB or have the opposite effects. Due to the limited available GWAS data on RTB, we selected 2 other datasets from the FinnGen Consortium for analysis. The link between Lachnospiraceae UCG010 and RTB1 and RTB2 risk suggests a greater risk of RTB. The close and substantial interaction between the host and gut microbiota emphasizes the significance of sustaining a dynamic equilibrium in this symbiosis.^[25]^ Tuberculous infection can lead to systemic multiorgan involvement, with respiratory and intestinal tuberculosis being the most common manifestations.^[26]^ The primary pathological and physiological features of RTB include *M tuberculosis* infection, lung lesions, immune response, and inflammatory reaction, which may be associated with changes in the gut microbiota composition and behavior.^[27]^

LachnospiraceaeUCG010, a member of the Lachnospiraceae family, is involved in the production of butyrate, a short-chain fatty acid.^[28]^ The principal source of energy for gut cells is butyrate, which can enhance the health and integrity of these cells by providing energy and helping to maintain the integrity of the gut barrier.^[29]^ However, when dysbiosis occurs in gut microbiota, especially during certain inflammatory conditions, such as tuberculosis infection, an overgrowth of Lachnospiraceae disrupts the balance among gut bacteria and may increase the likelihood of inflammation and gastrointestinal discomfort.^[30]^ Excessive growth of Lachnospiraceae and Lactobacillaceae leads to a decrease in the pH of the colon, disrupting normal fermentation in the intestines and potentially causing colonic spasms.^[31]^ Patients with tuberculosis infection are typically treated with antibiotics such as rifampicin, isoniazid, and fluoroquinolones.^[32]^ However, a study by Haibo Mu et al demonstrated that ciprofloxacin hydrochloride induces an overgrowth of the Lachnospiraceae family, suggesting that antibiotic treatment can also disrupt the gut microbiota balance.^[33]^ The primary immunological response to *M tuberculosis* infection is cell-mediated and known as delayed-type hypersensitivity, or Type IV. Helper T cells, specifically Th1 cells, play a pivotal role in this response. These cells generate cytokines such as IFN-γ and TNF-α in response to infection, thus activating macrophages to eliminate *M tuberculosis*.^[34]^ Shang et al reported a prevalence of 45.5% for allergic diseases among children, encompassing both respiratory allergies, such as allergic rhinitis and asthma, and skin allergies, including atopic dermatitis and eczema. The prevalence of tuberculosis caused by Lachnospiraceae was significantly greater in neonates with allergies than in infants without allergies.^[35]^ Our MR study likewise confirmed an increased risk of RTB associated with Lachnospiraceae UCG010.

Malnutrition caused by TB increases the likelihood of TB infection. Inadequate dietary intake results in deficiencies in protein, energy, and micronutrients, which compromise gut immunological functions.^[36]^ Additionally, anti-tuberculosis medications have various effects on the gastrointestinal tract, including nausea, vomiting, and diarrhea, and can further disrupt the gut microbiota in tuberculosis patients. The integrity of the gut barrier depends on the balance between pathogenic and good bacteria.^[37]^ Probiotic supplementation may aid in maintaining gut barrier function and preventing inflammation due to the translocation of pathogenic bacteria, mitigating RTB risk. Our study revealed that increasing the abundance of Ruminococcaceae UCG005, Holdemania, and Terrisporobacter may be beneficial for treating RTB. The Ruminococcaceae family is a group of anaerobic bacteria found in the intestines of humans and other mammals. It is crucial for the fermentation of cellulose and other complex carbohydrates, thereby assisting the host in obtaining energy.^[35]^

Our research has several remarkable advantages. This is the premier study to use MR to explore the association between gut microbiota and RTB, effectively reducing the impact of confounding factors and identifying potential bacterial candidates such as Lachnospiraceae UCG for further functional research. From a diagnostic perspective, there should be an increased focus on screening for RTB in patients with gut microbiota dysbiosis. Moreover, from a therapeutic standpoint, modulating the gut microbiota is promising as an adjunctive approach for RTB treatment.

Despite these limitations, our study still has certain limitations. First, most individuals included in the gut microbiota GWAS analysis were of European ancestry, which could contribute to population stratification, and the findings may only partially extend to populations outside of Europe, such as those of East Asian origin. Second, although the MR method utilizes genetic variants for causal inference in epidemiology, it does not provide direct mechanistic evidence for causal relationships. The third limitation of our study is the initial failure to apply multiple correction adjustments, such as Bonferroni or FDR, to our statistical analyses.^[38]^ Determining the particular pathways, mechanisms, and target genes involved in the relationship between the gut microbiota and RTB thus requires further investigation. Considering these limitations, a cautious interpretation of these findings is warranted.

## 5. Conclusion

Overall, our results provide rudimentary proof of a probable causal link between the gut microbiota and RTB. Among the identified microbial strains, Lachnospiraceae UCG010 is a promising candidate and may serve as a novel biomarker for treating and preventing RTB. However, additional further research is needed to elucidate the exact relationship between the gut microbiota and RTB and to explore the foundational molecular mechanisms involved.

## Author contributions

**Conceptualization:** Peijun Liu.

**Data curation:** Yaomei Luo.

**Investigation:** Yaomei Luo.

**Methodology:** Minghua Zhang.

**Project administration:** Minghua Zhang.

**Software:** Peijun Liu.

**Writing – original draft:** Peijun Liu, Yaomei Luo.

**Writing – review & editing:** Minghua Zhang.

## Supplementary Material

**Figure:**
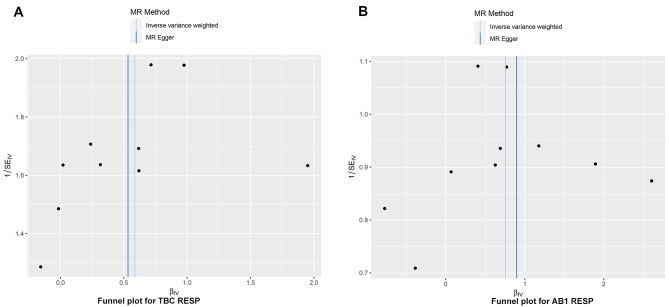


**Figure:**
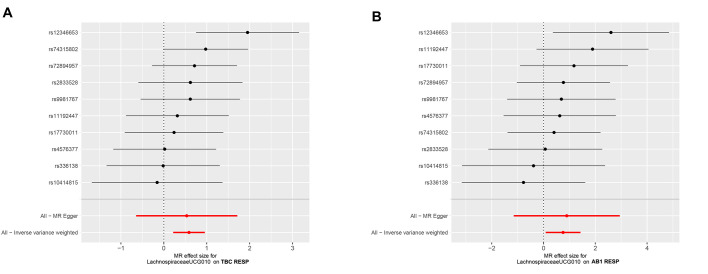


## References

[1] Bagcchi S. WHO’s global tuberculosis report 2022. Lancet Microbe. 2023;4:e20. 36521512 10.1016/S2666-5247(22)00359-7

[2] Rooks MG Garrett WS. Gut microbiota, metabolites and host immunity. Nat Rev Immunol. 2016;16:341–52. 27231050 10.1038/nri.2016.42 PMC5541232

[3] Wood MR Yu EA Mehta S. The human microbiome in the fight against tuberculosis. Am J Trop Med Hyg. 2017;96:1274–84. 28719264 10.4269/ajtmh.16-0581 PMC5462560

[4] Gonçalves P Araújo JR Di Santo JP. A cross-talk between microbiota-derived short-chain fatty acids and the host mucosal immune system regulates intestinal homeostasis and inflammatory Bowel disease. Inflamm Bowel Dis. 2018;24:558–72. 29462379 10.1093/ibd/izx029

[5] Yao Y Cai X Fei W. The role of short-chain fatty acids in immunity, inflammation and metabolism. Crit Rev Food Sci Nutr. 2022;62:1–12. 33261516 10.1080/10408398.2020.1854675

[6] Bravo M Combes T Martinez FO. Lactobacilli isolated from wild boar (Sus scrofa) antagonize Mycobacterium bovis Bacille Calmette-Guerin (BCG) in a species-dependent manner. Front Microbiol. 2019;10:1663. 31417502 10.3389/fmicb.2019.01663 PMC6683848

[7] Negi S Pahari S Bashir H. Gut microbiota regulates mincle mediated activation of lung dendritic cells to protect against *Mycobacterium tuberculosis*. Front Immunol. 2019;10:1142. 31231363 10.3389/fimmu.2019.01142 PMC6558411

[8] Zhang K Hussain T Wang J. Sodium butyrate abrogates the growth and pathogenesis of *Mycobacterium bovis* via regulation of cathelicidin (LL37) expression and NF-κB signaling. Front Microbiol. 2020;11:433. 32265874 10.3389/fmicb.2020.00433 PMC7096352

[9] Budden KF Gellatly SL Wood DL. Emerging pathogenic links between microbiota and the gut-lung axis. Nat Rev Microbiol. 2017;15:55–63. 27694885 10.1038/nrmicro.2016.142

[10] Sanderson E Glymour MM Holmes MV. Mendelian randomization. Nat Rev Methods Primers. 2022;2:6. 37325194 10.1038/s43586-021-00092-5 PMC7614635

[11] Lachat C Hawwash D Ocké MC. Strengthening the reporting of observational studies in epidemiology-nutritional epidemiology (STROBE-nut): an extension of the STROBE statement. PLoS Med. 2016;13:e1002036. 27270749 10.1371/journal.pmed.1002036 PMC4896435

[12] Skrivankova VW Richmond RC Woolf BAR. Strengthening the reporting of observational studies in epidemiology using Mendelian randomization: the STROBE-MR statement. JAMA. 2021;326:1614–21. 34698778 10.1001/jama.2021.18236

[13] Richmond RC Davey Smith G. Mendelian randomization: concepts and scope. Cold Spring Harb Perspect Med. 2022;12:a040501. 34426474 10.1101/cshperspect.a040501 PMC8725623

[14] Kurilshikov A Medina-Gomez C Bacigalupe R. Large-scale association analyses identify host factors influencing human gut microbiome composition. Nat Genet. 2021;53:156–65. 33462485 10.1038/s41588-020-00763-1 PMC8515199

[15] Kurki MI Karjalainen J Palta P. FinnGen provides genetic insights from a well-phenotyped isolated population. Nature. 2023;613:508–18. 36653562 10.1038/s41586-022-05473-8 PMC9849126

[16] Wang D Chen X Li Z. Association of the gut microbiota with coronary artery disease and myocardial infarction: a Mendelian randomization study. Front Genet. 2023;14:1158293. 37113988 10.3389/fgene.2023.1158293 PMC10126394

[17] Ma J Li J Jin C. Association of gut microbiome and primary liver cancer: a two-sample Mendelian randomization and case-control study. Liver Int. 2023;43:221–33. 36300678 10.1111/liv.15466

[18] Staley JR Blackshaw J Kamat MA. PhenoScanner: a database of human genotype-phenotype associations. Bioinformatics. 2016;32:3207–9. 27318201 10.1093/bioinformatics/btw373 PMC5048068

[19] Sanderson E Davey Smith G Windmeijer F. An examination of multivariable Mendelian randomization in the single-sample and two-sample summary data settings. Int J Epidemiol. 2019;48:713–27. 30535378 10.1093/ije/dyy262 PMC6734942

[20] Zheng J Baird D Borges MC. Recent developments in Mendelian randomization studies. Curr Epidemiol Rep. 2017;4:330–45. 29226067 10.1007/s40471-017-0128-6 PMC5711966

[21] Zhang K Zhou J Li A. Mendelian randomization study reveals the effect of idiopathic pulmonary fibrosis on the risk of erectile dysfunction. Front Med. 2023;10:1162153. 10.3389/fmed.2023.1162153 PMC10370277 37502356

[22] Shi Q Wang Q Wang Z. Systemic inflammatory regulators and proliferative diabetic retinopathy: a bidirectional Mendelian randomization study. Front Immunol. 2023;14:1088778. 36845092 10.3389/fimmu.2023.1088778 PMC9950638

[23] Higgins JP Thompson SG. Quantifying heterogeneity in a meta-analysis. Stat Med. 2002;21:1539–58. 12111919 10.1002/sim.1186

[24] Bowden J Del Greco MF Minelli C. A framework for the investigation of pleiotropy in two-sample summary data Mendelian randomization. Stat Med. 2017;36:1783–802. 28114746 10.1002/sim.7221 PMC5434863

[25] Thursby E Juge N. Introduction to the human gut microbiota. Biochem J. 2017;474:1823–36. 28512250 10.1042/BCJ20160510 PMC5433529

[26] Al-Zanbagi AB Shariff MK. Gastrointestinal tuberculosis: a systematic review of epidemiology, presentation, diagnosis and treatment. Saudi J Gastroenterol. 2021;27:261–74. 34213424 10.4103/sjg.sjg_148_21 PMC8555774

[27] Osei Sekyere J Maningi NE Fourie PB. *Mycobacterium tuberculosis*, antimicrobials, immunity, and lung-gut microbiota crosstalk: current updates and emerging advances. Ann N Y Acad Sci. 2020;1467:21–47. 31989644 10.1111/nyas.14300

[28] Meehan CJ Beiko RG. A phylogenomic view of ecological specialization in the Lachnospiraceae, a family of digestive tract-associated bacteria. Genome Biol Evol. 2014;6:703–13. 24625961 10.1093/gbe/evu050 PMC3971600

[29] Molino S Lerma-Aguilera A Jiménez-Hernández N. Evaluation of the effects of a short supplementation with tannins on the gut microbiota of healthy subjects. Front Microbiol. 2022;13:848611. 35572677 10.3389/fmicb.2022.848611 PMC9093706

[30] Wang F Li Q He Q. Temporal variations of the ileal microbiota in intestinal ischemia and reperfusion. Shock. 2013;39:96–103. 23247126 10.1097/SHK.0b013e318279265f

[31] Park T Cheong H Yoon J. Comparison of the fecal microbiota of horses with intestinal disease and their healthy counterparts. Vet Sci. 2021;8:113. 34204317 10.3390/vetsci8060113 PMC8234941

[32] Dartois VA Rubin EJ. Anti-tuberculosis treatment strategies and drug development: challenges and priorities. Nat Rev Microbiol. 2022;20:685–701. 35478222 10.1038/s41579-022-00731-y PMC9045034

[33] Mu H Bai H Sun F. Pathogen-targeting glycovesicles as a therapy for salmonellosis. Nat Commun. 2019;10:4039. 31492864 10.1038/s41467-019-12066-z PMC6731243

[34] Fogel N. Tuberculosis: a disease without boundaries. Tuberculosis (Edinb). 2015;95:527–31. 26198113 10.1016/j.tube.2015.05.017

[35] Shang Q Shan X Cai C. Dietary fucoidan modulates the gut microbiota in mice by increasing the abundance of Lactobacillus and Ruminococcaceae. Food Funct. 2016;7:3224–32. 27334000 10.1039/c6fo00309e

[36] Kant S Gupta H Ahluwalia S. Significance of nutrition in pulmonary tuberculosis. Crit Rev Food Sci Nutr. 2015;55:955–63. 24915351 10.1080/10408398.2012.679500

[37] Gensollen T Iyer SS Kasper DL. How colonization by microbiota in early life shapes the immune system. Science. 2016;352:539–44. 27126036 10.1126/science.aad9378 PMC5050524

[38] Xiao QA Qin L Yu J. The causality between gut microbiome and chronic regional pain: a Mendelian randomization analysis. Front Microbiol. 2024;15:1329521. 38486697 10.3389/fmicb.2024.1329521 PMC10938595

