# Supplementary material for: Intestinal microbiota and tuberculosis: Insights from Mendelian randomization
Source: Medicine (Baltimore). 2024 Jul 5;103(27):e38762. doi: 10.1097/MD.0000000000038762 (PMC11250452; doi:10.1097/MD.0000000000038762)
Supplement: Supplementary file 1 [file medi-103-e38762-s001.docx]

**TableS1** The instrumental variables (IVs) were extracted from the MiBioGen dataset.

| chr.  exposure | pos.  exposure | SNP | other_allele.  exposure | effect_allele.exposure | beta.  exposure | se.  exposure | pval.  exposure | samplesize.  exposure | exposure | eaf.  exposure | R^2^ | F |
| --- | --- | --- | --- | --- | --- | --- | --- | --- | --- | --- | --- | --- |
| 6 | 67207371 | rs34583783 | T | G | 0.126595837 | 0.026846063 | 4.49E-06 | 7468 | Actinomyces | 0.1024 | 0.002968808 | 22.23111771 |
| 15 | 99492313 | rs2715439 | T | C | 0.074668399 | 0.016482184 | 6.27E-06 | 7468 | Actinomyces | 0.4543 | 0.002740615 | 20.51765995 |
| 10 | 4782841 | rs4146653 | A | G | 0.098522403 | 0.021418242 | 4.50E-06 | 7468 | Actinomyces | 0.1968 | 0.002825331 | 21.15368445 |
| 6 | 133007480 | rs35011108 | G | A | 0.232633598 | 0.051204357 | 6.34E-06 | 3515 | Actinomyces | 0.0636 | 0.005837984 | 20.62927004 |
| 6 | 169224781 | rs4073240 | A | G | 0.074968682 | 0.016736817 | 7.94E-06 | 7468 | Actinomyces | 0.3827 | 0.002679443 | 20.0584647 |
| 10 | 127532121 | rs7915461 | C | T | 0.187759792 | 0.040163611 | 5.92E-06 | 5282 | Actinomyces | 0.0577 | 0.004120483 | 21.84616522 |
| 3 | 101352439 | rs71315246 | G | A | -0.09698092 | 0.021925041 | 9.83E-06 | 7468 | Actinomyces | 0.1431 | 0.002613071 | 19.56029702 |
| 10 | 121356829 | rs10787984 | C | G | 0.094315957 | 0.021351309 | 9.62E-06 | 7468 | Actinomyces | 0.1759 | 0.002606056 | 19.50765281 |
| 4 | 171281359 | rs7680684 | T | C | -0.083381015 | 0.016890088 | 9.77E-07 | 8243 | Adlercreutzia | 0.325 | 0.002947835 | 24.36492903 |
| 1 | 201061818 | rs2147798 | G | C | 0.092295902 | 0.019212124 | 1.40E-06 | 8243 | Adlercreutzia | 0.1958 | 0.002791994 | 23.07324668 |
| 5 | 94059857 | rs80078995 | T | A | -0.113239745 | 0.023258622 | 1.57E-06 | 8243 | Adlercreutzia | 0.1342 | 0.00286746 | 23.69869359 |
| 18 | 75009770 | rs2717140 | T | C | -0.119225209 | 0.025108114 | 2.05E-06 | 8243 | Adlercreutzia | 0.1113 | 0.00272795 | 22.54252779 |
| 6 | 123909083 | rs9490822 | T | C | -0.073451202 | 0.015578896 | 2.54E-06 | 8243 | Adlercreutzia | 0.4473 | 0.002689488 | 22.22383784 |
| 5 | 31991834 | rs12522517 | T | A | -0.104884371 | 0.023469475 | 4.41E-06 | 8187 | Adlercreutzia | 0.1272 | 0.002433503 | 19.96681016 |
| 7 | 48844151 | rs13231526 | A | C | 0.143237051 | 0.031165443 | 4.81E-06 | 8051 | Adlercreutzia | 0.0636 | 0.002616835 | 21.11816816 |
| 1 | 69444163 | rs6664405 | C | T | -0.095308388 | 0.02107517 | 5.23E-06 | 8051 | Adlercreutzia | 0.1441 | 0.002533777 | 20.44617958 |
| 10 | 135204950 | rs1046175 | G | C | 0.112757932 | 0.025599407 | 6.36E-06 | 8051 | Adlercreutzia | 0.1262 | 0.002404027 | 19.3966395 |
| 17 | 48864179 | rs9915817 | C | T | 0.074921889 | 0.016833007 | 8.22E-06 | 8243 | Adlercreutzia | 0.329 | 0.002397542 | 19.8056301 |
| 3 | 105355971 | rs55719207 | A | G | -0.069924275 | 0.015803609 | 9.61E-06 | 8243 | Adlercreutzia | 0.4175 | 0.002369343 | 19.57212592 |
| 11 | 98386085 | rs11604400 | T | C | -0.102524228 | 0.023483368 | 9.74E-06 | 8107 | Adlercreutzia | 0.1153 | 0.00234559 | 19.0557075 |
| 4 | 95027272 | rs11729256 | C | T | 0.075047253 | 0.015018395 | 6.58E-07 | 11860 | Akkermansia | 0.2386 | 0.002100992 | 24.96601847 |
| 11 | 130280667 | rs4936098 | G | A | 0.064922492 | 0.01359338 | 1.10E-06 | 11857 | Akkermansia | 0.34 | 0.001920109 | 22.80668541 |
| 6 | 56341481 | rs9349825 | G | A | -0.070340692 | 0.014713291 | 2.60E-06 | 11857 | Akkermansia | 0.2793 | 0.0019239 | 22.85179629 |
| 4 | 100544188 | rs74542928 | C | T | 0.112622737 | 0.023643438 | 1.48E-06 | 11215 | Akkermansia | 0.0785 | 0.002019082 | 22.68576664 |
| 16 | 81063149 | rs2602429 | T | C | 0.074535241 | 0.015620127 | 2.72E-06 | 11689 | Akkermansia | 0.2217 | 0.001944159 | 22.76564122 |
| 1 | 41074472 | rs61779207 | A | G | -0.076053856 | 0.01677691 | 6.32E-06 | 11590 | Akkermansia | 0.1779 | 0.001769969 | 20.54677019 |
| 1 | 105422565 | rs11184341 | C | G | 0.065585479 | 0.01422364 | 4.06E-06 | 11863 | Akkermansia | 0.2783 | 0.001789047 | 21.25791358 |
| 10 | 5064327 | rs4242783 | A | G | 0.068545381 | 0.014770062 | 3.00E-06 | 11689 | Akkermansia | 0.2873 | 0.001839141 | 21.53363926 |
| 18 | 49473635 | rs117107102 | G | A | 0.204406166 | 0.043162894 | 3.01E-06 | 4974 | Akkermansia | 0.0447 | 0.004488563 | 22.41775985 |
| 15 | 97570657 | rs12908520 | A | G | 0.061772023 | 0.01309539 | 2.26E-06 | 11862 | Akkermansia | 0.4563 | 0.001872298 | 22.24710763 |
| 12 | 130309670 | rs111862613 | C | T | 0.091119928 | 0.019674815 | 3.39E-06 | 11620 | Akkermansia | 0.166 | 0.001842461 | 21.44522679 |
| 20 | 31867840 | rs941682 | A | G | -0.063295745 | 0.01437768 | 9.17E-06 | 11808 | Akkermansia | 0.3111 | 0.001638637 | 19.37750528 |
| 7 | 135778919 | rs67281112 | C | G | 0.063457498 | 0.013784589 | 3.92E-06 | 17090 | Alistipes | 0.2167 | 0.001238506 | 21.18982605 |
| 1 | 19790706 | rs67705352 | G | T | -0.053240398 | 0.011086401 | 1.65E-06 | 17567 | Alistipes | 0.3738 | 0.001311095 | 23.05961254 |
| 7 | 62443498 | rs11769002 | A | G | -0.052879265 | 0.010938851 | 1.45E-06 | 17571 | Alistipes | 0.4006 | 0.00132817 | 23.3656544 |
| 11 | 17511427 | rs7129639 | A | C | -0.052495538 | 0.010958377 | 1.78E-06 | 17571 | Alistipes | 0.4016 | 0.001304335 | 22.94578425 |
| 9 | 14080129 | rs1689282 | C | A | -0.052006142 | 0.011394196 | 5.28E-06 | 17090 | Alistipes | 0.34 | 0.001217505 | 20.83008642 |
| 5 | 177755179 | rs11958296 | G | A | -0.098122204 | 0.021827201 | 9.30E-06 | 16214 | Alistipes | 0.0706 | 0.001244822 | 20.2062124 |
| 21 | 40580258 | rs8130320 | G | A | -0.049001972 | 0.01071701 | 4.84E-06 | 17571 | Alistipes | 0.4821 | 0.001188412 | 20.90404771 |
| 11 | 130887552 | rs2875322 | C | T | -0.058094464 | 0.013143238 | 8.78E-06 | 17459 | Alistipes | 0.2068 | 0.001117789 | 19.53507051 |
| 10 | 127787142 | rs2290844 | T | C | 0.081396908 | 0.019163185 | 9.10E-06 | 17571 | Alistipes | 0.1133 | 0.001025742 | 18.0397705 |
| 8 | 144428720 | rs2450745 | C | A | -0.080523959 | 0.018470178 | 7.12E-06 | 17571 | Alistipes | 0.0825 | 0.001080543 | 19.00459599 |
| 9 | 112120264 | rs62576416 | C | T | 0.049264678 | 0.010973282 | 7.50E-06 | 17564 | Alistipes | 0.3976 | 0.001146243 | 20.15342013 |
| 13 | 38057357 | rs1107244 | A | G | 0.075855776 | 0.017118221 | 3.59E-06 | 17571 | Alistipes | 0.0785 | 0.001116294 | 19.63408208 |
| 17 | 52776479 | rs34417064 | G | A | -0.04820195 | 0.010686843 | 7.01E-06 | 17570 | Alistipes | 0.5119 | 0.001156528 | 20.34140288 |
| 2 | 178000949 | rs12990744 | T | C | -0.077661791 | 0.017298561 | 8.21E-06 | 16694 | Alistipes | 0.1233 | 0.001205898 | 20.15314435 |
| 20 | 41191335 | rs4810359 | G | A | -0.065242793 | 0.014615527 | 7.50E-06 | 17380 | Alistipes | 0.1471 | 0.001145219 | 19.92444187 |
| 9 | 79110160 | rs602075 | G | A | 0.168974277 | 0.029697566 | 3.57E-08 | 3212 | Allisonella | 0.2306 | 0.009978575 | 32.35407491 |
| 9 | 100150556 | rs35778461 | T | C | 0.146678707 | 0.029718557 | 1.21E-06 | 3212 | Allisonella | 0.1918 | 0.007526999 | 24.34491219 |
| 2 | 33607071 | rs6742198 | A | G | 0.149151971 | 0.031648438 | 3.35E-06 | 3182 | Allisonella | 0.1958 | 0.006931583 | 22.19628878 |
| 5 | 114286707 | rs1901739 | G | T | 0.115768513 | 0.024862618 | 3.59E-06 | 3212 | Allisonella | 0.495 | 0.006704864 | 21.66789336 |
| 7 | 137968010 | rs76904847 | A | G | 0.148523492 | 0.033485691 | 6.09E-06 | 3182 | Allisonella | 0.1899 | 0.006144617 | 19.66068842 |
| 12 | 28018998 | rs35110698 | C | T | -0.146321972 | 0.032085207 | 5.72E-06 | 3212 | Allisonella | 0.1491 | 0.006433257 | 20.78446701 |
| 10 | 122413451 | rs7898615 | G | T | 0.167966358 | 0.037358631 | 8.87E-06 | 3212 | Allisonella | 0.1153 | 0.006254067 | 20.20189916 |
| 11 | 88632972 | rs594561 | T | C | 0.112230607 | 0.025168069 | 9.41E-06 | 3212 | Allisonella | 0.4414 | 0.006152717 | 19.87249125 |
| 18 | 75370866 | rs685403 | C | G | -0.175203225 | 0.040447416 | 4.88E-06 | 3212 | Allisonella | 0.1083 | 0.005807611 | 18.75133252 |
| 8 | 69994816 | rs12675596 | T | G | 0.145668934 | 0.029067823 | 9.64E-07 | 3008 | Alloprevotella | 0.2445 | 0.008279806 | 25.09689556 |
| 4 | 189076705 | rs58212166 | G | A | -0.161505876 | 0.035910233 | 7.94E-06 | 3005 | Alloprevotella | 0.1332 | 0.006686243 | 20.21394454 |
| 21 | 40993008 | rs34619204 | A | G | -0.156036945 | 0.034439247 | 8.84E-06 | 3003 | Alloprevotella | 0.1998 | 0.006789434 | 20.51437315 |
| 3 | 79794395 | rs17380632 | T | A | 0.125563764 | 0.028040851 | 9.27E-06 | 3009 | Alloprevotella | 0.3032 | 0.00661972 | 20.03814513 |
| 3 | 152805600 | rs4680035 | G | A | -0.119599445 | 0.025944593 | 4.99E-06 | 3007 | Alloprevotella | 0.5457 | 0.007017345 | 21.23614313 |
| 1 | 234126516 | rs4364940 | G | A | 0.126417748 | 0.028220735 | 8.58E-06 | 3008 | Alloprevotella | 0.2793 | 0.006626954 | 20.05351886 |
| 21 | 36053643 | rs2154444 | G | T | 0.138234323 | 0.030896071 | 8.37E-06 | 3009 | Alloprevotella | 0.2227 | 0.006608812 | 20.00490583 |
| 12 | 117811409 | rs816292 | C | T | -0.113002639 | 0.022040046 | 2.64E-07 | 5017 | Anaerofilum | 0.2425 | 0.005212409 | 26.2771965 |
| 2 | 191139151 | rs79598899 | T | C | 0.182585804 | 0.035728447 | 3.75E-07 | 4709 | Anaerofilum | 0.0825 | 0.005515376 | 26.1048528 |
| 1 | 247145690 | rs4506496 | A | G | 0.103089882 | 0.021286122 | 1.49E-06 | 4935 | Anaerofilum | 0.337 | 0.004730343 | 23.44568659 |
| 3 | 129686434 | rs712981 | C | A | 0.100759617 | 0.020290248 | 6.83E-07 | 5051 | Anaerofilum | 0.4185 | 0.00485854 | 24.65053341 |
| 10 | 125294406 | rs17105491 | C | G | -0.19310744 | 0.04111866 | 1.57E-06 | 4856 | Anaerofilum | 0.0596 | 0.004521404 | 22.04657828 |
| 11 | 1051715 | rs10794359 | C | T | -0.095352431 | 0.020059979 | 2.23E-06 | 4994 | Anaerofilum | 0.4871 | 0.00450395 | 22.58544445 |
| 14 | 30991358 | rs17096874 | T | C | -0.126336895 | 0.02689189 | 2.86E-06 | 5051 | Anaerofilum | 0.1809 | 0.004350577 | 22.06204386 |
| 17 | 6450532 | rs356049 | A | G | 0.132602907 | 0.028980209 | 6.56E-06 | 4994 | Anaerofilum | 0.1859 | 0.004174819 | 20.928067 |
| 2 | 3838940 | rs1563175 | C | A | 0.092383381 | 0.020215708 | 5.54E-06 | 5051 | Anaerofilum | 0.4443 | 0.004117565 | 20.87554366 |
| 2 | 76698142 | rs17012738 | G | T | 0.090341716 | 0.020023943 | 7.24E-06 | 5051 | Anaerofilum | 0.4076 | 0.004013779 | 20.34723895 |
| 9 | 104339812 | rs9299345 | C | T | -0.136399548 | 0.030237163 | 8.04E-06 | 5051 | Anaerofilum | 0.1173 | 0.004012548 | 20.34097525 |
| 12 | 67167461 | rs4244069 | A | G | -0.146774518 | 0.032659424 | 9.81E-06 | 5030 | Anaerofilum | 0.1133 | 0.003999226 | 20.18884935 |
| 16 | 77574020 | rs7193624 | T | C | 0.075064239 | 0.015072346 | 5.35E-07 | 16926 | Anaerostipes | 0.1491 | 0.001463235 | 24.80008305 |
| 10 | 117383184 | rs2804244 | G | A | -0.053088986 | 0.011098265 | 2.04E-06 | 16926 | Anaerostipes | 0.4553 | 0.001350074 | 22.87954502 |
| 21 | 25873624 | rs62215703 | A | G | 0.064496549 | 0.013670195 | 1.98E-06 | 15550 | Anaerostipes | 0.2197 | 0.00142946 | 22.25705305 |
| 2 | 228018671 | rs2396460 | C | T | -0.051265275 | 0.010955542 | 2.91E-06 | 16912 | Anaerostipes | 0.4394 | 0.00129307 | 21.8941173 |
| 9 | 13525082 | rs3900776 | A | G | -0.1100132 | 0.023630171 | 2.75E-06 | 14694 | Anaerostipes | 0.0706 | 0.001472908 | 21.67188845 |
| 3 | 171100102 | rs2014785 | C | T | 0.051568685 | 0.011219863 | 4.68E-06 | 16445 | Anaerostipes | 0.3887 | 0.001282938 | 21.12244576 |
| 2 | 142774333 | rs62157625 | C | T | 0.088573041 | 0.018554786 | 1.45E-06 | 15215 | Anaerostipes | 0.1133 | 0.001495441 | 22.7842183 |
| 2 | 39351569 | rs6726833 | A | C | -0.087749727 | 0.018940832 | 3.32E-06 | 16924 | Anaerostipes | 0.0944 | 0.001266601 | 21.46061199 |
| 9 | 1582701 | rs6474958 | G | A | -0.050006507 | 0.011200088 | 6.74E-06 | 16918 | Anaerostipes | 0.4026 | 0.001176928 | 19.93236625 |
| 4 | 169690814 | rs6854026 | C | T | -0.050848158 | 0.010907505 | 3.20E-06 | 16921 | Anaerostipes | 0.5119 | 0.001282674 | 21.72942643 |
| 19 | 1497547 | rs78735375 | C | A | -0.13742857 | 0.030530436 | 5.33E-06 | 10360 | Anaerostipes | 0.0527 | 0.001952 | 20.25835518 |
| 8 | 128397494 | rs7823228 | C | G | -0.062108471 | 0.013661403 | 5.84E-06 | 16445 | Anaerostipes | 0.2157 | 0.001255254 | 20.66608768 |
| 1 | 67595108 | rs13376554 | T | A | 0.19738899 | 0.04586424 | 7.72E-06 | 3101 | Anaerostipes | 0.0308 | 0.005937578 | 18.51046229 |
| 17 | 2850737 | rs60983350 | A | G | -0.053990065 | 0.011657526 | 4.42E-06 | 16344 | Anaerostipes | 0.4354 | 0.001310651 | 21.44676209 |
| 11 | 105615253 | rs10502061 | G | A | 0.083575226 | 0.019201667 | 7.94E-06 | 16825 | Anaerostipes | 0.0944 | 0.001124692 | 18.94199011 |
| 13 | 38058413 | rs6563550 | C | T | 0.087713486 | 0.017674473 | 2.35E-07 | 16566 | Anaerotruncus | 0.0785 | 0.001484489 | 24.62562983 |
| 14 | 30607199 | rs8005030 | T | C | 0.055444659 | 0.01178543 | 2.28E-06 | 16548 | Anaerotruncus | 0.3022 | 0.00133568 | 22.12972748 |
| 2 | 12200752 | rs4669806 | T | G | 0.057638903 | 0.012299403 | 2.42E-06 | 16566 | Anaerotruncus | 0.2495 | 0.001323946 | 21.95891604 |
| 14 | 30418008 | rs10150232 | G | A | 0.056708789 | 0.012487702 | 6.68E-06 | 16117 | Anaerotruncus | 0.2535 | 0.001277898 | 20.61967554 |
| 9 | 78630894 | rs1272208 | T | G | -0.061174271 | 0.012983073 | 4.28E-06 | 16117 | Anaerotruncus | 0.2803 | 0.001375627 | 22.1987594 |
| 8 | 74713100 | rs12056802 | G | C | 0.077419353 | 0.017717789 | 6.13E-06 | 16010 | Anaerotruncus | 0.0825 | 0.001191163 | 19.09087231 |
| 11 | 89040226 | rs11018566 | G | A | -0.156465176 | 0.036603226 | 6.14E-06 | 6278 | Anaerotruncus | 0.0517 | 0.002902105 | 18.26662399 |
| 4 | 172808992 | rs7675045 | T | A | -0.049781529 | 0.01105734 | 6.92E-06 | 16566 | Anaerotruncus | 0.4722 | 0.001222043 | 20.26668552 |
| 3 | 150855371 | rs1431492 | T | C | -0.06549964 | 0.014618811 | 7.36E-06 | 16560 | Anaerotruncus | 0.1829 | 0.001210786 | 20.07249205 |
| 6 | 165015261 | rs9347879 | C | T | 0.050618001 | 0.011049001 | 4.22E-06 | 16566 | Anaerotruncus | 0.4602 | 0.001265309 | 20.98512364 |
| 2 | 211663702 | rs17734739 | C | T | 0.066005195 | 0.014908026 | 7.43E-06 | 15927 | Anaerotruncus | 0.1899 | 0.001229271 | 19.60024243 |
| 15 | 33459867 | rs6494922 | G | A | 0.090310638 | 0.020225652 | 6.62E-06 | 16067 | Anaerotruncus | 0.0726 | 0.001239365 | 19.93511236 |
| 12 | 76523655 | rs34449434 | C | A | -0.049700388 | 0.011340216 | 9.85E-06 | 16117 | Anaerotruncus | 0.4563 | 0.001190352 | 19.20538468 |
| 14 | 77502546 | rs7155595 | A | C | 0.053933621 | 0.011890292 | 7.55E-06 | 16553 | Anaerotruncus | 0.3091 | 0.001241418 | 20.57224392 |
| 4 | 88163243 | rs115414803 | C | A | -0.144355888 | 0.031752395 | 6.83E-06 | 9273 | Anaerotruncus | 0.0586 | 0.00222397 | 20.66437839 |
| 11 | 54908652 | rs2704155 | A | T | -0.106234694 | 0.023481456 | 5.42E-06 | 12419 | Anaerotruncus | 0.9652 | 0.001645436 | 20.46504719 |
| 3 | 10593224 | rs6795673 | T | C | 0.053856525 | 0.010525127 | 3.38E-07 | 18301 | Bacteroides | 0.4115 | 0.001428651 | 26.18029278 |
| 6 | 29143451 | rs28757219 | A | T | 0.081839827 | 0.017031866 | 1.29E-06 | 14833 | Bacteroides | 0.1521 | 0.001554177 | 23.08587663 |
| 13 | 24910476 | rs9507307 | T | C | 0.060445621 | 0.012912793 | 2.13E-06 | 18184 | Bacteroides | 0.1899 | 0.001203585 | 21.90995734 |
| 20 | 58605432 | rs66474973 | T | G | 0.081251539 | 0.016447899 | 6.81E-07 | 17405 | Bacteroides | 0.1153 | 0.001400102 | 24.40014547 |
| 1 | 10644351 | rs11585893 | G | A | -0.074074561 | 0.014763325 | 1.80E-06 | 17821 | Bacteroides | 0.1481 | 0.001410668 | 25.1721944 |
| 9 | 112855426 | rs495004 | G | C | -0.060747491 | 0.012986536 | 3.42E-06 | 17707 | Bacteroides | 0.2097 | 0.001234209 | 21.87867218 |
| 19 | 24342250 | rs17619981 | G | T | 0.088097761 | 0.018700207 | 2.69E-06 | 15801 | Bacteroides | 0.0656 | 0.001402629 | 22.19125968 |
| 14 | 22045949 | rs2023437 | C | T | -0.07823245 | 0.016763399 | 5.02E-06 | 17484 | Bacteroides | 0.1243 | 0.001244137 | 21.77709746 |
| 3 | 77215327 | rs66710942 | T | C | 0.048803777 | 0.010741209 | 5.86E-06 | 17821 | Bacteroides | 0.4155 | 0.001157086 | 20.64200093 |
| 6 | 41519430 | rs13207588 | G | A | -0.059204982 | 0.013119468 | 7.49E-06 | 17707 | Bacteroides | 0.2326 | 0.001148788 | 20.36268245 |
| 3 | 62019400 | rs2366421 | A | T | -0.05281527 | 0.011712437 | 7.65E-06 | 18299 | Bacteroides | 0.2336 | 0.001109979 | 20.33185624 |
| 1 | 102960989 | rs1340391 | C | T | -0.059200382 | 0.013224416 | 6.73E-06 | 18302 | Bacteroides | 0.1918 | 0.00109376 | 20.0377312 |
| 4 | 5730954 | rs2276875 | G | A | -0.069717565 | 0.013953704 | 4.65E-07 | 13893 | Barnesiella | 0.2545 | 0.001793617 | 24.95990239 |
| 6 | 110831148 | rs2428166 | A | G | -0.165858717 | 0.033730907 | 8.51E-07 | 8091 | Barnesiella | 0.0487 | 0.002979357 | 24.17203421 |
| 12 | 54872438 | rs60316894 | T | C | -0.121478156 | 0.025183972 | 1.19E-06 | 12717 | Barnesiella | 0.0596 | 0.001826288 | 23.26374372 |
| 3 | 181589155 | rs35177866 | G | A | 0.091712545 | 0.019013343 | 2.95E-06 | 13394 | Barnesiella | 0.1243 | 0.001734109 | 23.26353222 |
| 7 | 122021518 | rs13242616 | C | T | -0.058374651 | 0.012317572 | 2.29E-06 | 13945 | Barnesiella | 0.3698 | 0.001607981 | 22.456191 |
| 4 | 114546595 | rs79795328 | G | A | -0.081864467 | 0.01764407 | 4.23E-06 | 13657 | Barnesiella | 0.1252 | 0.001573816 | 21.52433513 |
| 7 | 10076378 | rs2057922 | C | G | 0.091524071 | 0.019462277 | 3.83E-06 | 14208 | Barnesiella | 0.1064 | 0.001554086 | 22.11170412 |
| 6 | 23435415 | rs199035 | A | G | 0.055949483 | 0.011971558 | 3.00E-06 | 13945 | Barnesiella | 0.5089 | 0.001563841 | 21.83878564 |
| 3 | 44327429 | rs62251337 | G | A | -0.069076268 | 0.014937412 | 4.24E-06 | 13945 | Barnesiella | 0.1581 | 0.001531169 | 21.38182247 |
| 5 | 54107036 | rs77455852 | G | T | -0.089149084 | 0.019560057 | 3.16E-06 | 13394 | Barnesiella | 0.1213 | 0.001548496 | 20.76962582 |
| 6 | 148601905 | rs11155559 | C | T | 0.095602282 | 0.021296077 | 8.92E-06 | 13383 | Barnesiella | 0.0954 | 0.001503593 | 20.14987656 |
| 15 | 87145913 | rs12909713 | T | C | -0.055071556 | 0.012003894 | 4.95E-06 | 14205 | Barnesiella | 0.4692 | 0.001479538 | 21.04501417 |
| 2 | 180020849 | rs113258194 | G | A | 0.098992031 | 0.021412093 | 7.31E-06 | 13945 | Barnesiella | 0.1024 | 0.001530378 | 21.370759 |
| 1 | 206216607 | rs28479800 | T | A | -0.091520023 | 0.021000228 | 7.93E-06 | 12399 | Barnesiella | 0.0974 | 0.001529441 | 18.98952737 |
| 8 | 105458416 | rs76181748 | T | C | -0.077855934 | 0.017163996 | 6.78E-06 | 14201 | Barnesiella | 0.1441 | 0.001446768 | 20.57242253 |
| 4 | 102616869 | rs72684847 | C | T | -0.114369278 | 0.025387327 | 6.76E-06 | 12984 | Barnesiella | 0.0656 | 0.001560623 | 20.29167513 |
| 8 | 25834694 | rs28418786 | G | C | -0.079132421 | 0.017495701 | 6.48E-06 | 13698 | Barnesiella | 0.1561 | 0.001491217 | 20.45421413 |
| 2 | 136616754 | rs182549 | T | C | 0.119703495 | 0.012729374 | 1.28E-20 | 14778 | Bifidobacterium | 0.4911 | 0.0059483 | 88.41802126 |
| 13 | 112859829 | rs7322849 | C | T | 0.112428453 | 0.020181262 | 1.08E-08 | 14778 | Bifidobacterium | 0.0964 | 0.0020957 | 31.03109097 |
| 12 | 20378911 | rs10841473 | C | G | -0.062420654 | 0.012943849 | 1.65E-06 | 14777 | Bifidobacterium | 0.2704 | 0.001571305 | 23.25256868 |
| 5 | 537890 | rs56108664 | C | T | 0.072997098 | 0.015792589 | 2.44E-06 | 13916 | Bifidobacterium | 0.1849 | 0.001532935 | 21.36200972 |
| 7 | 48090746 | rs2686790 | C | T | 0.070741011 | 0.015792595 | 7.50E-06 | 14778 | Bifidobacterium | 0.1153 | 0.001355909 | 20.06211734 |
| 2 | 49906864 | rs4567981 | A | T | 0.056208395 | 0.011792309 | 1.93E-06 | 14666 | Bifidobacterium | 0.4394 | 0.001546752 | 22.71671076 |
| 10 | 126089703 | rs2491158 | A | G | 0.071262413 | 0.015983004 | 8.05E-06 | 14021 | Bifidobacterium | 0.1819 | 0.001415826 | 19.87660722 |
| 5 | 169748189 | rs76671854 | G | C | -0.084605457 | 0.01840032 | 3.96E-06 | 13806 | Bifidobacterium | 0.1252 | 0.001529022 | 21.1389348 |
| 21 | 31861790 | rs75344046 | T | C | 0.232354091 | 0.050597867 | 4.86E-06 | 3856 | Bifidobacterium | 0.0497 | 0.005439144 | 21.07710262 |
| 17 | 72897722 | rs540489 | G | T | -0.063764086 | 0.013874591 | 5.19E-06 | 14561 | Bifidobacterium | 0.2336 | 0.001448409 | 21.11797185 |
| 6 | 14617591 | rs857444 | T | C | 0.055823437 | 0.012121924 | 3.57E-06 | 14777 | Bifidobacterium | 0.3608 | 0.001433114 | 21.20465541 |
| 1 | 207003374 | rs12022129 | A | G | 0.061935632 | 0.013893747 | 8.00E-06 | 14776 | Bifidobacterium | 0.2207 | 0.00134308 | 19.86934747 |
| 2 | 189805784 | rs62181700 | A | G | -0.062464331 | 0.013120526 | 2.17E-06 | 14774 | Bifidobacterium | 0.2813 | 0.001531785 | 22.66224344 |
| 4 | 1517826 | rs55888705 | G | A | 0.054631934 | 0.012113865 | 6.67E-06 | 14777 | Bifidobacterium | 0.3867 | 0.001374498 | 20.33615913 |
| 22 | 18354272 | rs5746486 | C | T | -0.053621605 | 0.012080057 | 9.00E-06 | 14778 | Bifidobacterium | 0.3539 | 0.001331519 | 19.70075572 |
| 5 | 142793467 | rs73797465 | G | T | -0.095356633 | 0.02092365 | 4.38E-06 | 14666 | Bifidobacterium | 0.0865 | 0.001414167 | 20.76670896 |
| 6 | 20505628 | rs116261629 | C | G | 0.128112276 | 0.026135828 | 8.62E-07 | 11276 | Bilophila | 0.0716 | 0.002126324 | 24.02325884 |
| 9 | 4940871 | rs1571225 | T | C | 0.082682569 | 0.017062147 | 1.12E-06 | 12854 | Bilophila | 0.1491 | 0.0018236 | 23.47972481 |
| 20 | 61980991 | rs3827020 | T | C | 0.076647909 | 0.016064223 | 1.79E-06 | 12628 | Bilophila | 0.1899 | 0.001799552 | 22.76210418 |
| 1 | 103299921 | rs1241171 | A | G | -0.069265389 | 0.015014825 | 4.24E-06 | 12856 | Bilophila | 0.2157 | 0.0016526 | 21.27768795 |
| 7 | 138583840 | rs7802841 | A | C | 0.067009659 | 0.013770056 | 1.77E-06 | 12856 | Bilophila | 0.3509 | 0.001838647 | 23.67749982 |
| 14 | 67640571 | rs8013541 | T | A | -0.05719913 | 0.01256729 | 5.48E-06 | 12856 | Bilophila | 0.4235 | 0.001608757 | 20.71228404 |
| 3 | 194419531 | rs6793291 | A | C | 0.112729046 | 0.024163294 | 3.11E-06 | 11051 | Bilophila | 0.0924 | 0.001965637 | 21.7610973 |
| 7 | 123174986 | rs1917709 | T | A | 0.118509423 | 0.02674196 | 7.37E-06 | 10886 | Bilophila | 0.0537 | 0.001800811 | 19.63538859 |
| 15 | 50011554 | rs542415 | C | T | -0.061362319 | 0.013343594 | 4.71E-06 | 12777 | Bilophila | 0.34 | 0.001652383 | 21.14413552 |
| 8 | 109990939 | rs72676854 | C | T | 0.123208756 | 0.026882038 | 5.62E-06 | 11616 | Bilophila | 0.0656 | 0.001805167 | 21.003128 |
| 18 | 3765773 | rs4798126 | A | G | 0.073288019 | 0.016798984 | 7.15E-06 | 12777 | Bilophila | 0.1501 | 0.001487389 | 19.02970076 |
| 12 | 100786847 | rs1969927 | A | G | 0.056458855 | 0.012693476 | 9.07E-06 | 12777 | Bilophila | 0.3797 | 0.001545974 | 19.7803982 |
| 8 | 90878338 | rs60178956 | A | G | -0.062481785 | 0.014148499 | 8.06E-06 | 12777 | Bilophila | 0.2346 | 0.001524035 | 19.49926058 |
| 7 | 47211732 | rs2728491 | T | G | -0.062741568 | 0.013945646 | 6.33E-06 | 12856 | Bilophila | 0.3101 | 0.00157197 | 20.23792006 |
| 7 | 9510367 | rs2713349 | T | A | 0.061722789 | 0.014017424 | 8.63E-06 | 12853 | Bilophila | 0.2545 | 0.001506245 | 19.38594874 |
| 17 | 9121579 | rs9899990 | G | A | -0.102702281 | 0.023387821 | 9.07E-06 | 12088 | Bilophila | 0.0656 | 0.0015927 | 19.28007787 |
| 13 | 102402685 | rs11069458 | C | T | -0.068094659 | 0.015502996 | 7.72E-06 | 12856 | Bilophila | 0.1899 | 0.001498434 | 19.28977872 |
| 16 | 77238810 | rs11149971 | T | C | 0.117604747 | 0.023389975 | 1.04E-06 | 16255 | Blautia | 0.0537 | 0.001552846 | 25.27765375 |
| 17 | 16895873 | rs12453000 | T | C | 0.062533456 | 0.012998711 | 1.26E-06 | 17815 | Blautia | 0.2197 | 0.001297402 | 23.14064703 |
| 1 | 175757311 | rs115043014 | A | G | -0.206605438 | 0.043991974 | 5.19E-06 | 4902 | Blautia | 0.0437 | 0.004479335 | 22.04750118 |
| 7 | 124210039 | rs67794373 | T | C | 0.060170889 | 0.012344281 | 1.00E-06 | 18158 | Blautia | 0.2137 | 0.001306789 | 23.75710117 |
| 7 | 68396262 | rs117001700 | C | T | 0.196414252 | 0.044112197 | 8.84E-06 | 4965 | Blautia | 0.0437 | 0.003977211 | 19.81771799 |
| 19 | 1043103 | rs72973581 | G | A | 0.125169075 | 0.026540793 | 1.74E-06 | 12764 | Blautia | 0.0547 | 0.001739496 | 22.23812865 |
| 19 | 13420917 | rs4926264 | C | T | 0.082620681 | 0.017823907 | 5.10E-06 | 18276 | Blautia | 0.0785 | 0.001174303 | 21.48444829 |
| 9 | 1866537 | rs7860714 | G | A | -0.050217775 | 0.010985269 | 4.09E-06 | 18162 | Blautia | 0.334 | 0.001149292 | 20.89516044 |
| 11 | 87805911 | rs682885 | G | A | -0.049337611 | 0.010735883 | 4.49E-06 | 18274 | Blautia | 0.4165 | 0.001154371 | 21.11704399 |
| 1 | 219117743 | rs2788271 | G | T | -0.057559633 | 0.013349169 | 7.16E-06 | 18276 | Blautia | 0.165 | 0.00101626 | 18.59002862 |
| 14 | 78062644 | rs113271346 | T | C | 0.078268222 | 0.017211647 | 6.85E-06 | 17465 | Blautia | 0.1183 | 0.001182616 | 20.67646846 |
| 6 | 74605806 | rs3005511 | G | A | 0.050108496 | 0.01107676 | 6.19E-06 | 18276 | Blautia | 0.336 | 0.001118485 | 20.46207584 |
| 6 | 9298640 | rs16892041 | C | T | -0.062260496 | 0.014180398 | 8.82E-06 | 18276 | Blautia | 0.171 | 0.001053681 | 19.27528415 |
| 2 | 153453410 | rs56221232 | C | T | 0.082802732 | 0.016740066 | 7.62E-07 | 17133 | Butyricicoccus | 0.1083 | 0.001426008 | 24.46382283 |
| 4 | 7460153 | rs2017189 | T | G | -0.050695599 | 0.011024022 | 3.87E-06 | 17133 | Butyricicoccus | 0.4215 | 0.001232796 | 21.14509583 |
| 13 | 100213990 | rs7322368 | C | T | 0.081573314 | 0.018316651 | 5.52E-06 | 17134 | Butyricicoccus | 0.0845 | 0.001156226 | 19.83140071 |
| 18 | 22899113 | rs75238760 | A | T | 0.061942289 | 0.013994184 | 6.80E-06 | 17022 | Butyricicoccus | 0.1938 | 0.001149659 | 19.58972278 |
| 13 | 27018240 | rs12585793 | C | T | -0.262206199 | 0.056472877 | 5.79E-06 | 3135 | Butyricicoccus | 0.0527 | 0.006829554 | 21.54412835 |
| 7 | 157688150 | rs62478070 | G | T | 0.224039318 | 0.049495932 | 5.94E-06 | 4007 | Butyricicoccus | 0.0437 | 0.005087157 | 20.47824088 |
| 10 | 126908208 | rs4962426 | T | G | 0.061421574 | 0.013597912 | 7.38E-06 | 16889 | Butyricicoccus | 0.1978 | 0.001206616 | 20.4007427 |
| 2 | 191304149 | rs10084203 | G | A | 0.054969862 | 0.012356262 | 8.59E-06 | 17135 | Butyricicoccus | 0.2416 | 0.001153691 | 19.7890255 |
| 1 | 67379595 | rs12034718 | G | A | 0.0701199 | 0.01582135 | 9.58E-06 | 16237 | Butyricicoccus | 0.165 | 0.001208272 | 19.64002416 |
| 21 | 16550389 | rs113054641 | A | G | -0.144880972 | 0.027457379 | 1.74E-07 | 9949 | Butyricimonas | 0.0845 | 0.002790689 | 27.83666697 |
| 5 | 159867647 | rs62390301 | C | T | -0.087298337 | 0.017489288 | 7.42E-07 | 10634 | Butyricimonas | 0.1829 | 0.002337516 | 24.91070227 |
| 10 | 72711156 | rs7083431 | C | A | 0.070353941 | 0.014443868 | 8.85E-07 | 10737 | Butyricimonas | 0.3549 | 0.002204795 | 23.72077734 |
| 5 | 62119947 | rs2642760 | C | G | 0.071335713 | 0.014539571 | 8.58E-07 | 10634 | Butyricimonas | 0.3767 | 0.002258563 | 24.06739658 |
| 3 | 127468661 | rs782080 | A | T | 0.065348802 | 0.01379735 | 2.23E-06 | 10733 | Butyricimonas | 0.4066 | 0.00208572 | 22.42864138 |
| 16 | 22172866 | rs1862649 | A | G | 0.113123952 | 0.024817639 | 4.76E-06 | 10123 | Butyricimonas | 0.0915 | 0.002048276 | 20.77315265 |
| 12 | 129438771 | rs12304031 | A | G | -0.086278048 | 0.019684629 | 6.70E-06 | 10658 | Butyricimonas | 0.1292 | 0.001799237 | 19.20722573 |
| 2 | 83093264 | rs71428626 | T | G | -0.133188396 | 0.028990548 | 4.80E-06 | 9776 | Butyricimonas | 0.0726 | 0.002154379 | 21.10236233 |
| 3 | 78740009 | rs78453362 | G | A | -0.14948019 | 0.032707281 | 4.06E-06 | 8207 | Butyricimonas | 0.0577 | 0.002538575 | 20.88202249 |
| 5 | 997217 | rs326049 | G | C | 0.075969365 | 0.01676173 | 8.19E-06 | 10557 | Butyricimonas | 0.2048 | 0.001942024 | 20.5379482 |
| 19 | 49162501 | rs62130338 | A | G | -0.073260812 | 0.015832388 | 3.90E-06 | 10557 | Butyricimonas | 0.2624 | 0.002024091 | 21.4076153 |
| 8 | 4834406 | rs9657374 | T | C | 0.068071943 | 0.014817248 | 4.50E-06 | 10737 | Butyricimonas | 0.3022 | 0.001961848 | 21.10183913 |
| 18 | 34091618 | rs12458763 | C | A | 0.122036628 | 0.026958664 | 6.37E-06 | 10383 | Butyricimonas | 0.0716 | 0.00196972 | 20.48802055 |
| 15 | 80528373 | rs2114713 | T | G | 0.062720962 | 0.013899768 | 6.88E-06 | 10734 | Butyricimonas | 0.4513 | 0.001893327 | 20.35773126 |
| 1 | 45082825 | rs270727 | C | G | -0.069417267 | 0.015079545 | 5.38E-06 | 10634 | Butyricimonas | 0.3221 | 0.001988828 | 21.18736015 |
| 10 | 72443070 | rs72814525 | G | A | 0.066415351 | 0.01497662 | 8.25E-06 | 10736 | Butyricimonas | 0.3231 | 0.001828403 | 19.66203287 |
| 11 | 56664962 | rs11228830 | G | A | 0.135264872 | 0.029841132 | 6.55E-06 | 8974 | Butyricimonas | 0.0706 | 0.002284337 | 20.54199762 |
| 14 | 76065563 | rs72723662 | T | C | 0.224078206 | 0.044933642 | 7.86E-07 | 3073 | Butyrivibrio | 0.1004 | 0.008027734 | 24.85268153 |
| 1 | 235580646 | rs7412979 | G | C | 0.186778577 | 0.038911205 | 1.71E-06 | 3480 | Butyrivibrio | 0.0875 | 0.00657748 | 23.02794108 |
| 6 | 111780705 | rs7752361 | G | A | -0.119230279 | 0.023997488 | 7.69E-07 | 3481 | Butyrivibrio | 0.504 | 0.007041556 | 24.67129589 |
| 13 | 27292666 | rs4537857 | C | T | -0.124589674 | 0.026099163 | 1.80E-06 | 3482 | Butyrivibrio | 0.2922 | 0.006502033 | 22.77515898 |
| 7 | 150596929 | rs11761679 | C | T | 0.154775363 | 0.032153939 | 2.20E-06 | 3482 | Butyrivibrio | 0.1561 | 0.006610376 | 23.15718621 |
| 17 | 20575697 | rs16941336 | T | C | 0.127487508 | 0.026793591 | 1.53E-06 | 3482 | Butyrivibrio | 0.2644 | 0.006459963 | 22.62683912 |
| 18 | 10267733 | rs77356209 | C | T | 0.216874487 | 0.04832994 | 6.66E-06 | 3379 | Butyrivibrio | 0.0477 | 0.00592401 | 20.12460111 |
| 14 | 34150977 | rs74622183 | G | A | -0.201027447 | 0.042820448 | 2.46E-06 | 3478 | Butyrivibrio | 0.0875 | 0.006297024 | 22.02715937 |
| 6 | 21960091 | rs7763512 | A | G | 0.119845684 | 0.025336711 | 3.11E-06 | 3259 | Butyrivibrio | 0.3827 | 0.006818496 | 22.36030488 |
| 10 | 22587900 | rs142855850 | G | A | 0.205013198 | 0.045713757 | 6.86E-06 | 3234 | Butyrivibrio | 0.1014 | 0.006180694 | 20.10023548 |
| 6 | 53939724 | rs9349693 | G | A | 0.117972728 | 0.025986063 | 5.55E-06 | 3482 | Butyrivibrio | 0.3111 | 0.005884241 | 20.59836519 |
| 20 | 811507 | rs486484 | G | A | -0.108348689 | 0.024000793 | 6.61E-06 | 3456 | Butyrivibrio | 0.4712 | 0.005862312 | 20.36782956 |
| 5 | 128976021 | rs17163238 | A | G | 0.140986912 | 0.030911712 | 5.51E-06 | 3482 | Butyrivibrio | 0.1998 | 0.005938761 | 20.79035725 |
| 3 | 54288503 | rs4928024 | G | A | -0.174709994 | 0.038933257 | 8.19E-06 | 3481 | Butyrivibrio | 0.0984 | 0.00575155 | 20.12539576 |
| 6 | 148403405 | rs1007475 | T | G | 0.118065426 | 0.026127086 | 7.92E-06 | 3482 | Butyrivibrio | 0.2942 | 0.005830359 | 20.40863774 |
| 9 | 119693164 | rs16934069 | C | T | -0.133784151 | 0.029944364 | 8.86E-06 | 3482 | Butyrivibrio | 0.1988 | 0.005699908 | 19.94938962 |
| 1 | 224688107 | rs4294381 | C | T | 0.112196526 | 0.023186007 | 1.37E-06 | 6145 | CandidatusSoleaferrea | 0.1799 | 0.003796062 | 23.40806517 |
| 8 | 138638199 | rs10090365 | G | A | -0.083441348 | 0.018098882 | 4.17E-06 | 6145 | CandidatusSoleaferrea | 0.5586 | 0.003446971 | 21.2479847 |
| 3 | 137945192 | rs4678258 | C | T | 0.098613616 | 0.021564627 | 5.53E-06 | 6101 | CandidatusSoleaferrea | 0.2763 | 0.003415882 | 20.90487052 |
| 8 | 123882833 | rs10108780 | G | A | -0.09283404 | 0.01998066 | 3.64E-06 | 5999 | CandidatusSoleaferrea | 0.2674 | 0.003585552 | 21.5799305 |
| 10 | 82879688 | rs386526 | G | C | 0.081845856 | 0.018008617 | 8.33E-06 | 6144 | CandidatusSoleaferrea | 0.5169 | 0.00335061 | 20.64863107 |
| 7 | 352368 | rs36155147 | T | C | 0.104966126 | 0.024099527 | 5.41E-06 | 5958 | CandidatusSoleaferrea | 0.3618 | 0.003173951 | 18.96424203 |
| 5 | 172758774 | rs6881988 | C | G | -0.081913114 | 0.018185036 | 9.23E-06 | 6140 | CandidatusSoleaferrea | 0.4443 | 0.003293649 | 20.28322618 |
| 6 | 109376647 | rs11153159 | C | G | -0.128050003 | 0.028544611 | 4.42E-06 | 6101 | CandidatusSoleaferrea | 0.1292 | 0.003287606 | 20.11724693 |
| 9 | 10611282 | rs10809135 | C | T | 0.08348579 | 0.01824263 | 5.47E-06 | 5999 | CandidatusSoleaferrea | 0.4911 | 0.003479027 | 20.93656356 |
| 2 | 19885907 | rs9973954 | G | A | 0.089207514 | 0.019540149 | 5.95E-06 | 6145 | CandidatusSoleaferrea | 0.3062 | 0.003380296 | 20.83558802 |
| 12 | 115352769 | rs6489992 | G | A | -0.084043749 | 0.018703073 | 7.89E-06 | 6142 | CandidatusSoleaferrea | 0.3847 | 0.003276798 | 20.18568293 |
| 15 | 62394540 | rs6494306 | G | A | -0.09693036 | 0.021422086 | 5.80E-06 | 6145 | CandidatusSoleaferrea | 0.1968 | 0.0033207 | 20.46702636 |
| 4 | 52935445 | rs12500231 | T | A | 0.08138533 | 0.018269552 | 7.68E-06 | 6145 | CandidatusSoleaferrea | 0.4254 | 0.003218951 | 19.83787222 |
| 14 | 76403542 | rs7400877 | C | T | -0.095108487 | 0.021279907 | 9.29E-06 | 6145 | CandidatusSoleaferrea | 0.2505 | 0.00324017 | 19.96906494 |
| 3 | 190349049 | rs2193878 | A | T | 0.228255107 | 0.050924135 | 9.46E-06 | 3088 | CandidatusSoleaferrea | 0.0487 | 0.00646398 | 20.07762419 |
| 19 | 47991441 | rs830149 | G | C | 0.184646616 | 0.039723748 | 9.58E-06 | 5065 | CandidatusSoleaferrea | 0.0586 | 0.004247703 | 21.59786144 |
| 1 | 240118443 | rs12404911 | T | C | 0.140717216 | 0.030414222 | 2.80E-06 | 3239 | Catenibacterium | 0.17 | 0.006565523 | 21.39305396 |
| 4 | 122282768 | rs77285108 | A | G | -0.161826131 | 0.035344674 | 3.63E-06 | 3210 | Catenibacterium | 0.1461 | 0.006488098 | 20.94974112 |
| 7 | 57364320 | rs73128290 | G | A | 0.129726249 | 0.02845626 | 4.29E-06 | 3239 | Catenibacterium | 0.2634 | 0.006375459 | 20.76977684 |
| 6 | 159485742 | rs212393 | A | G | -0.135253856 | 0.02862088 | 3.62E-06 | 3239 | Catenibacterium | 0.3101 | 0.006847598 | 22.31850423 |
| 6 | 105089665 | rs7742829 | T | C | 0.114110133 | 0.025109895 | 5.61E-06 | 3239 | Catenibacterium | 0.4195 | 0.006335595 | 20.63908248 |
| 19 | 34076956 | rs999354 | A | T | 0.058024779 | 0.011718514 | 7.01E-07 | 16324 | ChristensenellaceaeR.7group | 0.325 | 0.001499697 | 24.51482094 |
| 10 | 126448374 | rs78521377 | T | C | 0.12499178 | 0.027479689 | 5.61E-06 | 12605 | ChristensenellaceaeR.7group | 0.0527 | 0.001638642 | 20.68570671 |
| 7 | 118096925 | rs62467127 | T | C | 0.114108279 | 0.025198272 | 3.25E-06 | 12719 | ChristensenellaceaeR.7group | 0.0447 | 0.001609682 | 20.50333495 |
| 18 | 67555560 | rs17081797 | G | A | -0.090432475 | 0.020425314 | 3.34E-06 | 16324 | ChristensenellaceaeR.7group | 0.0686 | 0.001199399 | 19.60009376 |
| 19 | 49279227 | rs62132810 | G | A | -0.082885316 | 0.017962272 | 5.67E-06 | 15066 | ChristensenellaceaeR.7group | 0.1083 | 0.001411306 | 21.28996774 |
| 19 | 35883736 | rs60954665 | G | T | 0.049814958 | 0.011078727 | 7.13E-06 | 16324 | ChristensenellaceaeR.7group | 0.5099 | 0.001237017 | 20.21559802 |
| 4 | 156131004 | rs10461257 | G | A | -0.05519694 | 0.01220692 | 6.51E-06 | 15987 | ChristensenellaceaeR.7group | 0.2853 | 0.001277308 | 20.44388626 |
| 5 | 29352420 | rs79150079 | A | C | 0.121546581 | 0.027097732 | 9.42E-06 | 11994 | ChristensenellaceaeR.7group | 0.0716 | 0.001674664 | 20.11625679 |
| 2 | 230943099 | rs62190261 | C | A | 0.095839425 | 0.021472282 | 8.74E-06 | 15274 | ChristensenellaceaeR.7group | 0.0905 | 0.001302606 | 19.91934847 |
| 9 | 83043376 | rs892686 | G | A | 0.051409887 | 0.011135975 | 3.97E-06 | 16325 | ChristensenellaceaeR.7group | 0.4513 | 0.001303818 | 21.31000582 |
| 18 | 1779609 | rs73952017 | T | C | -0.086216444 | 0.019435393 | 8.46E-06 | 15274 | ChristensenellaceaeR.7group | 0.0875 | 0.001286713 | 19.67599518 |
| 5 | 71186626 | rs6890185 | C | T | 0.113423668 | 0.023313729 | 1.12E-06 | 4166 | Clostridiuminnocuumgroup | 0.3111 | 0.005649424 | 23.65785277 |
| 18 | 6348227 | rs61267978 | C | T | 0.14707953 | 0.032087502 | 5.59E-06 | 4165 | Clostridiuminnocuumgroup | 0.1243 | 0.005019175 | 21.00023052 |
| 3 | 103025517 | rs1948423 | A | T | -0.108859115 | 0.023424976 | 3.49E-06 | 4165 | Clostridiuminnocuumgroup | 0.3082 | 0.005158343 | 21.58552929 |
| 1 | 8419420 | rs6577484 | A | G | 0.16042475 | 0.036085736 | 8.41E-06 | 3858 | Clostridiuminnocuumgroup | 0.1481 | 0.005096714 | 19.75360726 |
| 5 | 113126539 | rs10074000 | C | T | -0.102648064 | 0.022750829 | 7.00E-06 | 4048 | Clostridiuminnocuumgroup | 0.4135 | 0.005003663 | 20.34662751 |
| 18 | 69274308 | rs1942371 | A | G | -0.15793795 | 0.034186957 | 4.06E-06 | 3971 | Clostridiuminnocuumgroup | 0.1302 | 0.005345942 | 21.33208526 |
| 5 | 9368158 | rs40656 | T | C | 0.142664382 | 0.031102116 | 8.62E-06 | 4048 | Clostridiuminnocuumgroup | 0.1889 | 0.005170818 | 21.02987105 |
| 6 | 143718234 | rs71564433 | A | T | -0.126745947 | 0.027465666 | 7.80E-06 | 4164 | Clostridiuminnocuumgroup | 0.1789 | 0.005088172 | 21.2852742 |
| 15 | 59704208 | rs77845139 | G | A | -0.11499293 | 0.02571863 | 8.41E-06 | 4048 | Clostridiuminnocuumgroup | 0.2952 | 0.004914356 | 19.98168042 |
| 12 | 30268485 | rs10506058 | G | A | 0.099704805 | 0.022192573 | 8.92E-06 | 4163 | Clostridiuminnocuumgroup | 0.3966 | 0.004825139 | 20.1747493 |
| 5 | 95717619 | rs4869133 | A | G | -0.180590537 | 0.040950516 | 7.24E-06 | 3735 | Clostridiuminnocuumgroup | 0.0885 | 0.005179945 | 19.43741927 |
| 6 | 165722832 | rs550843 | C | T | -0.078324645 | 0.016920943 | 2.05E-06 | 14102 | Clostridiumsensustricto1 | 0.1491 | 0.001517077 | 21.42328801 |
| 10 | 43270264 | rs2795528 | A | G | -0.184314695 | 0.039220419 | 2.72E-06 | 6366 | Clostridiumsensustricto1 | 0.0487 | 0.003457202 | 22.07796304 |
| 1 | 3041519 | rs2817172 | T | C | 0.058138957 | 0.012449219 | 2.77E-06 | 13658 | Clostridiumsensustricto1 | 0.4125 | 0.001594301 | 21.8065344 |
| 5 | 84997131 | rs115807074 | G | A | -0.227359786 | 0.049257523 | 4.32E-06 | 4229 | Clostridiumsensustricto1 | 0.0497 | 0.005012589 | 21.29495472 |
| 12 | 43485015 | rs116847295 | T | C | 0.110021418 | 0.024603155 | 4.58E-06 | 12910 | Clostridiumsensustricto1 | 0.0755 | 0.001546587 | 19.99427123 |
| 9 | 136710881 | rs12341505 | A | G | 0.081071775 | 0.018012111 | 4.82E-06 | 14118 | Clostridiumsensustricto1 | 0.1113 | 0.001432894 | 20.25576239 |
| 1 | 83252771 | rs11586026 | T | A | 0.110966819 | 0.025049788 | 8.85E-06 | 13095 | Clostridiumsensustricto1 | 0.0636 | 0.001496313 | 19.62057943 |
| 1 | 155676492 | rs11264403 | A | G | -0.139061503 | 0.033445425 | 7.76E-06 | 7285 | Clostridiumsensustricto1 | 0.0398 | 0.002367454 | 17.28308092 |
| 3 | 111482845 | rs12490337 | G | C | -0.061687867 | 0.0137581 | 7.49E-06 | 14121 | Clostridiumsensustricto1 | 0.2455 | 0.001421671 | 20.10115672 |
| 13 | 68610061 | rs9541268 | A | C | 0.09596998 | 0.019731218 | 8.79E-07 | 12914 | Collinsella | 0.0964 | 0.001828552 | 23.65351895 |
| 17 | 47529492 | rs2671662 | G | C | -0.056775091 | 0.011944997 | 2.22E-06 | 14334 | Collinsella | 0.4682 | 0.001573592 | 22.58827038 |
| 2 | 195739311 | rs73052258 | A | G | 0.093017814 | 0.020265842 | 1.72E-06 | 14334 | Collinsella | 0.0964 | 0.001467566 | 21.0640733 |
| 21 | 29355467 | rs2103510 | A | G | 0.078655101 | 0.016821672 | 2.42E-06 | 14334 | Collinsella | 0.1312 | 0.001522952 | 21.86023594 |
| 4 | 149818294 | rs75672793 | G | A | -0.108908003 | 0.024052154 | 6.14E-06 | 12601 | Collinsella | 0.0706 | 0.001624428 | 20.4994731 |
| 11 | 107123199 | rs10890671 | C | T | -0.053740144 | 0.011884842 | 6.52E-06 | 14334 | Collinsella | 0.5268 | 0.001424375 | 20.44326184 |
| 16 | 77283458 | rs12921100 | T | A | 0.056375862 | 0.012701892 | 8.23E-06 | 14334 | Collinsella | 0.3171 | 0.001372416 | 19.69649679 |
| 15 | 50026113 | rs59414781 | G | C | 0.066886563 | 0.014977291 | 9.15E-06 | 14334 | Collinsella | 0.1799 | 0.001389441 | 19.94116885 |
| 7 | 24465024 | rs62448871 | A | C | -0.054030922 | 0.012032414 | 6.78E-06 | 14334 | Collinsella | 0.4612 | 0.001404757 | 20.16130594 |
| 19 | 31760841 | rs1496626 | C | T | -0.072230674 | 0.016160046 | 6.78E-06 | 14334 | Collinsella | 0.1541 | 0.001391829 | 19.97549562 |
| 19 | 56526640 | rs149807560 | A | C | -0.104264209 | 0.023584097 | 7.10E-06 | 13473 | Collinsella | 0.0895 | 0.001448565 | 19.54193326 |
| 10 | 8589813 | rs11597285 | T | G | -0.053794651 | 0.012054849 | 9.38E-06 | 14334 | Collinsella | 0.4245 | 0.001387345 | 19.91104524 |
| 1 | 88233128 | rs305411 | G | A | 0.129218483 | 0.026451784 | 1.01E-06 | 6777 | Coprobacter | 0.1352 | 0.003508934 | 23.85674296 |
| 3 | 46483717 | rs3828477 | T | G | -0.091217951 | 0.019569041 | 2.89E-06 | 6798 | Coprobacter | 0.2813 | 0.003186064 | 21.72169761 |
| 4 | 34852443 | rs55672356 | A | T | -0.193354203 | 0.041381488 | 2.74E-06 | 4903 | Coprobacter | 0.0527 | 0.00443306 | 21.82316991 |
| 11 | 103549974 | rs143662916 | T | C | 0.25326389 | 0.05401412 | 3.07E-06 | 3083 | Coprobacter | 0.0577 | 0.007080638 | 21.97101447 |
| 6 | 97234939 | rs213863 | T | C | -0.088741189 | 0.018837727 | 2.35E-06 | 6798 | Coprobacter | 0.3091 | 0.003253845 | 22.18532056 |
| 6 | 4714797 | rs72821405 | C | T | -0.147365313 | 0.031997805 | 4.76E-06 | 6177 | Coprobacter | 0.0885 | 0.00342203 | 21.20359606 |
| 12 | 98289796 | rs11532348 | T | C | -0.103930603 | 0.022682638 | 5.71E-06 | 6364 | Coprobacter | 0.1829 | 0.003288056 | 20.98762336 |
| 2 | 137223458 | rs12996055 | C | A | 0.092181727 | 0.020940499 | 8.08E-06 | 6570 | Coprobacter | 0.1918 | 0.002940839 | 19.37239885 |
| 9 | 137784868 | rs12684609 | C | T | 0.100826957 | 0.022036643 | 6.10E-06 | 6364 | Coprobacter | 0.1978 | 0.003278733 | 20.92791439 |
| 15 | 91166608 | rs5011652 | C | G | 0.089959749 | 0.02000097 | 5.51E-06 | 6783 | Coprobacter | 0.2455 | 0.002973577 | 20.22396411 |
| 2 | 32242452 | rs74919520 | A | G | 0.125702917 | 0.02762957 | 5.76E-06 | 6387 | Coprobacter | 0.1282 | 0.003230282 | 20.69218964 |
| 4 | 96389240 | rs28402691 | C | T | 0.110695393 | 0.025119913 | 9.56E-06 | 6387 | Coprobacter | 0.167 | 0.003031151 | 19.41273948 |
| 20 | 57574851 | rs189356 | A | G | 0.078112515 | 0.017191637 | 6.26E-06 | 6763 | Coprobacter | 0.5219 | 0.003043292 | 20.63850854 |
| 4 | 3852385 | rs76001613 | G | C | 0.215910371 | 0.049301859 | 9.13E-06 | 3385 | Coprobacter | 0.0696 | 0.005633886 | 19.16742399 |
| 20 | 4319420 | rs4277593 | A | G | -0.058564915 | 0.010991363 | 1.14E-07 | 16651 | Coprococcus1 | 0.4831 | 0.001702126 | 28.38701751 |
| 4 | 174468316 | rs56405618 | G | A | -0.089630001 | 0.018650815 | 1.57E-06 | 16379 | Coprococcus1 | 0.1113 | 0.00140803 | 23.09181807 |
| 1 | 95424321 | rs74101919 | C | T | -0.071898851 | 0.014465115 | 1.03E-06 | 16732 | Coprococcus1 | 0.1869 | 0.001474387 | 24.70291533 |
| 1 | 30400301 | rs1010560 | A | C | 0.058023212 | 0.012272151 | 1.96E-06 | 16723 | Coprococcus1 | 0.2962 | 0.001334959 | 22.35168047 |
| 11 | 134622282 | rs73031725 | C | T | 0.167590996 | 0.035522381 | 1.98E-06 | 7206 | Coprococcus1 | 0.0606 | 0.003079382 | 22.25239059 |
| 2 | 22079770 | rs1519491 | C | T | 0.049919694 | 0.01135578 | 8.95E-06 | 16733 | Coprococcus1 | 0.4085 | 0.001153546 | 19.3222632 |
| 6 | 72529277 | rs1576241 | G | A | -0.051034349 | 0.010953456 | 3.33E-06 | 16729 | Coprococcus1 | 0.4394 | 0.001295954 | 21.70555622 |
| 3 | 165693143 | rs73167075 | C | T | 0.057313703 | 0.01275731 | 8.57E-06 | 16733 | Coprococcus1 | 0.2396 | 0.001204762 | 20.18119284 |
| 11 | 124554061 | rs12794898 | T | G | 0.090329854 | 0.019721215 | 4.92E-06 | 15186 | Coprococcus1 | 0.0944 | 0.001379597 | 20.9767437 |
| 10 | 15913820 | rs946513 | T | C | 0.205898923 | 0.046001796 | 8.62E-06 | 4310 | Coprococcus1 | 0.0547 | 0.004626657 | 20.02428412 |
| 6 | 150288094 | rs1762123 | T | C | -0.089153231 | 0.019855409 | 8.01E-06 | 16724 | Coprococcus1 | 0.0716 | 0.001204074 | 20.15879337 |
| 7 | 132556835 | rs7784490 | G | C | -0.051854255 | 0.011313593 | 4.55E-06 | 16733 | Coprococcus1 | 0.3887 | 0.00125386 | 21.00466624 |
| 14 | 29898456 | rs12886051 | C | G | -0.052178208 | 0.011805063 | 8.01E-06 | 16633 | Coprococcus1 | 0.3429 | 0.001173171 | 19.53392391 |
| 19 | 2600717 | rs2907920 | G | A | 0.056117333 | 0.012682384 | 7.65E-06 | 16040 | Coprococcus1 | 0.2903 | 0.001219153 | 19.57663672 |
| 20 | 49266770 | rs59936925 | T | A | 0.117085317 | 0.023399473 | 9.38E-07 | 10931 | Coprococcus2 | 0.1004 | 0.002285279 | 25.03301943 |
| 1 | 112139008 | rs6677933 | T | C | -0.080440499 | 0.016421626 | 1.19E-06 | 11483 | Coprococcus2 | 0.2416 | 0.002085236 | 23.99062319 |
| 14 | 86833255 | rs1958519 | A | T | 0.066528846 | 0.013855806 | 1.58E-06 | 11009 | Coprococcus2 | 0.4364 | 0.002089777 | 23.05034962 |
| 4 | 131125786 | rs72680320 | C | T | -0.064938201 | 0.013918999 | 2.27E-06 | 11009 | Coprococcus2 | 0.4135 | 0.001973235 | 21.76233911 |
| 9 | 25554068 | rs2482516 | T | C | 0.075441464 | 0.016461865 | 4.72E-06 | 11483 | Coprococcus2 | 0.2256 | 0.001825632 | 20.99841371 |
| 10 | 129715759 | rs35890118 | G | A | -0.066536003 | 0.014766106 | 8.26E-06 | 11478 | Coprococcus2 | 0.3171 | 0.001765825 | 20.30045547 |
| 1 | 4210455 | rs9426473 | G | A | 0.072736094 | 0.016166159 | 6.31E-06 | 11486 | Coprococcus2 | 0.2793 | 0.001759352 | 20.2400066 |
| 3 | 180544880 | rs12634070 | C | T | 0.073648589 | 0.016492134 | 9.95E-06 | 11457 | Coprococcus2 | 0.1928 | 0.001737597 | 19.9388141 |
| 1 | 223688236 | rs61823518 | C | A | -0.095538071 | 0.021572431 | 6.68E-06 | 10980 | Coprococcus2 | 0.0954 | 0.001783107 | 19.60991104 |
| 5 | 34794789 | rs10070053 | G | A | 0.059431172 | 0.01353752 | 7.65E-06 | 11473 | Coprococcus2 | 0.4076 | 0.001677044 | 19.26968621 |
| 19 | 40032132 | rs8100692 | C | T | 0.057741093 | 0.011347595 | 4.16E-07 | 15829 | Coprococcus3 | 0.4274 | 0.001633048 | 25.88853531 |
| 8 | 4128340 | rs62481985 | C | G | -0.058238523 | 0.011505221 | 4.18E-07 | 15472 | Coprococcus3 | 0.4364 | 0.001653353 | 25.61973586 |
| 22 | 21331556 | rs178271 | C | T | 0.145256923 | 0.029423868 | 7.81E-07 | 10794 | Coprococcus3 | 0.0527 | 0.002252746 | 24.3665288 |
| 2 | 240727657 | rs13394391 | T | C | -0.070930158 | 0.015091785 | 2.20E-06 | 15834 | Coprococcus3 | 0.16 | 0.001393107 | 22.08644456 |
| 17 | 26104511 | rs11080344 | T | C | 0.051692096 | 0.011305408 | 4.79E-06 | 15835 | Coprococcus3 | 0.4742 | 0.001318514 | 20.90359507 |
| 1 | 149998497 | rs7521171 | A | G | -0.05964344 | 0.012927857 | 4.32E-06 | 15472 | Coprococcus3 | 0.338 | 0.001373817 | 21.28218057 |
| 17 | 76343961 | rs11077359 | C | T | -0.064544278 | 0.014883314 | 9.64E-06 | 15378 | Coprococcus3 | 0.1998 | 0.001221478 | 18.8044109 |
| 7 | 76357726 | rs13247359 | A | G | 0.051210989 | 0.011303088 | 7.33E-06 | 15833 | Coprococcus3 | 0.4801 | 0.00129481 | 20.52471897 |
| 14 | 99141751 | rs4575475 | A | G | 0.061951375 | 0.013778722 | 7.04E-06 | 15154 | Coprococcus3 | 0.2366 | 0.001332226 | 20.21280944 |
| 9 | 13799809 | rs10810043 | G | A | 0.051551783 | 0.011592302 | 9.27E-06 | 15833 | Coprococcus3 | 0.3897 | 0.001247506 | 19.77393651 |
| 3 | 72207794 | rs4677103 | G | A | 0.09779121 | 0.019726332 | 9.60E-07 | 7983 | DefluviitaleaceaeUCG011 | 0.171 | 0.003069064 | 24.56960222 |
| 4 | 147414743 | rs72731813 | T | C | -0.147382972 | 0.029379613 | 4.33E-07 | 7767 | DefluviitaleaceaeUCG011 | 0.0815 | 0.003229567 | 25.15884117 |
| 9 | 8786663 | rs112893842 | C | T | 0.113810202 | 0.023280562 | 1.45E-06 | 8183 | DefluviitaleaceaeUCG011 | 0.0974 | 0.002912034 | 23.89292539 |
| 1 | 85205632 | rs9725395 | G | A | -0.138339669 | 0.029554002 | 3.52E-06 | 8011 | DefluviitaleaceaeUCG011 | 0.0875 | 0.002727645 | 21.90546216 |
| 21 | 41811003 | rs55658617 | C | T | 0.174368775 | 0.036222554 | 2.15E-06 | 6663 | DefluviitaleaceaeUCG011 | 0.0696 | 0.003465786 | 23.16588664 |
| 22 | 24804081 | rs9608282 | G | T | 0.14293919 | 0.029979298 | 2.52E-06 | 7716 | DefluviitaleaceaeUCG011 | 0.0656 | 0.002937581 | 22.72726205 |
| 7 | 79734541 | rs28696126 | T | A | -0.106502748 | 0.023847256 | 6.63E-06 | 7811 | DefluviitaleaceaeUCG011 | 0.1918 | 0.00254701 | 19.94038632 |
| 1 | 118723685 | rs1582238 | C | T | 0.080503588 | 0.016725672 | 1.57E-06 | 7983 | DefluviitaleaceaeUCG011 | 0.3718 | 0.002893601 | 23.16085035 |
| 10 | 66407366 | rs4344384 | T | G | 0.071597782 | 0.015630912 | 4.83E-06 | 8180 | DefluviitaleaceaeUCG011 | 0.4742 | 0.002558378 | 20.97608018 |
| 4 | 120741726 | rs2892880 | A | G | 0.081775542 | 0.018168647 | 6.83E-06 | 8182 | DefluviitaleaceaeUCG011 | 0.2604 | 0.002469837 | 20.25328671 |
| 14 | 97116904 | rs11160353 | A | T | -0.069175564 | 0.014728505 | 2.70E-06 | 9234 | Desulfovibrio | 0.508 | 0.002383208 | 22.05433627 |
| 8 | 105462047 | rs2853179 | T | C | 0.081197078 | 0.017423443 | 2.42E-06 | 8949 | Desulfovibrio | 0.2485 | 0.002420949 | 21.71279643 |
| 2 | 198089911 | rs16863365 | G | A | 0.109402491 | 0.022696375 | 1.79E-06 | 8776 | Desulfovibrio | 0.1173 | 0.002640563 | 23.22963836 |
| 1 | 68664315 | rs12031543 | C | T | -0.127184982 | 0.028188722 | 6.55E-06 | 8855 | Desulfovibrio | 0.0755 | 0.002293692 | 20.35273524 |
| 3 | 67478973 | rs13066142 | A | G | 0.11914059 | 0.025089055 | 3.79E-06 | 9241 | Desulfovibrio | 0.0934 | 0.002434296 | 22.54534537 |
| 13 | 64264446 | rs2590913 | A | G | 0.154495517 | 0.033939625 | 6.65E-06 | 7225 | Desulfovibrio | 0.0726 | 0.002859803 | 20.71559723 |
| 10 | 130170270 | rs2032031 | G | A | -0.065493057 | 0.014868084 | 9.14E-06 | 9241 | Desulfovibrio | 0.4732 | 0.002095321 | 19.39931981 |
| 8 | 57972916 | rs72647089 | G | T | -0.10659436 | 0.023933668 | 8.30E-06 | 8985 | Desulfovibrio | 0.1064 | 0.002202795 | 19.83139413 |
| 5 | 52653978 | rs7729080 | A | C | -0.070286961 | 0.015769108 | 9.96E-06 | 9241 | Desulfovibrio | 0.2893 | 0.002145278 | 19.86283692 |
| 18 | 13447995 | rs4797774 | A | G | 0.21258194 | 0.04700444 | 5.64E-06 | 3476 | Desulfovibrio | 0.0686 | 0.005849883 | 20.44207631 |
| 5 | 139410687 | rs6580353 | C | T | 0.0770979 | 0.016973529 | 4.94E-06 | 9241 | Desulfovibrio | 0.2604 | 0.002227682 | 20.62750883 |
| 8 | 138295725 | rs11166701 | A | G | -0.065530102 | 0.013186 | 5.51E-07 | 11720 | Dialister | 0.5159 | 0.002102876 | 24.69343205 |
| 11 | 10967286 | rs517089 | A | T | 0.076227029 | 0.017041108 | 5.14E-06 | 11988 | Dialister | 0.169 | 0.001666293 | 20.00552108 |
| 10 | 23199653 | rs4747450 | A | C | 0.066850856 | 0.014768536 | 5.84E-06 | 11987 | Dialister | 0.2664 | 0.001706423 | 20.48644412 |
| 16 | 9336862 | rs2314294 | C | T | 0.086593192 | 0.019371895 | 8.08E-06 | 11339 | Dialister | 0.1183 | 0.001759073 | 19.97775513 |
| 11 | 92267129 | rs4753063 | A | G | -0.059629704 | 0.013005185 | 4.86E-06 | 11989 | Dialister | 0.4414 | 0.001750445 | 21.01937321 |
| 7 | 150890034 | rs2435610 | C | A | 0.064713149 | 0.014331424 | 5.93E-06 | 11984 | Dialister | 0.2604 | 0.001698503 | 20.38608822 |
| 4 | 23294359 | rs10938938 | A | G | -0.077351446 | 0.017091491 | 7.37E-06 | 11401 | Dialister | 0.1938 | 0.001793307 | 20.47862565 |
| 14 | 102458052 | rs10138457 | C | T | -0.113053794 | 0.026193253 | 7.88E-06 | 10968 | Dialister | 0.0716 | 0.001695614 | 18.62568253 |
| 15 | 32955095 | rs11071887 | C | T | 0.066240302 | 0.014631484 | 5.91E-06 | 11989 | Dialister | 0.2634 | 0.001706644 | 20.49252056 |
| 3 | 45809449 | rs764177 | A | C | -0.060145359 | 0.013536587 | 9.61E-06 | 11988 | Dialister | 0.338 | 0.001644086 | 19.73846577 |
| 9 | 25447080 | rs76680460 | A | G | -0.16129611 | 0.036433211 | 8.19E-06 | 7176 | Dialister | 0.0477 | 0.002723867 | 19.59439404 |
| 1 | 172809633 | rs75416973 | G | A | 0.072722304 | 0.016450983 | 9.46E-06 | 11402 | Dialister | 0.2256 | 0.001710909 | 19.53778569 |
| 8 | 127905865 | rs13279148 | A | G | 0.071530899 | 0.015087753 | 2.25E-06 | 17605 | Dorea | 0.166 | 0.001275111 | 22.47443958 |
| 8 | 15768657 | rs62503162 | G | A | -0.09740913 | 0.019437924 | 7.47E-07 | 16210 | Dorea | 0.0875 | 0.001546836 | 25.10995665 |
| 6 | 116078810 | rs12216169 | A | T | 0.088172222 | 0.019368315 | 5.33E-06 | 16086 | Dorea | 0.0835 | 0.001286686 | 20.72172518 |
| 6 | 25078050 | rs73729431 | T | C | -0.137450296 | 0.029998324 | 3.17E-06 | 9596 | Dorea | 0.0606 | 0.002183022 | 20.98972992 |
| 17 | 70733523 | rs4793307 | T | C | 0.057416514 | 0.012247163 | 4.01E-06 | 17600 | Dorea | 0.2455 | 0.001247234 | 21.9762339 |
| 16 | 81803497 | rs11150408 | G | T | 0.04882539 | 0.010906337 | 7.06E-06 | 17215 | Dorea | 0.4453 | 0.001162843 | 20.03932658 |
| 3 | 6826520 | rs345219 | G | T | -0.049736844 | 0.011262081 | 8.80E-06 | 17215 | Dorea | 0.3757 | 0.001131672 | 19.5015313 |
| 9 | 106815466 | rs62583469 | A | T | -0.063413594 | 0.014222695 | 5.78E-06 | 16819 | Dorea | 0.1988 | 0.001180559 | 19.87692839 |
| 6 | 74605806 | rs3005511 | G | A | 0.051612659 | 0.011285226 | 5.29E-06 | 17610 | Dorea | 0.336 | 0.001186362 | 20.91426607 |
| 4 | 60992340 | rs1899291 | T | C | 0.069721719 | 0.015028462 | 4.57E-06 | 17609 | Dorea | 0.1282 | 0.001220793 | 21.52076957 |
| 11 | 8729057 | rs3752849 | A | G | 0.16384587 | 0.036607992 | 7.68E-06 | 5322 | Dorea | 0.0328 | 0.00374984 | 20.02423556 |
| 7 | 154978183 | rs12537781 | C | T | -0.055544008 | 0.012522298 | 9.15E-06 | 17608 | Dorea | 0.2296 | 0.001116121 | 19.67238486 |
| 2 | 45453805 | rs3851328 | G | T | -0.107774251 | 0.023652669 | 4.18E-06 | 5147 | Eggerthella | 0.2664 | 0.004017605 | 20.75395699 |
| 7 | 38335954 | rs2240838 | G | A | 0.098058185 | 0.01976528 | 7.36E-07 | 5147 | Eggerthella | 0.502 | 0.004759219 | 24.60327634 |
| 21 | 29778032 | rs2223081 | A | G | 0.102571659 | 0.022110762 | 3.89E-06 | 5147 | Eggerthella | 0.2922 | 0.004163716 | 21.51188904 |
| 1 | 9136139 | rs112205261 | C | T | -0.188617927 | 0.040361319 | 3.35E-06 | 5036 | Eggerthella | 0.0577 | 0.004317877 | 21.83045198 |
| 17 | 17098213 | rs4985746 | A | G | 0.110522311 | 0.024765265 | 5.71E-06 | 4906 | Eggerthella | 0.1789 | 0.004043216 | 19.90842318 |
| 11 | 102461599 | rs1784446 | A | G | 0.090772774 | 0.019809579 | 5.23E-06 | 5147 | Eggerthella | 0.4423 | 0.004062922 | 20.98900883 |
| 20 | 55647869 | rs76663501 | T | C | 0.175349437 | 0.03785764 | 4.83E-06 | 4814 | Eggerthella | 0.0815 | 0.004436745 | 21.44476103 |
| 2 | 141337454 | rs6430926 | T | C | 0.08797364 | 0.019712748 | 8.37E-06 | 5147 | Eggerthella | 0.5139 | 0.0038546 | 19.90865907 |
| 4 | 70483957 | rs2877457 | A | G | -0.093486043 | 0.021020752 | 9.03E-06 | 5083 | Eggerthella | 0.8678 | 0.003876059 | 19.77088753 |
| 3 | 20807454 | rs13070736 | C | A | -0.12133107 | 0.027202382 | 7.62E-06 | 5147 | Eggerthella | 0.1571 | 0.003850353 | 19.88663909 |
| 15 | 65691324 | rs67490567 | C | T | 0.108494846 | 0.024545741 | 8.94E-06 | 5083 | Eggerthella | 0.1978 | 0.00382895 | 19.52967526 |
| 8 | 50822676 | rs3812426 | A | G | 0.106447316 | 0.0224159 | 2.72E-06 | 5583 | Eisenbergiella | 0.2078 | 0.004022896 | 22.54246771 |
| 15 | 53375201 | rs1508033 | C | A | 0.091545961 | 0.019578652 | 3.23E-06 | 5580 | Eisenbergiella | 0.3499 | 0.003902836 | 21.85531696 |
| 11 | 124764527 | rs12278566 | A | T | -0.121064706 | 0.025165804 | 1.65E-06 | 5582 | Eisenbergiella | 0.175 | 0.004128828 | 23.13437917 |
| 15 | 36178565 | rs2683098 | T | C | 0.107318939 | 0.022515029 | 2.24E-06 | 5582 | Eisenbergiella | 0.2237 | 0.00405372 | 22.71182714 |
| 17 | 53367641 | rs11079158 | C | T | 0.100626785 | 0.022546032 | 7.35E-06 | 5582 | Eisenbergiella | 0.2197 | 0.003555904 | 19.91275364 |
| 8 | 55330814 | rs13258851 | G | A | 0.137001443 | 0.030212434 | 7.75E-06 | 5583 | Eisenbergiella | 0.0855 | 0.003669565 | 20.55527284 |
| 2 | 19973210 | rs12710729 | A | C | 0.089338392 | 0.019906928 | 9.84E-06 | 5583 | Eisenbergiella | 0.3708 | 0.003594482 | 20.13317056 |
| 10 | 109417101 | rs12257723 | C | A | -0.095274183 | 0.021156892 | 8.85E-06 | 5582 | Eisenbergiella | 0.339 | 0.003619778 | 20.27174324 |
| 21 | 21610254 | rs4462860 | A | G | 0.093907092 | 0.020110429 | 4.16E-06 | 5460 | Eisenbergiella | 0.3439 | 0.003977686 | 21.79691322 |
| 11 | 24001840 | rs11027642 | T | C | 0.129005538 | 0.028484392 | 4.92E-06 | 5583 | Eisenbergiella | 0.1243 | 0.003660518 | 20.50440536 |
| 4 | 189060431 | rs11938607 | C | T | 0.097812525 | 0.021663562 | 8.22E-06 | 5583 | Eisenbergiella | 0.2634 | 0.003638134 | 20.37856488 |
| 3 | 111419486 | rs1553971 | G | T | 0.120964524 | 0.026305423 | 5.27E-06 | 5583 | Eisenbergiella | 0.161 | 0.003773254 | 21.13829047 |
| 4 | 127336602 | rs11098863 | A | T | -0.096600831 | 0.016267701 | 3.06E-09 | 7524 | Enterorhabdus | 0.4821 | 0.004664767 | 35.25282356 |
| 10 | 20280612 | rs7923280 | T | A | 0.086001907 | 0.016914834 | 5.24E-07 | 7524 | Enterorhabdus | 0.3946 | 0.003424067 | 25.84432572 |
| 2 | 19611953 | rs114731706 | G | T | 0.182308046 | 0.038229783 | 2.17E-06 | 5956 | Enterorhabdus | 0.0835 | 0.00380363 | 22.73328257 |
| 11 | 62174193 | rs3017103 | G | A | 0.098089701 | 0.020899456 | 2.94E-06 | 7524 | Enterorhabdus | 0.1899 | 0.002919164 | 22.0222354 |
| 12 | 69515102 | rs73331712 | C | T | 0.261989512 | 0.055123019 | 4.85E-06 | 3109 | Enterorhabdus | 0.0427 | 0.00721335 | 22.5747188 |
| 2 | 236439879 | rs77655283 | A | G | 0.132987641 | 0.029830551 | 5.88E-06 | 7494 | Enterorhabdus | 0.0686 | 0.002645063 | 19.86936993 |
| 6 | 37567189 | rs9470637 | T | A | -0.075551295 | 0.016626296 | 5.70E-06 | 7524 | Enterorhabdus | 0.4026 | 0.002736868 | 20.64321674 |
| 19 | 35863028 | rs424715 | C | T | 0.081874254 | 0.017485462 | 4.41E-06 | 7524 | Enterorhabdus | 0.5537 | 0.002905548 | 21.91921641 |
| 8 | 113109756 | rs10098492 | C | T | 0.132323148 | 0.029368842 | 6.41E-06 | 7361 | Enterorhabdus | 0.0905 | 0.002750205 | 20.29457593 |
| 7 | 89709883 | rs2051957 | T | C | 0.084329062 | 0.018986405 | 8.90E-06 | 7524 | Enterorhabdus | 0.2346 | 0.002615069 | 19.72212084 |
| 17 | 10177708 | rs7221249 | G | A | 0.083986052 | 0.01427414 | 4.31E-09 | 9781 | Erysipelatoclostridium | 0.5467 | 0.003526929 | 34.61191341 |
| 1 | 42333631 | rs710230 | C | T | 0.143381274 | 0.028151388 | 6.33E-07 | 9016 | Erysipelatoclostridium | 0.0865 | 0.002868954 | 25.93515833 |
| 4 | 25446787 | rs4697572 | G | A | -0.081063643 | 0.016330822 | 7.59E-07 | 9779 | Erysipelatoclostridium | 0.2455 | 0.002513326 | 24.63470253 |
| 11 | 121876913 | rs58236560 | T | G | -0.11115688 | 0.023442572 | 2.16E-06 | 9368 | Erysipelatoclostridium | 0.1372 | 0.002394276 | 22.47860955 |
| 9 | 90586043 | rs622418 | G | A | -0.066805108 | 0.014322449 | 3.68E-06 | 9783 | Erysipelatoclostridium | 0.5109 | 0.002218952 | 21.75183811 |
| 5 | 77815056 | rs17804233 | C | T | -0.066291401 | 0.014420319 | 4.59E-06 | 9783 | Erysipelatoclostridium | 0.4821 | 0.002155537 | 21.12885167 |
| 2 | 34595533 | rs1434153 | A | G | -0.06845264 | 0.01524432 | 6.85E-06 | 9662 | Erysipelatoclostridium | 0.3211 | 0.002082533 | 20.15924963 |
| 5 | 34892042 | rs340991 | G | A | -0.074048187 | 0.015889138 | 3.75E-06 | 9781 | Erysipelatoclostridium | 0.2982 | 0.00221555 | 21.71397573 |
| 8 | 38780606 | rs6474512 | C | A | 0.067014995 | 0.014320143 | 3.02E-06 | 9783 | Erysipelatoclostridium | 0.4513 | 0.002233604 | 21.89578242 |
| 13 | 46372065 | rs9590927 | A | G | -0.064600302 | 0.014347949 | 6.39E-06 | 9782 | Erysipelatoclostridium | 0.494 | 0.002068058 | 20.26752008 |
| 1 | 167818597 | rs61806970 | T | C | 0.142547998 | 0.032068815 | 9.09E-06 | 8644 | Erysipelatoclostridium | 0.0646 | 0.002280605 | 19.7540401 |
| 19 | 55857777 | rs45480394 | G | T | -0.068713443 | 0.015183535 | 7.66E-06 | 9604 | Erysipelatoclostridium | 0.3618 | 0.002127945 | 20.47609564 |
| 11 | 36871523 | rs2901723 | A | C | 0.064133865 | 0.014429456 | 8.79E-06 | 9662 | Erysipelatoclostridium | 0.503 | 0.002040426 | 19.75081446 |
| 17 | 71411325 | rs34528142 | G | C | -0.087555073 | 0.019555627 | 6.13E-06 | 9604 | Erysipelatoclostridium | 0.171 | 0.002082867 | 20.04142862 |
| 10 | 36394609 | rs16936671 | T | C | -0.096802556 | 0.021788273 | 6.04E-06 | 9488 | Erysipelatoclostridium | 0.1362 | 0.002076112 | 19.73497049 |
| 6 | 24292185 | rs3804326 | G | A | 0.141469838 | 0.033635154 | 9.85E-06 | 7028 | Erysipelatoclostridium | 0.0676 | 0.00251083 | 17.68549698 |
| 10 | 125628540 | rs28568391 | G | A | -0.058391081 | 0.011874052 | 6.42E-07 | 14544 | ErysipelotrichaceaeUCG003 | 0.4493 | 0.00165993 | 24.17883875 |
| 8 | 91660344 | rs11994308 | T | C | 0.115417413 | 0.024250252 | 1.33E-06 | 13558 | ErysipelotrichaceaeUCG003 | 0.0696 | 0.001667975 | 22.64884635 |
| 15 | 86522244 | rs76502207 | C | T | 0.14484376 | 0.028992913 | 6.41E-07 | 10740 | ErysipelotrichaceaeUCG003 | 0.0905 | 0.002318481 | 24.95370202 |
| 18 | 46614602 | rs10164067 | G | T | -0.103260131 | 0.021240502 | 1.13E-06 | 14427 | ErysipelotrichaceaeUCG003 | 0.0746 | 0.001635494 | 23.63064556 |
| 16 | 4076830 | rs8053479 | G | A | -0.083821185 | 0.018650912 | 5.83E-06 | 13813 | ErysipelotrichaceaeUCG003 | 0.1412 | 0.001460108 | 20.19503239 |
| 7 | 33684926 | rs17798136 | A | G | 0.15881755 | 0.034767431 | 3.24E-06 | 7421 | ErysipelotrichaceaeUCG003 | 0.0616 | 0.002803948 | 20.86098064 |
| 18 | 43034126 | rs59104037 | G | A | -0.095126261 | 0.020474209 | 4.48E-06 | 14848 | ErysipelotrichaceaeUCG003 | 0.0815 | 0.001451736 | 21.58380931 |
| 19 | 57828980 | rs11666127 | G | A | -0.07191871 | 0.016137058 | 7.90E-06 | 13813 | ErysipelotrichaceaeUCG003 | 0.171 | 0.001435896 | 19.85967543 |
| 6 | 24220160 | rs62403464 | C | T | -0.073174326 | 0.015651572 | 3.44E-06 | 14899 | ErysipelotrichaceaeUCG003 | 0.164 | 0.001464899 | 21.85461796 |
| 4 | 113256737 | rs59068084 | G | T | 0.056477243 | 0.012022387 | 3.12E-06 | 14544 | ErysipelotrichaceaeUCG003 | 0.4215 | 0.001515037 | 22.06509838 |
| 7 | 20554814 | rs73074432 | T | C | 0.072153999 | 0.016437785 | 9.99E-06 | 14217 | ErysipelotrichaceaeUCG003 | 0.166 | 0.001353437 | 19.26518161 |
| 11 | 7765096 | rs4758231 | T | G | -0.05517544 | 0.012204675 | 6.55E-06 | 14890 | ErysipelotrichaceaeUCG003 | 0.3499 | 0.00137072 | 20.43529083 |
| 16 | 80167277 | rs74988980 | A | G | -0.133141406 | 0.034857439 | 8.64E-06 | 5944 | ErysipelotrichaceaeUCG003 | 0.0437 | 0.002448454 | 14.58442082 |
| 2 | 175403951 | rs75949021 | C | T | -0.16966706 | 0.037423793 | 3.58E-06 | 6721 | ErysipelotrichaceaeUCG003 | 0.0457 | 0.003048874 | 20.54803057 |
| 5 | 32345671 | rs6875357 | T | C | 0.165673441 | 0.035383116 | 6.70E-06 | 7034 | ErysipelotrichaceaeUCG003 | 0.0467 | 0.003107132 | 21.91745494 |
| 14 | 47935928 | rs79396538 | G | C | 0.084814752 | 0.019114638 | 8.63E-06 | 13750 | ErysipelotrichaceaeUCG003 | 0.1034 | 0.001429837 | 19.68554619 |
| 10 | 873148 | rs12251396 | G | A | -0.070539949 | 0.015876598 | 9.52E-06 | 14211 | ErysipelotrichaceaeUCG003 | 0.1938 | 0.001387164 | 19.73759678 |
| 7 | 31136897 | rs2267739 | C | G | 0.115505392 | 0.02376277 | 1.42E-06 | 11853 | Escherichia.Shigella | 0.0855 | 0.001989378 | 23.62311186 |
| 11 | 134774845 | rs1154904 | G | A | -0.061337641 | 0.01306443 | 3.04E-06 | 12002 | Escherichia.Shigella | 0.4642 | 0.001833254 | 22.03945534 |
| 21 | 39324484 | rs73208162 | G | A | -0.119295648 | 0.024838994 | 2.19E-06 | 11073 | Escherichia.Shigella | 0.0586 | 0.002078797 | 23.06230778 |
| 7 | 140678199 | rs113513883 | G | A | 0.172283306 | 0.038041024 | 5.28E-06 | 6280 | Escherichia.Shigella | 0.0577 | 0.003255414 | 20.50423904 |
| 17 | 14173290 | rs7502686 | C | G | -0.136383738 | 0.030257643 | 5.90E-06 | 9990 | Escherichia.Shigella | 0.0626 | 0.002029585 | 20.31271792 |
| 2 | 236515155 | rs57024273 | C | T | 0.062617236 | 0.013991866 | 9.70E-06 | 11853 | Escherichia.Shigella | 0.3181 | 0.001686845 | 20.02457253 |
| 13 | 23253392 | rs113127095 | G | A | 0.151009395 | 0.032343436 | 3.33E-06 | 8433 | Escherichia.Shigella | 0.0517 | 0.002578293 | 21.7937819 |
| 10 | 69394914 | rs117092367 | T | A | 0.11736182 | 0.026453289 | 9.65E-06 | 10304 | Escherichia.Shigella | 0.0885 | 0.0019066 | 19.67931698 |
| 16 | 3750623 | rs112767262 | C | T | 0.073296333 | 0.01635886 | 8.21E-06 | 11771 | Escherichia.Shigella | 0.1958 | 0.00170257 | 20.07172219 |
| 3 | 3173016 | rs11706043 | A | T | 0.07565695 | 0.016415509 | 5.87E-06 | 12001 | Escherichia.Shigella | 0.1571 | 0.001766866 | 21.23814416 |
| 9 | 136179347 | rs592299 | C | T | -0.059200433 | 0.012948643 | 4.77E-06 | 11995 | Escherichia.Shigella | 0.4682 | 0.001739582 | 20.89916006 |
| 7 | 128054100 | rs4731451 | A | G | -0.060988547 | 0.013515708 | 7.47E-06 | 12001 | Escherichia.Shigella | 0.3718 | 0.001693812 | 20.35853742 |
| 16 | 77711641 | rs35555519 | G | C | 0.101975959 | 0.022288306 | 4.92E-06 | 12002 | Escherichia.Shigella | 0.1143 | 0.001741129 | 20.92999413 |
| 5 | 79863544 | rs118526 | A | C | -0.059440114 | 0.013598005 | 8.00E-06 | 11853 | Escherichia.Shigella | 0.3926 | 0.001609463 | 19.10449497 |
| 1 | 48961903 | rs2798105 | G | A | -0.100850689 | 0.02220448 | 8.25E-06 | 11296 | Escherichia.Shigella | 0.1183 | 0.001822886 | 20.6252697 |
| 10 | 94185213 | rs112617308 | C | T | -0.170873361 | 0.036284397 | 2.38E-06 | 4001 | Eubacteriumbrachygroup | 0.0795 | 0.005512387 | 22.166226 |
| 22 | 26922534 | rs9613196 | A | T | -0.238718472 | 0.052856104 | 4.99E-06 | 3004 | Eubacteriumbrachygroup | 0.0765 | 0.006744393 | 20.38414613 |
| 5 | 17460096 | rs62348779 | C | T | -0.201480569 | 0.043286015 | 3.78E-06 | 3827 | Eubacteriumbrachygroup | 0.0815 | 0.005629385 | 21.65429762 |
| 10 | 22892667 | rs2913110 | T | C | 0.105143062 | 0.022941973 | 4.56E-06 | 4001 | Eubacteriumbrachygroup | 0.3777 | 0.005222245 | 20.99339006 |
| 4 | 184628931 | rs4862235 | A | G | 0.104806298 | 0.022575135 | 3.73E-06 | 4000 | Eubacteriumbrachygroup | 0.4443 | 0.00535945 | 21.54253806 |
| 2 | 218237281 | rs12151423 | G | A | 0.101328627 | 0.022732445 | 9.27E-06 | 3854 | Eubacteriumbrachygroup | 0.4791 | 0.005128934 | 19.85850685 |
| 4 | 6856564 | rs73199919 | C | T | -0.236716837 | 0.053133485 | 8.16E-06 | 3008 | Eubacteriumbrachygroup | 0.0567 | 0.006555225 | 19.83502861 |
| 11 | 80236833 | rs6591893 | A | G | 0.108225435 | 0.024031859 | 7.34E-06 | 3999 | Eubacteriumbrachygroup | 0.3479 | 0.005045865 | 20.27060446 |
| 22 | 48190598 | rs720439 | G | A | -0.111927002 | 0.025125245 | 7.03E-06 | 4001 | Eubacteriumbrachygroup | 0.2515 | 0.004935507 | 19.83498953 |
| 4 | 144665178 | rs13139592 | C | T | -0.145996616 | 0.032719209 | 7.97E-06 | 3842 | Eubacteriumbrachygroup | 0.1223 | 0.005155583 | 19.90003532 |
| 14 | 23060552 | rs1384962 | G | A | 0.120888267 | 0.026626427 | 6.99E-06 | 4001 | Eubacteriumbrachygroup | 0.2107 | 0.005125571 | 20.60275789 |
| 7 | 31085162 | rs17159861 | T | C | 0.096222758 | 0.016838828 | 1.04E-08 | 17380 | Eubacteriumcoprostanoligenesgroup | 0.1332 | 0.001875283 | 32.64990366 |
| 7 | 16380806 | rs9648214 | C | T | -0.082871942 | 0.016430987 | 2.52E-07 | 17379 | Eubacteriumcoprostanoligenesgroup | 0.1183 | 0.001461597 | 25.43535217 |
| 1 | 200638489 | rs115325767 | C | G | 0.078332188 | 0.017471885 | 2.62E-06 | 16555 | Eubacteriumcoprostanoligenesgroup | 0.0934 | 0.001212676 | 20.09779899 |
| 15 | 36911598 | rs12906958 | T | C | -0.053315925 | 0.011593477 | 4.35E-06 | 17380 | Eubacteriumcoprostanoligenesgroup | 0.3022 | 0.00121537 | 21.14640478 |
| 15 | 86440997 | rs4076415 | G | T | 0.051515202 | 0.011029435 | 1.99E-06 | 16960 | Eubacteriumcoprostanoligenesgroup | 0.4006 | 0.001284636 | 21.81288408 |
| 3 | 127139574 | rs6762473 | A | C | 0.052158365 | 0.011235251 | 4.26E-06 | 17377 | Eubacteriumcoprostanoligenesgroup | 0.3569 | 0.001238709 | 21.54926314 |
| 10 | 2215931 | rs10444197 | G | A | -0.050582278 | 0.011345529 | 5.98E-06 | 17380 | Eubacteriumcoprostanoligenesgroup | 0.3678 | 0.001142355 | 19.87455079 |
| 10 | 84506096 | rs2644213 | A | G | 0.053884526 | 0.012124458 | 9.86E-06 | 17380 | Eubacteriumcoprostanoligenesgroup | 0.2366 | 0.001135169 | 19.74938256 |
| 3 | 113794507 | rs11720857 | T | C | 0.063077578 | 0.014448482 | 9.26E-06 | 16960 | Eubacteriumcoprostanoligenesgroup | 0.1769 | 0.001122514 | 19.05699219 |
| 7 | 73306506 | rs4717831 | T | A | 0.078678267 | 0.017403801 | 9.18E-06 | 16854 | Eubacteriumcoprostanoligenesgroup | 0.1193 | 0.001211134 | 20.43477294 |
| 15 | 97869293 | rs62024432 | T | C | -0.076969763 | 0.017210135 | 7.50E-06 | 17271 | Eubacteriumcoprostanoligenesgroup | 0.1252 | 0.001156782 | 19.99960658 |
| 3 | 81595477 | rs76898927 | A | G | 0.12305502 | 0.026637491 | 4.79E-06 | 12197 | Eubacteriumcoprostanoligenesgroup | 0.0477 | 0.001746625 | 21.33736625 |
| 7 | 33603146 | rs1020520 | G | T | -0.059070472 | 0.013292872 | 8.89E-06 | 17371 | Eubacteriumcoprostanoligenesgroup | 0.2038 | 0.001135494 | 19.74482315 |
| 12 | 32713919 | rs11052069 | C | T | 0.047783175 | 0.01078333 | 9.38E-06 | 17380 | Eubacteriumcoprostanoligenesgroup | 0.4344 | 0.001128507 | 19.63334369 |
| 2 | 50797401 | rs79895140 | C | T | -0.06410167 | 0.014115288 | 8.62E-06 | 16139 | Eubacteriumcoprostanoligenesgroup | 0.2167 | 0.001276227 | 20.62079484 |
| 16 | 81820486 | rs4583233 | C | A | 0.067020477 | 0.012812178 | 2.84E-07 | 14327 | Eubacteriumeligensgroup | 0.334 | 0.001906273 | 27.35952013 |
| 10 | 83092007 | rs265534 | G | T | -0.056375608 | 0.01201272 | 2.27E-06 | 14776 | Eubacteriumeligensgroup | 0.4553 | 0.001488319 | 22.02120204 |
| 2 | 115197171 | rs72839198 | G | C | 0.155658995 | 0.037561222 | 3.14E-06 | 4926 | Eubacteriumeligensgroup | 0.0557 | 0.003474267 | 17.16693075 |
| 14 | 39301409 | rs74606150 | G | C | -0.196453545 | 0.042564951 | 4.25E-06 | 5381 | Eubacteriumeligensgroup | 0.0457 | 0.003943089 | 21.29384136 |
| 6 | 70956815 | rs6923695 | G | T | 0.103267603 | 0.02295245 | 4.87E-06 | 12891 | Eubacteriumeligensgroup | 0.0567 | 0.001567841 | 20.23963622 |
| 2 | 110260993 | rs56080211 | T | C | 0.123092697 | 0.028274315 | 9.14E-06 | 10699 | Eubacteriumeligensgroup | 0.0557 | 0.001768351 | 18.94956336 |
| 14 | 77619186 | rs158115 | C | G | 0.091527053 | 0.019061087 | 9.74E-06 | 14678 | Eubacteriumeligensgroup | 0.0924 | 0.001568394 | 23.05390144 |
| 13 | 107563458 | rs2200429 | G | A | -0.088882459 | 0.019847898 | 5.30E-06 | 14761 | Eubacteriumeligensgroup | 0.0736 | 0.001356743 | 20.0513795 |
| 11 | 31098691 | rs182318 | A | G | -0.082494113 | 0.019561497 | 8.40E-06 | 14777 | Eubacteriumeligensgroup | 0.0775 | 0.001202079 | 17.78209671 |
| 7 | 51365152 | rs12719051 | G | A | 0.091662637 | 0.020818348 | 7.12E-06 | 12799 | Eubacteriumeligensgroup | 0.1004 | 0.001512373 | 19.38314773 |
| 2 | 71145246 | rs3771393 | T | C | 0.130842344 | 0.026667104 | 7.38E-07 | 3792 | Eubacteriumfissicatenagroup | 0.2843 | 0.00630853 | 24.06111745 |
| 8 | 5433699 | rs2733072 | A | G | 0.109644168 | 0.022830587 | 1.49E-06 | 3833 | Eubacteriumfissicatenagroup | 0.4652 | 0.00598126 | 23.05208776 |
| 11 | 115165111 | rs7104872 | A | G | 0.138611743 | 0.029190553 | 2.73E-06 | 3829 | Eubacteriumfissicatenagroup | 0.1779 | 0.005854369 | 22.53660603 |
| 7 | 73043561 | rs151257695 | G | A | 0.209509779 | 0.045484723 | 3.10E-06 | 3587 | Eubacteriumfissicatenagroup | 0.0805 | 0.005880102 | 21.20485364 |
| 18 | 45753272 | rs11876297 | C | T | 0.131469398 | 0.028171203 | 2.67E-06 | 3396 | Eubacteriumfissicatenagroup | 0.2157 | 0.006372276 | 21.76620483 |
| 6 | 39940437 | rs6934739 | G | A | 0.111463384 | 0.025278805 | 9.75E-06 | 3831 | Eubacteriumfissicatenagroup | 0.2952 | 0.005049411 | 19.43231708 |
| 14 | 89484373 | rs10147907 | G | T | 0.172262807 | 0.039600983 | 8.27E-06 | 3833 | Eubacteriumfissicatenagroup | 0.0686 | 0.004912399 | 18.91230436 |
| 3 | 39604954 | rs1768152 | C | T | 0.139488571 | 0.031618584 | 8.70E-06 | 3590 | Eubacteriumfissicatenagroup | 0.1372 | 0.005392 | 19.45137927 |
| 10 | 96758467 | rs11818408 | A | G | 0.10585035 | 0.023711404 | 8.20E-06 | 3590 | Eubacteriumfissicatenagroup | 0.4771 | 0.005520412 | 19.91718962 |
| 4 | 111885431 | rs13116360 | C | T | 0.154124445 | 0.029718533 | 2.94E-07 | 10323 | Eubacteriumhalliigroup | 0.0626 | 0.002598677 | 26.8908273 |
| 3 | 110283389 | rs949971 | G | T | -0.054016364 | 0.011610071 | 3.29E-06 | 16566 | Eubacteriumhalliigroup | 0.3837 | 0.001304957 | 21.64355122 |
| 1 | 34308917 | rs10798999 | T | C | 0.060166074 | 0.012675245 | 2.61E-06 | 16561 | Eubacteriumhalliigroup | 0.2157 | 0.001358669 | 22.52880672 |
| 7 | 148856720 | rs60254196 | G | A | -0.052283163 | 0.011186491 | 2.70E-06 | 16238 | Eubacteriumhalliigroup | 0.5408 | 0.001343444 | 21.84149992 |
| 7 | 100635375 | rs10808115 | C | A | -0.050474741 | 0.010992248 | 4.42E-06 | 16566 | Eubacteriumhalliigroup | 0.4781 | 0.001271174 | 21.08253165 |
| 15 | 62014160 | rs74018587 | T | C | 0.208942799 | 0.043821974 | 3.70E-06 | 3039 | Eubacteriumhalliigroup | 0.0368 | 0.007425121 | 22.71878181 |
| 3 | 23663416 | rs6550770 | C | T | -0.198087408 | 0.044354487 | 4.82E-06 | 4279 | Eubacteriumhalliigroup | 0.0517 | 0.004639559 | 19.93588742 |
| 11 | 58040621 | rs10501370 | T | C | -0.115588894 | 0.025248889 | 5.42E-06 | 13824 | Eubacteriumhalliigroup | 0.0527 | 0.001513757 | 20.95486408 |
| 19 | 49214274 | rs281379 | G | A | -0.049952328 | 0.011215293 | 9.33E-06 | 16130 | Eubacteriumhalliigroup | 0.4602 | 0.00122835 | 19.83519094 |
| 11 | 123789877 | rs78056098 | T | G | -0.050741114 | 0.011375822 | 8.29E-06 | 16568 | Eubacteriumhalliigroup | 0.3608 | 0.001199398 | 19.89308018 |
| 3 | 195238086 | rs138531890 | G | A | 0.153138471 | 0.034945641 | 5.43E-06 | 7710 | Eubacteriumhalliigroup | 0.0537 | 0.002484551 | 19.19861538 |
| 3 | 64664456 | rs28584818 | G | A | 0.126115364 | 0.026862747 | 4.43E-06 | 11095 | Eubacteriumhalliigroup | 0.0686 | 0.001982649 | 22.03722182 |
| 11 | 11771637 | rs117748144 | C | T | -0.126581958 | 0.028711955 | 7.86E-06 | 10861 | Eubacteriumhalliigroup | 0.0427 | 0.001786371 | 19.43292179 |
| 1 | 104524676 | rs17474256 | A | G | 0.081080509 | 0.018457309 | 9.45E-06 | 16460 | Eubacteriumhalliigroup | 0.1233 | 0.001171002 | 19.29493955 |
| 18 | 48384463 | rs630939 | T | C | -0.050891423 | 0.011435211 | 9.16E-06 | 16238 | Eubacteriumhalliigroup | 0.3996 | 0.001218257 | 19.80374657 |
| 4 | 183709536 | rs17074066 | C | T | -0.081398624 | 0.018927232 | 9.35E-06 | 15804 | Eubacteriumhalliigroup | 0.0915 | 0.00116892 | 18.4928981 |
| 14 | 52185888 | rs34297067 | G | A | -0.186906548 | 0.034147492 | 6.60E-08 | 3271 | Eubacteriumnodatumgroup | 0.1392 | 0.009075932 | 29.94096515 |
| 9 | 28245518 | rs113893692 | T | C | -0.18510716 | 0.040357325 | 5.76E-06 | 3195 | Eubacteriumnodatumgroup | 0.0984 | 0.006541548 | 21.02469668 |
| 9 | 96597985 | rs77910827 | T | C | 0.201788441 | 0.041356826 | 9.05E-07 | 3271 | Eubacteriumnodatumgroup | 0.1034 | 0.007225505 | 23.79208722 |
| 10 | 9659749 | rs61841040 | T | G | 0.160621141 | 0.034160929 | 3.56E-06 | 3295 | Eubacteriumnodatumgroup | 0.166 | 0.00666479 | 22.09440899 |
| 1 | 34529736 | rs9425984 | C | T | -0.130233832 | 0.029234653 | 7.21E-06 | 3294 | Eubacteriumnodatumgroup | 0.2386 | 0.005988521 | 19.83297975 |
| 7 | 64894137 | rs10263623 | T | C | 0.193495904 | 0.043902338 | 8.91E-06 | 3080 | Eubacteriumnodatumgroup | 0.1044 | 0.006267395 | 19.41270965 |
| 10 | 61322015 | rs11006576 | G | A | -0.110167094 | 0.024592963 | 7.99E-06 | 3295 | Eubacteriumnodatumgroup | 0.5129 | 0.006053266 | 20.0548018 |
| 1 | 171060821 | rs7880204 | C | T | -0.125453808 | 0.027530312 | 6.84E-06 | 3294 | Eubacteriumnodatumgroup | 0.2475 | 0.006264589 | 20.75303675 |
| 4 | 94712747 | rs6818880 | G | A | -0.110080596 | 0.024596445 | 7.83E-06 | 3291 | Eubacteriumnodatumgroup | 0.4553 | 0.00604942 | 20.01763839 |
| 7 | 135052348 | rs10458299 | C | T | -0.187752781 | 0.041969011 | 8.37E-06 | 3271 | Eubacteriumnodatumgroup | 0.0805 | 0.006081149 | 20.00090592 |
| 8 | 4954912 | rs7827125 | T | C | 0.122299815 | 0.027133143 | 7.17E-06 | 3294 | Eubacteriumnodatumgroup | 0.2823 | 0.006129956 | 20.30428115 |
| 12 | 94515340 | rs12423772 | T | G | 0.140979641 | 0.029502889 | 2.63E-06 | 5321 | Eubacteriumoxidoreducensgroup | 0.1501 | 0.004272979 | 22.82550746 |
| 4 | 37526679 | rs2973294 | T | G | 0.092355871 | 0.019543871 | 2.39E-06 | 5378 | Eubacteriumoxidoreducensgroup | 0.4205 | 0.004135114 | 22.32267811 |
| 16 | 86480355 | rs34561138 | A | G | 0.216145345 | 0.04599197 | 2.51E-06 | 4186 | Eubacteriumoxidoreducensgroup | 0.0586 | 0.005248595 | 22.07599106 |
| 5 | 106882394 | rs440215 | T | C | 0.093276592 | 0.019529391 | 1.65E-06 | 5377 | Eubacteriumoxidoreducensgroup | 0.4622 | 0.004224636 | 22.80375606 |
| 4 | 187905399 | rs1425962 | C | G | 0.090610394 | 0.020061232 | 7.32E-06 | 5321 | Eubacteriumoxidoreducensgroup | 0.3429 | 0.003819317 | 20.39283232 |
| 1 | 195064019 | rs12129908 | A | C | 0.08932725 | 0.019833287 | 5.80E-06 | 5377 | Eubacteriumoxidoreducensgroup | 0.4473 | 0.003758401 | 20.27761942 |
| 15 | 93006765 | rs35398954 | G | A | -0.090140156 | 0.017460171 | 5.40E-07 | 16621 | Eubacteriumrectalegroup | 0.1213 | 0.001600983 | 26.64939539 |
| 12 | 20066025 | rs117151453 | C | G | -0.113140609 | 0.02435414 | 1.84E-06 | 13908 | Eubacteriumrectalegroup | 0.0507 | 0.001549364 | 21.57888332 |
| 18 | 37703874 | rs16960159 | G | C | -0.156595964 | 0.03362338 | 4.10E-06 | 8334 | Eubacteriumrectalegroup | 0.0477 | 0.002595948 | 21.68573619 |
| 2 | 125553155 | rs314726 | C | T | 0.052882354 | 0.010948624 | 1.38E-06 | 16966 | Eubacteriumrectalegroup | 0.4573 | 0.001373176 | 23.32659159 |
| 1 | 234530370 | rs10797540 | G | A | 0.050317907 | 0.010838946 | 3.53E-06 | 17350 | Eubacteriumrectalegroup | 0.4344 | 0.001240602 | 21.54869466 |
| 7 | 121281238 | rs10248854 | A | C | -0.052782677 | 0.01134635 | 4.21E-06 | 17348 | Eubacteriumrectalegroup | 0.3598 | 0.00124589 | 21.63816267 |
| 1 | 170900221 | rs3980709 | T | A | -0.062270145 | 0.014057159 | 6.86E-06 | 17340 | Eubacteriumrectalegroup | 0.161 | 0.00113038 | 19.62070233 |
| 11 | 8129537 | rs2884897 | G | A | -0.129363699 | 0.028902068 | 6.44E-06 | 10813 | Eubacteriumrectalegroup | 0.0527 | 0.001849341 | 20.03026453 |
| 11 | 98523638 | rs10892089 | G | C | -0.063552174 | 0.014007233 | 6.22E-06 | 17351 | Eubacteriumrectalegroup | 0.1968 | 0.001184995 | 20.58287506 |
| 1 | 116082909 | rs143694765 | C | T | 0.087002523 | 0.01977896 | 9.75E-06 | 15124 | Eubacteriumrectalegroup | 0.0944 | 0.001277718 | 19.34636488 |
| 9 | 129425745 | rs59427698 | G | A | -0.057603509 | 0.013085337 | 5.37E-06 | 16613 | Eubacteriumrectalegroup | 0.2028 | 0.001165128 | 19.37652025 |
| 9 | 92013419 | rs62547233 | G | A | 0.0536319 | 0.012020063 | 9.90E-06 | 17347 | Eubacteriumrectalegroup | 0.3072 | 0.001146332 | 19.9059468 |
| 5 | 121182996 | rs2116427 | G | A | 0.091146051 | 0.018235307 | 4.67E-07 | 7739 | Eubacteriumruminantiumgroup | 0.2565 | 0.003217844 | 24.97682999 |
| 22 | 25303055 | rs139749 | T | C | -0.084543927 | 0.017179078 | 8.59E-07 | 7314 | Eubacteriumruminantiumgroup | 0.3419 | 0.00330046 | 24.21287643 |
| 10 | 129037453 | rs72836424 | T | C | -0.139824589 | 0.030068963 | 2.62E-06 | 7314 | Eubacteriumruminantiumgroup | 0.0954 | 0.002947767 | 21.61779623 |
| 9 | 130980937 | rs2229917 | G | A | 0.153537929 | 0.032392215 | 2.16E-06 | 7050 | Eubacteriumruminantiumgroup | 0.0666 | 0.003176721 | 22.46088395 |
| 14 | 52219066 | rs10131724 | C | A | -0.199832279 | 0.041457713 | 2.39E-06 | 5071 | Eubacteriumruminantiumgroup | 0.0636 | 0.004560807 | 23.22465519 |
| 5 | 33897589 | rs16891896 | A | G | -0.174787011 | 0.039057268 | 2.38E-06 | 4753 | Eubacteriumruminantiumgroup | 0.0586 | 0.004195857 | 20.01851229 |
| 8 | 138626268 | rs7000472 | G | A | -0.076228208 | 0.016523022 | 4.07E-06 | 7739 | Eubacteriumruminantiumgroup | 0.3618 | 0.002742678 | 21.27846164 |
| 2 | 201610645 | rs13025464 | C | T | -0.073707715 | 0.016378577 | 6.97E-06 | 7738 | Eubacteriumruminantiumgroup | 0.3827 | 0.002610416 | 20.24702795 |
| 2 | 71247578 | rs2418654 | T | C | -0.074887891 | 0.016585161 | 6.17E-06 | 7314 | Eubacteriumruminantiumgroup | 0.4781 | 0.002779841 | 20.38285686 |
| 1 | 197978281 | rs6676699 | T | G | -0.088812364 | 0.019644717 | 6.38E-06 | 7708 | Eubacteriumruminantiumgroup | 0.2217 | 0.002644622 | 20.43349219 |
| 12 | 63306003 | rs73139629 | C | A | -0.115097848 | 0.024790941 | 5.36E-06 | 7314 | Eubacteriumruminantiumgroup | 0.1322 | 0.00293843 | 21.54912359 |
| 22 | 48749548 | rs112375806 | A | T | 0.143141085 | 0.029375749 | 5.82E-06 | 7123 | Eubacteriumruminantiumgroup | 0.1074 | 0.003322328 | 23.7371593 |
| 9 | 2539358 | rs606117 | G | A | 0.083324266 | 0.018055987 | 4.82E-06 | 7263 | Eubacteriumruminantiumgroup | 0.332 | 0.002923566 | 21.29025794 |
| 6 | 130030114 | rs57340348 | C | T | -0.097942933 | 0.021216595 | 4.93E-06 | 7314 | Eubacteriumruminantiumgroup | 0.2147 | 0.002905202 | 21.30473186 |
| 1 | 3044181 | rs2817174 | T | C | -0.073430579 | 0.016368685 | 7.87E-06 | 7710 | Eubacteriumruminantiumgroup | 0.4294 | 0.002603393 | 20.11933496 |
| 1 | 88523399 | rs10923018 | A | G | 0.072643786 | 0.016092406 | 6.80E-06 | 7739 | Eubacteriumruminantiumgroup | 0.4722 | 0.002626202 | 20.37242628 |
| 15 | 61289622 | rs11637981 | T | G | -0.073257996 | 0.016088796 | 5.44E-06 | 7718 | Eubacteriumruminantiumgroup | 0.4841 | 0.002679126 | 20.72766706 |
| 12 | 30360954 | rs17519472 | T | C | 0.10780414 | 0.023398378 | 4.70E-06 | 7710 | Eubacteriumruminantiumgroup | 0.1541 | 0.002745686 | 21.22201999 |
| 6 | 11953633 | rs209813 | A | G | -0.103488116 | 0.023639057 | 9.23E-06 | 7264 | Eubacteriumruminantiumgroup | 0.166 | 0.002631482 | 19.16024517 |
| 20 | 2308307 | rs6048195 | T | A | -0.060359432 | 0.011657994 | 2.50E-07 | 14839 | Eubacteriumventriosumgroup | 0.4791 | 0.001803243 | 26.80304277 |
| 20 | 50140919 | rs73615400 | C | T | -0.095630317 | 0.01934276 | 9.54E-07 | 15146 | Eubacteriumventriosumgroup | 0.0905 | 0.001611224 | 24.43976158 |
| 3 | 190223732 | rs57199565 | C | T | 0.078339435 | 0.01602571 | 7.97E-07 | 14913 | Eubacteriumventriosumgroup | 0.2406 | 0.001599801 | 23.89285522 |
| 13 | 98785900 | rs11617697 | G | A | -0.143348528 | 0.028624073 | 7.22E-07 | 11497 | Eubacteriumventriosumgroup | 0.0477 | 0.002176671 | 25.07541099 |
| 8 | 114445055 | rs16884680 | T | G | -0.090642843 | 0.019190901 | 1.74E-06 | 15289 | Eubacteriumventriosumgroup | 0.1074 | 0.001457015 | 22.30588553 |
| 18 | 22460785 | rs12964517 | A | G | 0.058719806 | 0.012345332 | 2.07E-06 | 15289 | Eubacteriumventriosumgroup | 0.3161 | 0.001477551 | 22.62074119 |
| 12 | 52423996 | rs876734 | T | C | -0.061869648 | 0.013253231 | 2.89E-06 | 14913 | Eubacteriumventriosumgroup | 0.2893 | 0.001459192 | 21.78981186 |
| 14 | 44975510 | rs3809430 | C | T | -0.054817114 | 0.011842566 | 3.55E-06 | 15287 | Eubacteriumventriosumgroup | 0.3479 | 0.00139962 | 21.42317911 |
| 16 | 60348381 | rs72783037 | A | C | 0.065892572 | 0.014374684 | 6.55E-06 | 15286 | Eubacteriumventriosumgroup | 0.1869 | 0.001372732 | 21.00968309 |
| 4 | 126371814 | rs73849225 | C | T | 0.097571148 | 0.022438827 | 5.21E-06 | 14573 | Eubacteriumventriosumgroup | 0.0795 | 0.001295778 | 18.90527116 |
| 13 | 51938576 | rs9316536 | G | T | -0.081711077 | 0.018312095 | 7.84E-06 | 15289 | Eubacteriumventriosumgroup | 0.1362 | 0.001300593 | 19.9080619 |
| 14 | 51960625 | rs35179274 | T | C | -0.062726329 | 0.013785975 | 5.76E-06 | 15287 | Eubacteriumventriosumgroup | 0.2435 | 0.00135243 | 20.69988554 |
| 1 | 147291928 | rs78250280 | A | G | 0.074976242 | 0.016440448 | 3.36E-06 | 14835 | Eubacteriumventriosumgroup | 0.1491 | 0.001399987 | 20.79512413 |
| 1 | 109398264 | rs66746423 | T | C | 0.0751501 | 0.016497843 | 6.11E-06 | 14578 | Eubacteriumventriosumgroup | 0.1561 | 0.001421311 | 20.74651746 |
| 2 | 203661779 | rs6704822 | G | A | 0.073917922 | 0.016670495 | 6.62E-06 | 15289 | Eubacteriumventriosumgroup | 0.1163 | 0.001284297 | 19.65828818 |
| 3 | 107736434 | rs13082419 | T | C | -0.071552205 | 0.016136969 | 9.56E-06 | 14501 | Eubacteriumventriosumgroup | 0.17 | 0.001353991 | 19.65813129 |
| 3 | 171895485 | rs66830358 | A | T | 0.052875209 | 0.011797227 | 6.87E-06 | 14913 | Eubacteriumventriosumgroup | 0.4175 | 0.001345224 | 20.08565991 |
| 20 | 57995506 | rs17830032 | A | G | -0.160597431 | 0.031051453 | 2.39E-07 | 9527 | Eubacteriumxylanophilumgroup | 0.0537 | 0.00279988 | 26.74374056 |
| 7 | 43566344 | rs13239072 | A | G | 0.068742734 | 0.014272832 | 1.82E-06 | 13307 | Eubacteriumxylanophilumgroup | 0.2753 | 0.00174019 | 23.19359371 |
| 17 | 66689958 | rs79582700 | G | C | -0.095167651 | 0.019996897 | 2.41E-06 | 13256 | Eubacteriumxylanophilumgroup | 0.1074 | 0.001705688 | 22.64581571 |
| 19 | 8255478 | rs12980122 | G | C | -0.108273525 | 0.023825715 | 5.02E-06 | 12039 | Eubacteriumxylanophilumgroup | 0.0845 | 0.00171245 | 20.64811989 |
| 16 | 84790559 | rs2012708 | G | A | 0.057320248 | 0.012690502 | 6.53E-06 | 13306 | Eubacteriumxylanophilumgroup | 0.3638 | 0.001530896 | 20.39826484 |
| 11 | 131009660 | rs2213117 | G | T | 0.087779736 | 0.018901228 | 4.21E-06 | 12878 | Eubacteriumxylanophilumgroup | 0.161 | 0.001671989 | 21.56458123 |
| 14 | 73181783 | rs10140184 | C | A | 0.057679453 | 0.01259899 | 4.96E-06 | 12930 | Eubacteriumxylanophilumgroup | 0.4125 | 0.001618337 | 20.95577018 |
| 1 | 22627266 | rs10917203 | C | A | 0.061263327 | 0.013091688 | 3.15E-06 | 12930 | Eubacteriumxylanophilumgroup | 0.335 | 0.001690738 | 21.89488392 |
| 16 | 73696771 | rs112176119 | T | C | -0.113455468 | 0.024576196 | 3.33E-06 | 12199 | Eubacteriumxylanophilumgroup | 0.0646 | 0.001743971 | 21.30837582 |
| 9 | 130498120 | rs1999224 | T | G | -0.094905143 | 0.020350281 | 3.75E-06 | 13309 | Eubacteriumxylanophilumgroup | 0.0885 | 0.001631489 | 21.74569965 |
| 4 | 87891938 | rs75586835 | G | A | -0.114497555 | 0.026345472 | 9.39E-06 | 12550 | Eubacteriumxylanophilumgroup | 0.0666 | 0.001502739 | 18.88474978 |
| 1 | 246780557 | rs4654122 | G | C | 0.055350808 | 0.012358794 | 7.20E-06 | 13309 | Eubacteriumxylanophilumgroup | 0.4394 | 0.001504861 | 20.05535986 |
| 12 | 83502773 | rs12320842 | G | C | 0.094832958 | 0.016396224 | 7.57E-09 | 17638 | Faecalibacterium | 0.1322 | 0.001893035 | 33.44888594 |
| 6 | 131587157 | rs6910935 | G | A | 0.134863869 | 0.027703163 | 1.38E-06 | 12640 | Faecalibacterium | 0.0537 | 0.001871423 | 23.69538639 |
| 14 | 64044097 | rs1271565 | T | C | -0.057625117 | 0.011964928 | 1.30E-06 | 17638 | Faecalibacterium | 0.2724 | 0.001313359 | 23.19285511 |
| 16 | 6099863 | rs75499067 | T | C | 0.227584711 | 0.046552213 | 1.76E-06 | 4526 | Faecalibacterium | 0.0636 | 0.005252954 | 23.88985684 |
| 13 | 53567017 | rs9536330 | C | T | -0.048317039 | 0.01079702 | 5.33E-06 | 18082 | Faecalibacterium | 0.4085 | 0.001106282 | 20.02372188 |
| 15 | 47404021 | rs28376661 | G | C | 0.05048173 | 0.010936797 | 3.66E-06 | 18087 | Faecalibacterium | 0.3738 | 0.00117655 | 21.30296884 |
| 1 | 245267339 | rs10927394 | T | G | -0.232262107 | 0.051236432 | 7.02E-06 | 3495 | Faecalibacterium | 0.0268 | 0.005845286 | 20.5376321 |
| 2 | 37125235 | rs114946999 | T | C | -0.086156499 | 0.018960043 | 5.70E-06 | 15673 | Faecalibacterium | 0.0984 | 0.00131575 | 20.64629103 |
| 1 | 111036827 | rs79656633 | C | T | 0.145630704 | 0.032301873 | 8.14E-06 | 9080 | Faecalibacterium | 0.1054 | 0.002233539 | 20.32145542 |
| 10 | 128614990 | rs61875484 | G | C | 0.081795263 | 0.018369023 | 9.18E-06 | 16718 | Faecalibacterium | 0.0905 | 0.001184636 | 19.82586973 |
| 1 | 14201546 | rs12753492 | C | A | 0.064130474 | 0.014993333 | 8.80E-06 | 17525 | Faecalibacterium | 0.1491 | 0.001042849 | 18.29291646 |
| 21 | 39027901 | rs2835874 | C | T | -0.086635636 | 0.019649581 | 7.54E-06 | 17567 | Faecalibacterium | 0.0696 | 0.001105372 | 19.43735284 |
| 8 | 38593645 | rs11776390 | C | T | -0.078353971 | 0.017183046 | 6.40E-06 | 16718 | Faecalibacterium | 0.0895 | 0.001242217 | 20.79072998 |
| 3 | 3556826 | rs9852893 | G | C | 0.065783419 | 0.012905622 | 3.88E-07 | 14184 | FamilyXIIIAD3011group | 0.2565 | 0.001828443 | 25.97848286 |
| 20 | 4618639 | rs62200412 | T | C | -0.080085132 | 0.016383437 | 5.80E-07 | 13661 | FamilyXIIIAD3011group | 0.171 | 0.001746033 | 23.89077237 |
| 1 | 240694163 | rs16840310 | G | A | -0.060805195 | 0.012214901 | 6.75E-07 | 14201 | FamilyXIIIAD3011group | 0.3718 | 0.001741909 | 24.77652037 |
| 1 | 192019835 | rs72730932 | A | C | -0.089955866 | 0.017710652 | 6.89E-07 | 13102 | FamilyXIIIAD3011group | 0.164 | 0.00196516 | 25.79428983 |
| 15 | 58683697 | rs16940167 | T | C | 0.073255157 | 0.015989797 | 3.91E-06 | 14201 | FamilyXIIIAD3011group | 0.169 | 0.001475809 | 20.98598514 |
| 7 | 28603985 | rs17156849 | A | G | -0.112890921 | 0.024529236 | 4.19E-06 | 13352 | FamilyXIIIAD3011group | 0.0626 | 0.001583854 | 21.17799965 |
| 16 | 16006242 | rs62029761 | G | A | 0.128752725 | 0.027600154 | 3.89E-06 | 9655 | FamilyXIIIAD3011group | 0.0676 | 0.002248846 | 21.75704341 |
| 4 | 38917830 | rs11736617 | A | G | -0.075949337 | 0.017210081 | 9.02E-06 | 13778 | FamilyXIIIAD3011group | 0.1203 | 0.001411505 | 19.47238218 |
| 12 | 18127597 | rs12812672 | C | T | -0.096065015 | 0.020828023 | 2.56E-06 | 13565 | FamilyXIIIAD3011group | 0.0736 | 0.001565792 | 21.27014223 |
| 6 | 32695336 | rs9276029 | G | A | -0.081138392 | 0.018566861 | 8.93E-06 | 13566 | FamilyXIIIAD3011group | 0.168 | 0.001405766 | 19.09465296 |
| 15 | 40047706 | rs12911842 | T | A | -0.081197635 | 0.018333544 | 6.91E-06 | 14125 | FamilyXIIIAD3011group | 0.1243 | 0.001386765 | 19.61247579 |
| 5 | 14159925 | rs149302 | C | T | -0.064563538 | 0.014322495 | 7.48E-06 | 14192 | FamilyXIIIAD3011group | 0.2127 | 0.00142979 | 20.31777189 |
| 3 | 29811865 | rs9837139 | G | A | 0.10752022 | 0.024048177 | 8.71E-06 | 13527 | FamilyXIIIAD3011group | 0.0815 | 0.001475615 | 19.98718997 |
| 9 | 136678954 | rs739451 | T | C | 0.064958894 | 0.014753414 | 7.88E-06 | 14197 | FamilyXIIIAD3011group | 0.1759 | 0.00136365 | 19.38344615 |
| 2 | 74195551 | rs11126423 | T | C | 0.090439883 | 0.019630134 | 5.91E-06 | 13735 | FamilyXIIIAD3011group | 0.1133 | 0.001543029 | 21.22316883 |
| 3 | 188224096 | rs1426266 | C | T | -0.066552114 | 0.013715073 | 1.25E-06 | 12751 | FamilyXIIIUCG001 | 0.2962 | 0.00184324 | 23.54286715 |
| 1 | 85228325 | rs12049454 | C | T | -0.064729775 | 0.013406537 | 1.17E-06 | 12748 | FamilyXIIIUCG001 | 0.3012 | 0.001825322 | 23.30809704 |
| 1 | 183503664 | rs3842897 | A | G | -0.112639326 | 0.024275096 | 5.20E-06 | 12407 | FamilyXIIIUCG001 | 0.0646 | 0.001732361 | 21.5272313 |
| 15 | 25151194 | rs116979587 | A | T | -0.121678937 | 0.02608207 | 3.05E-06 | 11989 | FamilyXIIIUCG001 | 0.0706 | 0.001812074 | 21.76076875 |
| 6 | 76598635 | rs62414802 | T | C | -0.061190403 | 0.013456987 | 4.29E-06 | 12750 | FamilyXIIIUCG001 | 0.2982 | 0.001619038 | 20.67296773 |
| 21 | 45153079 | rs2276529 | G | C | -0.075940605 | 0.016509926 | 5.36E-06 | 12748 | FamilyXIIIUCG001 | 0.1859 | 0.001656897 | 21.15386277 |
| 17 | 33761027 | rs112362903 | G | A | -0.149047048 | 0.033327141 | 7.88E-06 | 8579 | FamilyXIIIUCG001 | 0.0427 | 0.002325962 | 19.99628812 |
| 17 | 78223817 | rs8076666 | G | A | 0.088655005 | 0.019809411 | 8.02E-06 | 12265 | FamilyXIIIUCG001 | 0.1362 | 0.001630374 | 20.02592515 |
| 11 | 94681786 | rs7119679 | A | G | -0.080908465 | 0.017478204 | 3.52E-06 | 12445 | FamilyXIIIUCG001 | 0.1501 | 0.001718907 | 21.42518228 |
| 3 | 45479090 | rs76463770 | G | A | 0.193129687 | 0.04198659 | 3.77E-06 | 5651 | FamilyXIIIUCG001 | 0.0517 | 0.003730169 | 21.15062128 |
| 1 | 77887835 | rs12030302 | G | A | -0.069227582 | 0.013746695 | 5.61E-07 | 10805 | Flavonifractor | 0.4911 | 0.002341633 | 25.35603535 |
| 10 | 32381186 | rs806808 | C | T | 0.0667272 | 0.013655748 | 1.18E-06 | 10801 | Flavonifractor | 0.4761 | 0.002205727 | 23.87230626 |
| 11 | 44849689 | rs34066017 | G | A | 0.076429405 | 0.015978927 | 1.52E-06 | 10705 | Flavonifractor | 0.2455 | 0.002132612 | 22.87412946 |
| 1 | 237351354 | rs11811696 | C | T | -0.116062715 | 0.024101172 | 2.07E-06 | 10221 | Flavonifractor | 0.1103 | 0.002263766 | 23.18590913 |
| 16 | 6696929 | rs11642826 | C | G | 0.146860461 | 0.032519094 | 6.65E-06 | 8151 | Flavonifractor | 0.0636 | 0.002495955 | 20.39042752 |
| 5 | 168218403 | rs114873521 | T | C | -0.130066614 | 0.029408684 | 7.13E-06 | 9156 | Flavonifractor | 0.0765 | 0.002131807 | 19.55625147 |
| 2 | 50201547 | rs6761463 | C | G | -0.083391953 | 0.018459615 | 8.11E-06 | 10805 | Flavonifractor | 0.165 | 0.001885206 | 20.40434225 |
| 1 | 102287083 | rs12038887 | G | C | 0.094271903 | 0.021164606 | 9.37E-06 | 10583 | Flavonifractor | 0.1093 | 0.001871208 | 19.83636456 |
| 1 | 24927625 | rs4378146 | C | A | -0.061669056 | 0.012525884 | 7.20E-07 | 17384 | Fusicatenibacter | 0.2416 | 0.001392397 | 24.23638524 |
| 5 | 29595000 | rs62353480 | G | A | -0.070139694 | 0.014558884 | 1.57E-06 | 16119 | Fusicatenibacter | 0.171 | 0.001437833 | 23.20693012 |
| 3 | 64252803 | rs704418 | C | T | 0.073912422 | 0.015107283 | 7.77E-07 | 16626 | Fusicatenibacter | 0.1859 | 0.001437638 | 23.93370114 |
| 8 | 15360718 | rs2132128 | A | G | -0.07719139 | 0.016034448 | 1.08E-06 | 17370 | Fusicatenibacter | 0.165 | 0.00133245 | 23.1728632 |
| 18 | 10370596 | rs206581 | G | A | -0.056833856 | 0.012789397 | 8.96E-06 | 17384 | Fusicatenibacter | 0.2087 | 0.001134675 | 19.7453183 |
| 2 | 226649570 | rs62187631 | C | T | -0.07105711 | 0.015924014 | 4.55E-06 | 16522 | Fusicatenibacter | 0.1402 | 0.001203717 | 19.90936562 |
| 10 | 112177119 | rs2025938 | A | G | -0.096729791 | 0.020542557 | 2.99E-06 | 15626 | Fusicatenibacter | 0.0765 | 0.001416928 | 22.16949964 |
| 10 | 120447429 | rs3303 | C | T | -0.095365713 | 0.020406992 | 3.94E-06 | 16877 | Fusicatenibacter | 0.0765 | 0.001292319 | 21.83610042 |
| 9 | 130371107 | rs2039204 | A | T | -0.04974145 | 0.010794137 | 3.94E-06 | 17383 | Fusicatenibacter | 0.4314 | 0.00122013 | 21.2329871 |
| 15 | 88353215 | rs8028026 | G | A | -0.079214582 | 0.018050473 | 8.06E-06 | 16522 | Fusicatenibacter | 0.0915 | 0.001164299 | 19.2566408 |
| 17 | 70721782 | rs1864685 | C | A | -0.049479928 | 0.010810494 | 4.96E-06 | 17374 | Fusicatenibacter | 0.4652 | 0.001204326 | 20.94676932 |
| 2 | 5532793 | rs792108 | C | T | -0.050834518 | 0.011384326 | 8.50E-06 | 16978 | Fusicatenibacter | 0.3539 | 0.001173023 | 19.93662225 |
| 7 | 148856720 | rs60254196 | G | A | -0.049242701 | 0.010936491 | 5.47E-06 | 16978 | Fusicatenibacter | 0.5408 | 0.001192677 | 20.27106702 |
| 17 | 70697344 | rs9905659 | A | G | -0.06161703 | 0.013657585 | 7.31E-06 | 17384 | Fusicatenibacter | 0.1879 | 0.001169488 | 20.35184277 |
| 16 | 73821805 | rs8063430 | C | T | -0.104026455 | 0.022216952 | 4.93E-06 | 15552 | Fusicatenibacter | 0.0596 | 0.001407734 | 21.92112332 |
| 20 | 25257001 | rs6515626 | A | G | 0.141575028 | 0.031355872 | 7.29E-06 | 7796 | Fusicatenibacter | 0.0616 | 0.002608132 | 20.38093531 |
| 21 | 42681392 | rs10439674 | G | A | -0.057212907 | 0.013000675 | 7.68E-06 | 17381 | Fusicatenibacter | 0.2018 | 0.001113007 | 19.36449734 |
| 20 | 55937366 | rs167879 | T | C | -0.065954324 | 0.014876261 | 5.87E-06 | 16542 | Fusicatenibacter | 0.167 | 0.001186848 | 19.65379767 |
| 12 | 58658311 | rs73103914 | G | A | -0.059732424 | 0.013445773 | 8.30E-06 | 17384 | Fusicatenibacter | 0.1779 | 0.001133982 | 19.73325607 |
| 3 | 187450354 | rs76287110 | T | A | -0.242964921 | 0.046617731 | 1.67E-07 | 3483 | Gordonibacter | 0.0706 | 0.007738514 | 27.14785001 |
| 12 | 29024534 | rs7294633 | T | C | 0.128670034 | 0.024994637 | 3.44E-07 | 3723 | Gordonibacter | 0.3509 | 0.007067856 | 26.48669737 |
| 17 | 10148653 | rs7220558 | T | A | 0.116884144 | 0.02348337 | 6.71E-07 | 3723 | Gordonibacter | 0.4324 | 0.006610236 | 24.76036152 |
| 7 | 47144505 | rs71545975 | G | A | -0.153972004 | 0.033891756 | 7.04E-06 | 3556 | Gordonibacter | 0.173 | 0.00577059 | 20.62771112 |
| 4 | 8142607 | rs35042269 | A | C | -0.180292677 | 0.040329634 | 8.11E-06 | 3595 | Gordonibacter | 0.1889 | 0.005528421 | 19.97404111 |
| 4 | 136768501 | rs72714787 | A | C | 0.181404779 | 0.037710172 | 1.43E-06 | 3723 | Gordonibacter | 0.1044 | 0.006177266 | 23.12847869 |
| 7 | 136937189 | rs322296 | A | G | 0.178689528 | 0.037721957 | 4.02E-06 | 3595 | Gordonibacter | 0.1193 | 0.006203096 | 22.42684014 |
| 1 | 83136096 | rs72939513 | G | A | -0.213989639 | 0.049059194 | 7.98E-06 | 3435 | Gordonibacter | 0.0537 | 0.005508319 | 19.01479893 |
| 1 | 210628449 | rs3765837 | G | T | -0.190728517 | 0.043360843 | 7.17E-06 | 3556 | Gordonibacter | 0.0785 | 0.005411498 | 19.33710711 |
| 2 | 30158494 | rs13412653 | C | A | 0.107597447 | 0.023922776 | 8.61E-06 | 3723 | Gordonibacter | 0.3539 | 0.005404237 | 20.2184299 |
| 7 | 129433205 | rs117347059 | C | G | -0.128261008 | 0.028512264 | 9.17E-06 | 3684 | Gordonibacter | 0.2068 | 0.00546295 | 20.22507063 |
| 17 | 52957210 | rs16955299 | A | G | -0.196422874 | 0.043353771 | 6.37E-06 | 3572 | Gordonibacter | 0.0835 | 0.005713869 | 20.51573708 |
| 9 | 88787144 | rs4596722 | G | A | 0.102908274 | 0.023157103 | 9.06E-06 | 3723 | Gordonibacter | 0.4761 | 0.005276446 | 19.73780101 |
| 7 | 18970607 | rs768830 | A | G | 0.149852407 | 0.033331454 | 7.76E-06 | 3723 | Gordonibacter | 0.1292 | 0.00539976 | 20.20159001 |
| 12 | 131024079 | rs61934597 | T | C | -0.172494533 | 0.038856681 | 8.37E-06 | 3684 | Gordonibacter | 0.0755 | 0.00532087 | 19.6962442 |
| 6 | 129224488 | rs12191680 | G | C | 0.106544332 | 0.020032321 | 1.47E-07 | 9115 | Haemophilus | 0.165 | 0.003093825 | 28.28152617 |
| 6 | 55448491 | rs9382510 | T | C | -0.093521046 | 0.017264852 | 7.12E-08 | 9117 | Haemophilus | 0.2455 | 0.003208083 | 29.33578419 |
| 13 | 36104947 | rs9574096 | T | A | -0.073637529 | 0.015522726 | 2.18E-06 | 9118 | Haemophilus | 0.3618 | 0.002462024 | 22.49920309 |
| 10 | 94306385 | rs76022354 | T | C | 0.244637568 | 0.050550437 | 1.83E-06 | 3618 | Haemophilus | 0.0328 | 0.006431698 | 23.4075704 |
| 16 | 48742489 | rs111582866 | A | G | -0.124265408 | 0.026016016 | 1.27E-06 | 8370 | Haemophilus | 0.1014 | 0.002718387 | 22.80946745 |
| 17 | 64535013 | rs9895850 | C | T | -0.192956876 | 0.041675519 | 2.14E-06 | 4592 | Haemophilus | 0.0497 | 0.004646585 | 21.42739112 |
| 12 | 115493337 | rs35509 | A | G | 0.128248955 | 0.026877911 | 2.01E-06 | 8430 | Haemophilus | 0.0785 | 0.002693504 | 22.76216572 |
| 9 | 105650242 | rs78909003 | C | T | -0.246255715 | 0.050392389 | 1.67E-06 | 3559 | Haemophilus | 0.0497 | 0.006665158 | 23.86704312 |
| 6 | 8350917 | rs9328464 | C | T | 0.072309489 | 0.014879949 | 1.42E-06 | 9119 | Haemophilus | 0.492 | 0.002582957 | 23.60980609 |
| 22 | 26891808 | rs4822728 | C | T | 0.070586099 | 0.015138761 | 3.48E-06 | 9119 | Haemophilus | 0.4374 | 0.002378353 | 21.73513896 |
| 11 | 9951672 | rs10840326 | G | C | -0.067738403 | 0.015146258 | 7.37E-06 | 8888 | Haemophilus | 0.4433 | 0.002245323 | 19.99684316 |
| 13 | 85114550 | rs12876183 | A | T | 0.074920105 | 0.016710973 | 9.62E-06 | 9110 | Haemophilus | 0.2753 | 0.002201495 | 20.0954586 |
| 9 | 78752170 | rs10781340 | A | G | 0.094891501 | 0.020322072 | 4.32E-06 | 9037 | Haemophilus | 0.166 | 0.002406843 | 21.79829494 |
| 7 | 24360361 | rs56310940 | C | G | -0.108393321 | 0.024705186 | 7.23E-06 | 9119 | Haemophilus | 0.0825 | 0.002106521 | 19.24569139 |
| 6 | 163603171 | rs761624 | G | C | 0.095980128 | 0.017953732 | 1.38E-07 | 7706 | Holdemanella | 0.3082 | 0.003695017 | 28.57198744 |
| 6 | 4148609 | rs607782 | C | T | -0.08542142 | 0.017251511 | 7.19E-07 | 7456 | Holdemanella | 0.3817 | 0.003277537 | 24.51110003 |
| 10 | 4494630 | rs75764681 | C | T | -0.283102607 | 0.059899873 | 1.94E-06 | 3003 | Holdemanella | 0.0626 | 0.007383497 | 22.32269349 |
| 19 | 15020095 | rs73011279 | C | T | -0.096166927 | 0.019933645 | 1.36E-06 | 7456 | Holdemanella | 0.2078 | 0.003111849 | 23.26813166 |
| 9 | 105004835 | rs4541991 | C | T | -0.092733374 | 0.019443459 | 2.10E-06 | 7657 | Holdemanella | 0.2356 | 0.002961953 | 22.74110761 |
| 7 | 14728950 | rs1830029 | G | C | -0.095409594 | 0.021093039 | 5.35E-06 | 7456 | Holdemanella | 0.1799 | 0.00273659 | 20.45451865 |
| 4 | 71000791 | rs12513188 | A | G | 0.090390629 | 0.019527621 | 4.65E-06 | 7700 | Holdemanella | 0.2217 | 0.002774921 | 21.42078271 |
| 10 | 125021315 | rs12415649 | C | G | 0.083938435 | 0.019056694 | 7.88E-06 | 7662 | Holdemanella | 0.2376 | 0.002525726 | 19.39604687 |
| 1 | 65153340 | rs1926302 | A | G | -0.107971755 | 0.023142056 | 7.50E-06 | 7702 | Holdemanella | 0.1531 | 0.0028183 | 21.76224235 |
| 13 | 50932063 | rs17586763 | C | T | -0.227284509 | 0.051013457 | 7.72E-06 | 3817 | Holdemanella | 0.0179 | 0.005173629 | 19.84004012 |
| 19 | 43851935 | rs8113760 | A | G | 0.078998984 | 0.017340046 | 4.62E-06 | 7456 | Holdemanella | 0.3519 | 0.002776063 | 20.75037789 |
| 8 | 138623633 | rs34187114 | A | C | -0.104505235 | 0.022600956 | 5.13E-06 | 7704 | Holdemanella | 0.166 | 0.002767595 | 21.3751762 |
| 3 | 85392882 | rs35228298 | A | G | 0.093483215 | 0.020288588 | 7.30E-06 | 7456 | Holdemanella | 0.2465 | 0.002839376 | 21.22497228 |
| 19 | 7668225 | rs62113381 | C | T | -0.105447171 | 0.023202912 | 5.54E-06 | 7310 | Holdemanella | 0.159 | 0.002817359 | 20.64743245 |
| 11 | 18768068 | rs1867876 | C | T | 0.084291862 | 0.016219181 | 2.74E-07 | 9109 | Holdemania | 0.2942 | 0.002956357 | 27.00337628 |
| 6 | 105737322 | rs9500080 | T | C | 0.092676436 | 0.01788862 | 4.09E-07 | 9106 | Holdemania | 0.2346 | 0.002938861 | 26.83425623 |
| 20 | 3839754 | rs6133067 | C | T | 0.091082873 | 0.017856087 | 5.17E-07 | 8863 | Holdemania | 0.3111 | 0.002927164 | 26.01374354 |
| 12 | 39589195 | rs150096134 | A | T | 0.162139279 | 0.033208945 | 2.38E-06 | 6906 | Holdemania | 0.0626 | 0.00343988 | 23.83090511 |
| 14 | 102553414 | rs41438744 | G | C | -0.124870498 | 0.02701295 | 2.44E-06 | 8839 | Holdemania | 0.0726 | 0.002411703 | 21.36374481 |
| 5 | 77388406 | rs77293403 | G | A | 0.164556483 | 0.034177476 | 1.77E-06 | 7585 | Holdemania | 0.0457 | 0.003046977 | 23.17584055 |
| 10 | 129908282 | rs80149660 | T | C | -0.23298738 | 0.051918311 | 6.04E-06 | 3283 | Holdemania | 0.0586 | 0.006096729 | 20.12607259 |
| 13 | 81685543 | rs116500994 | T | G | -0.137550456 | 0.029338596 | 2.34E-06 | 8432 | Holdemania | 0.0517 | 0.002600065 | 21.97568391 |
| 17 | 76443497 | rs55888180 | G | C | 0.128670991 | 0.028279464 | 5.89E-06 | 8464 | Holdemania | 0.0875 | 0.002439958 | 20.69742396 |
| 5 | 106209093 | rs4146507 | T | C | 0.079488043 | 0.017700231 | 7.23E-06 | 9108 | Holdemania | 0.2217 | 0.002209338 | 20.16277842 |
| 15 | 93110222 | rs111745969 | G | A | 0.120676888 | 0.026578103 | 3.71E-06 | 8233 | Holdemania | 0.0795 | 0.00249779 | 20.61079483 |
| 8 | 123973840 | rs113593397 | G | A | -0.128934656 | 0.028252607 | 9.36E-06 | 8296 | Holdemania | 0.1024 | 0.002504175 | 20.82176588 |
| 10 | 115332413 | rs10885477 | C | T | -0.13513664 | 0.030189444 | 8.60E-06 | 8690 | Holdemania | 0.0616 | 0.002300467 | 20.03254141 |
| 13 | 70856516 | rs9529719 | C | T | 0.074037972 | 0.016049433 | 5.97E-06 | 9107 | Holdemania | 0.325 | 0.002331313 | 21.27621036 |
| 3 | 60615680 | rs967319 | C | T | 0.078863839 | 0.017674402 | 8.38E-06 | 9108 | Holdemania | 0.2465 | 0.002181198 | 19.90540466 |
| 7 | 38292857 | rs12701617 | G | A | -0.066061737 | 0.014942681 | 9.52E-06 | 9106 | Holdemania | 0.4583 | 0.002141825 | 19.5410327 |
| 7 | 63323145 | rs73139538 | A | G | -0.148593595 | 0.032746911 | 7.77E-06 | 8095 | Holdemania | 0.0527 | 0.002537111 | 20.58506256 |
| 17 | 26789271 | rs11080063 | A | G | -0.066523819 | 0.014983728 | 6.67E-06 | 9103 | Holdemania | 0.4662 | 0.002160681 | 19.70693927 |
| 11 | 77901287 | rs901099 | G | T | -0.127180025 | 0.025092403 | 6.53E-07 | 3647 | Howardella | 0.3121 | 0.0069947 | 25.67527284 |
| 18 | 43206985 | rs1484873 | G | A | -0.227831171 | 0.046335026 | 2.56E-06 | 3608 | Howardella | 0.0686 | 0.006656415 | 24.16387886 |
| 7 | 133154356 | rs17167098 | A | G | -0.16936566 | 0.035206781 | 1.12E-06 | 3586 | Howardella | 0.1133 | 0.006412009 | 23.12894371 |
| 4 | 169178315 | rs609430 | G | T | -0.112045365 | 0.023932786 | 3.34E-06 | 3798 | Howardella | 0.339 | 0.005737824 | 21.90647592 |
| 7 | 93527414 | rs36081916 | C | T | -0.181229725 | 0.040298718 | 4.70E-06 | 3216 | Howardella | 0.1113 | 0.006249392 | 20.21185763 |
| 17 | 17253288 | rs12452946 | G | A | -0.105833103 | 0.022894084 | 3.80E-06 | 3800 | Howardella | 0.501 | 0.005592133 | 21.35835941 |
| 1 | 72972799 | rs61771805 | T | A | -0.136778682 | 0.029681112 | 4.03E-06 | 3612 | Howardella | 0.1869 | 0.005844978 | 21.22442644 |
| 18 | 60125134 | rs672217 | A | G | 0.164145673 | 0.034999266 | 3.52E-06 | 3611 | Howardella | 0.1412 | 0.006054469 | 21.98367978 |
| 2 | 218819396 | rs3791893 | G | A | 0.147035334 | 0.034023014 | 9.50E-06 | 3795 | Howardella | 0.1233 | 0.004897269 | 18.66675846 |
| 15 | 98476920 | rs10048062 | T | C | -0.14735223 | 0.033653121 | 8.59E-06 | 3800 | Howardella | 0.1074 | 0.005019882 | 19.16170284 |
| 14 | 95452797 | rs2154047 | A | C | -0.192557008 | 0.042009328 | 9.97E-06 | 3623 | Howardella | 0.0676 | 0.005765641 | 20.9984566 |
| 4 | 166058998 | rs13128780 | C | T | -0.149725257 | 0.031277743 | 1.75E-06 | 3999 | Hungatella | 0.1451 | 0.005697528 | 22.90351165 |
| 15 | 89577376 | rs72759041 | T | G | -0.126025023 | 0.028224238 | 3.86E-06 | 3910 | Hungatella | 0.2346 | 0.005073218 | 19.9272293 |
| 5 | 58424440 | rs10044993 | A | C | 0.139546965 | 0.031674777 | 8.07E-06 | 4209 | Hungatella | 0.1173 | 0.004590253 | 19.40024578 |
| 8 | 14853525 | rs13249325 | G | T | -0.100022583 | 0.022588331 | 9.69E-06 | 4209 | Hungatella | 0.3668 | 0.004636929 | 19.59843579 |
| 14 | 95973619 | rs17092615 | A | G | 0.152234676 | 0.033786729 | 7.38E-06 | 4009 | Hungatella | 0.1243 | 0.005038544 | 20.29168769 |
| 4 | 14324623 | rs10805326 | A | G | 0.077515061 | 0.013966468 | 3.55E-08 | 12303 | Intestinibacter | 0.2783 | 0.00249748 | 30.79841958 |
| 15 | 92446683 | rs4327025 | A | G | -0.081034559 | 0.015439666 | 1.64E-07 | 12303 | Intestinibacter | 0.1948 | 0.002233996 | 27.541915 |
| 11 | 125663184 | rs478972 | C | T | -0.142680973 | 0.02971163 | 1.82E-06 | 10333 | Intestinibacter | 0.0596 | 0.002226817 | 23.05659183 |
| 5 | 159686256 | rs6875660 | T | C | 0.089045542 | 0.01939052 | 3.06E-06 | 12293 | Intestinibacter | 0.1233 | 0.00171255 | 21.08505666 |
| 9 | 21502923 | rs16938435 | C | T | -0.112188506 | 0.023543887 | 1.80E-06 | 12303 | Intestinibacter | 0.0606 | 0.001842165 | 22.70229637 |
| 20 | 61325223 | rs6062862 | G | A | 0.092450812 | 0.020465817 | 6.68E-06 | 12189 | Intestinibacter | 0.0944 | 0.001671355 | 20.40290464 |
| 16 | 5993076 | rs118030283 | A | G | -0.15182897 | 0.032447117 | 2.67E-06 | 8830 | Intestinibacter | 0.0378 | 0.002473551 | 21.89065167 |
| 12 | 97928437 | rs11109097 | T | C | 0.062427238 | 0.013853923 | 5.49E-06 | 11886 | Intestinibacter | 0.3121 | 0.001705398 | 20.30156907 |
| 6 | 10303945 | rs9348442 | T | C | 0.099081964 | 0.022162526 | 6.26E-06 | 12303 | Intestinibacter | 0.0875 | 0.00162194 | 19.98389246 |
| 5 | 148846426 | rs447950 | G | A | 0.062834174 | 0.01366523 | 5.64E-06 | 12303 | Intestinibacter | 0.2992 | 0.001715541 | 21.13913916 |
| 6 | 170918194 | rs62430350 | C | T | 0.151095721 | 0.035147611 | 6.84E-06 | 7650 | Intestinibacter | 0.0577 | 0.002409924 | 18.47562525 |
| 4 | 179361262 | rs2702387 | G | A | 0.060855918 | 0.013214549 | 4.26E-06 | 12291 | Intestinibacter | 0.3519 | 0.001722523 | 21.20461034 |
| 3 | 70657785 | rs68093214 | T | C | 0.066225756 | 0.014987729 | 9.26E-06 | 11886 | Intestinibacter | 0.2575 | 0.001639961 | 19.52131563 |
| 11 | 127703160 | rs2098844 | T | C | -0.057529528 | 0.012841562 | 6.79E-06 | 12291 | Intestinibacter | 0.4245 | 0.001630235 | 20.06666726 |
| 2 | 20054971 | rs893394 | A | G | 0.058332172 | 0.013072938 | 7.85E-06 | 11886 | Intestinibacter | 0.4195 | 0.001672274 | 19.90658874 |
| 10 | 13124513 | rs11258178 | G | A | 0.066074232 | 0.013413886 | 6.98E-07 | 11766 | Intestinimonas | 0.4145 | 0.002057934 | 24.25946426 |
| 11 | 94364293 | rs12226153 | G | A | -0.151142451 | 0.030692229 | 5.12E-07 | 9023 | Intestinimonas | 0.0616 | 0.002680399 | 24.24486611 |
| 16 | 58818770 | rs4784055 | C | T | -0.175323954 | 0.038599023 | 8.72E-07 | 3193 | Intestinimonas | 0.0557 | 0.006419982 | 20.61853385 |
| 2 | 6514088 | rs716604 | G | A | 0.081806835 | 0.016599238 | 8.57E-07 | 12090 | Intestinimonas | 0.1879 | 0.002004956 | 24.28460333 |
| 16 | 85327902 | rs2930225 | T | G | 0.072952384 | 0.015294848 | 1.35E-06 | 12003 | Intestinimonas | 0.2584 | 0.001891805 | 22.74658991 |
| 3 | 10611142 | rs62240188 | A | G | 0.130073573 | 0.026717884 | 2.20E-06 | 10252 | Intestinimonas | 0.0686 | 0.002306548 | 23.69677773 |
| 5 | 17209391 | rs2731794 | T | C | 0.120632118 | 0.025752925 | 1.92E-06 | 11079 | Intestinimonas | 0.0596 | 0.001976573 | 21.93786534 |
| 15 | 93819735 | rs7170984 | C | T | -0.06581157 | 0.014076733 | 2.98E-06 | 12090 | Intestinimonas | 0.2903 | 0.001804638 | 21.85389847 |
| 7 | 66890843 | rs10262702 | C | T | 0.091801712 | 0.019488468 | 2.06E-06 | 11766 | Intestinimonas | 0.1292 | 0.001882344 | 22.18565899 |
| 1 | 43767569 | rs12566247 | A | T | 0.063708441 | 0.013535548 | 2.19E-06 | 12076 | Intestinimonas | 0.3479 | 0.001831147 | 22.14982698 |
| 7 | 22007303 | rs1859797 | A | G | 0.060367955 | 0.013179253 | 4.12E-06 | 11766 | Intestinimonas | 0.504 | 0.001780036 | 20.97768079 |
| 8 | 3763540 | rs17067892 | T | C | 0.107193792 | 0.025001344 | 6.38E-06 | 11544 | Intestinimonas | 0.0765 | 0.001589883 | 18.3796528 |
| 2 | 140898088 | rs72982915 | T | C | 0.183157909 | 0.040274272 | 4.91E-06 | 5591 | Intestinimonas | 0.0457 | 0.003685555 | 20.6747647 |
| 4 | 114608814 | rs994794 | C | G | -0.141904818 | 0.031507362 | 7.31E-06 | 9250 | Intestinimonas | 0.0398 | 0.00218815 | 20.28038928 |
| 20 | 6218922 | rs4113676 | C | A | -0.218506906 | 0.049015185 | 7.42E-06 | 3170 | Intestinimonas | 0.0616 | 0.006230108 | 19.86071802 |
| 3 | 17187502 | rs9823439 | C | T | -0.058194711 | 0.013145465 | 9.86E-06 | 11766 | Intestinimonas | 0.4414 | 0.00166289 | 19.59482032 |
| 6 | 155737906 | rs62427239 | A | C | 0.162658132 | 0.036764706 | 9.41E-06 | 6385 | Intestinimonas | 0.0457 | 0.003056322 | 19.56830911 |
| 3 | 150620956 | rs2276760 | G | A | -0.06852335 | 0.015254563 | 7.84E-06 | 12081 | Intestinimonas | 0.2197 | 0.001667439 | 20.17463753 |
| 6 | 67200802 | rs6934519 | T | C | 0.069237276 | 0.015115352 | 8.57E-06 | 12089 | Intestinimonas | 0.2753 | 0.001732606 | 20.97836171 |
| 13 | 23343542 | rs1000888 | G | C | -0.058503766 | 0.01321901 | 9.71E-06 | 12090 | Intestinimonas | 0.3956 | 0.001617486 | 19.58384226 |
| 20 | 19300846 | rs6112314 | C | A | -0.056171476 | 0.010817419 | 2.43E-07 | 17842 | Lachnoclostridium | 0.3966 | 0.001508985 | 26.96097924 |
| 3 | 177470032 | rs62285313 | G | A | 0.086420325 | 0.018156544 | 1.58E-06 | 16991 | Lachnoclostridium | 0.0855 | 0.001331582 | 22.65240347 |
| 17 | 13816159 | rs78068103 | G | A | 0.088619929 | 0.019424795 | 3.67E-06 | 17477 | Lachnoclostridium | 0.0915 | 0.001189505 | 20.8113488 |
| 3 | 23037786 | rs615997 | C | T | 0.05117524 | 0.010649056 | 2.03E-06 | 17911 | Lachnoclostridium | 0.499 | 0.00128771 | 23.09133607 |
| 12 | 28169306 | rs61915992 | T | A | 0.080387577 | 0.01722087 | 2.67E-06 | 16773 | Lachnoclostridium | 0.1233 | 0.001297457 | 21.78792792 |
| 18 | 1053252 | rs789029 | T | C | -0.06412885 | 0.013797406 | 3.75E-06 | 17922 | Lachnoclostridium | 0.1889 | 0.001203933 | 21.60048771 |
| 1 | 185091351 | rs12566975 | C | T | -0.04680969 | 0.010578675 | 9.57E-06 | 17921 | Lachnoclostridium | 0.493 | 0.001091371 | 19.57764863 |
| 8 | 59370320 | rs4738679 | A | G | -0.052026675 | 0.011404049 | 4.42E-06 | 17921 | Lachnoclostridium | 0.3648 | 0.001160026 | 20.81064516 |
| 3 | 66725825 | rs1031599 | T | G | -0.078627049 | 0.017564435 | 6.31E-06 | 17398 | Lachnoclostridium | 0.0845 | 0.001150473 | 20.03667599 |
| 17 | 46694541 | rs72829893 | T | G | 0.117472377 | 0.026810315 | 5.58E-06 | 12404 | Lachnoclostridium | 0.0656 | 0.001545376 | 19.19542156 |
| 4 | 38694566 | rs3821998 | A | C | -0.0864066 | 0.019251946 | 6.72E-06 | 17920 | Lachnoclostridium | 0.0885 | 0.001122842 | 20.14169913 |
| 18 | 19743455 | rs2385421 | G | A | 0.074618572 | 0.018073408 | 7.14E-06 | 17903 | Lachnoclostridium | 0.1044 | 0.000951206 | 17.04375269 |
| 2 | 167244051 | rs1528479 | A | G | -0.049779849 | 0.011191926 | 9.64E-06 | 17477 | Lachnoclostridium | 0.3698 | 0.001130679 | 19.78098015 |
| 12 | 102046595 | rs1997204 | C | T | -0.108074801 | 0.024202203 | 5.97E-06 | 14138 | Lachnoclostridium | 0.0527 | 0.001408442 | 19.93781894 |
| 16 | 27217620 | rs62028349 | C | G | 0.046998893 | 0.010597086 | 9.17E-06 | 17922 | Lachnoclostridium | 0.505 | 0.001096325 | 19.66771151 |
| 5 | 176802250 | rs13157098 | G | A | -0.076805837 | 0.015531203 | 5.99E-07 | 15947 | Lachnospira | 0.167 | 0.001531206 | 24.45252864 |
| 11 | 25962890 | rs4923324 | A | G | -0.061732096 | 0.013338657 | 2.44E-06 | 16490 | Lachnospira | 0.2386 | 0.001297219 | 21.41633618 |
| 2 | 57701660 | rs56791201 | C | T | 0.051822993 | 0.011069673 | 2.93E-06 | 16486 | Lachnospira | 0.4573 | 0.001327649 | 21.9140527 |
| 6 | 6893668 | rs2326833 | G | C | -0.078495035 | 0.017109015 | 4.60E-06 | 15632 | Lachnospira | 0.1252 | 0.001344731 | 21.04644577 |
| 3 | 186445436 | rs4686798 | C | T | 0.053182605 | 0.011374743 | 2.74E-06 | 16497 | Lachnospira | 0.3857 | 0.001323354 | 21.85764535 |
| 4 | 111995627 | rs159484 | A | G | 0.079466817 | 0.017699872 | 6.68E-06 | 16487 | Lachnospira | 0.1133 | 0.001221122 | 20.15480959 |
| 12 | 91006514 | rs2520509 | G | A | 0.051906257 | 0.011577959 | 7.42E-06 | 16500 | Lachnospira | 0.3181 | 0.001216642 | 20.09660691 |
| 19 | 2571232 | rs7249113 | A | G | 0.067948308 | 0.013349587 | 3.72E-07 | 13534 | LachnospiraceaeFCS020group | 0.2813 | 0.001910579 | 25.90343945 |
| 1 | 35328576 | rs12078956 | G | C | 0.106101007 | 0.022290421 | 2.15E-06 | 13101 | LachnospiraceaeFCS020group | 0.1213 | 0.001726425 | 22.65354478 |
| 16 | 23631307 | rs369444 | G | C | 0.125481334 | 0.025900786 | 3.15E-06 | 12465 | LachnospiraceaeFCS020group | 0.0636 | 0.001879417 | 23.46727504 |
| 2 | 53946431 | rs72793667 | G | A | -0.116880798 | 0.024658906 | 1.63E-06 | 12396 | LachnospiraceaeFCS020group | 0.0835 | 0.001809134 | 22.4630455 |
| 19 | 17865305 | rs1363769 | C | T | -0.200628437 | 0.044937217 | 1.58E-06 | 3555 | LachnospiraceaeFCS020group | 0.0328 | 0.005575769 | 19.92178479 |
| 8 | 121244406 | rs10093861 | A | G | -0.056886893 | 0.012115188 | 3.06E-06 | 13772 | LachnospiraceaeFCS020group | 0.498 | 0.001598352 | 22.04453997 |
| 13 | 40419314 | rs9788306 | T | C | -0.062803973 | 0.013074422 | 1.39E-06 | 13772 | LachnospiraceaeFCS020group | 0.2843 | 0.00167265 | 23.07098507 |
| 5 | 109403564 | rs113859143 | C | G | -0.108906331 | 0.024205472 | 2.55E-06 | 13238 | LachnospiraceaeFCS020group | 0.0845 | 0.001526839 | 20.24014028 |
| 1 | 118425334 | rs9919338 | C | G | -0.055333855 | 0.012062109 | 4.91E-06 | 13768 | LachnospiraceaeFCS020group | 0.4364 | 0.001526164 | 21.04128594 |
| 11 | 3248876 | rs35035870 | C | T | -0.190615272 | 0.041440269 | 2.62E-06 | 5642 | LachnospiraceaeFCS020group | 0.0358 | 0.003736041 | 21.1502872 |
| 3 | 165996415 | rs2862811 | C | T | 0.056492728 | 0.012173604 | 3.92E-06 | 13772 | LachnospiraceaeFCS020group | 0.4543 | 0.001561245 | 21.53196615 |
| 10 | 44346094 | rs1254846 | A | G | 0.105962631 | 0.023250118 | 5.60E-06 | 13172 | LachnospiraceaeFCS020group | 0.0915 | 0.001574415 | 20.76773758 |
| 6 | 71966268 | rs4452603 | G | T | 0.060420825 | 0.013596453 | 8.98E-06 | 13487 | LachnospiraceaeFCS020group | 0.2863 | 0.00146208 | 19.74502227 |
| 4 | 166588085 | rs2322265 | T | C | -0.0666305 | 0.014157654 | 5.21E-06 | 13772 | LachnospiraceaeFCS020group | 0.2555 | 0.001605716 | 22.14626389 |
| 10 | 65563647 | rs3999074 | T | G | -0.05505865 | 0.012184677 | 6.55E-06 | 13772 | LachnospiraceaeFCS020group | 0.4781 | 0.001480412 | 20.41549984 |
| 4 | 165786478 | rs9308097 | G | A | 0.05538101 | 0.012365944 | 7.47E-06 | 13542 | LachnospiraceaeFCS020group | 0.4523 | 0.00147891 | 20.0540938 |
| 20 | 5330700 | rs6116753 | A | G | 0.099474834 | 0.020913933 | 2.92E-06 | 6278 | LachnospiraceaeNC2004group | 0.2396 | 0.003590637 | 22.6160453 |
| 5 | 149544722 | rs3756315 | G | A | -0.088346946 | 0.01883968 | 3.33E-06 | 6328 | LachnospiraceaeNC2004group | 0.339 | 0.003463083 | 21.98359724 |
| 1 | 234157721 | rs12127733 | A | G | 0.115181538 | 0.024600625 | 3.11E-06 | 6226 | LachnospiraceaeNC2004group | 0.1779 | 0.003508633 | 21.91461975 |
| 18 | 58229086 | rs17067076 | A | G | -0.154631362 | 0.035219374 | 5.61E-06 | 4449 | LachnospiraceaeNC2004group | 0.0765 | 0.004314116 | 19.26799931 |
| 9 | 122239986 | rs1331592 | G | C | 0.094880626 | 0.02083688 | 5.34E-06 | 6328 | LachnospiraceaeNC2004group | 0.2286 | 0.003265898 | 20.72776281 |
| 9 | 29334137 | rs1928659 | C | T | 0.102522036 | 0.022644542 | 6.17E-06 | 5516 | LachnospiraceaeNC2004group | 0.2495 | 0.003702305 | 20.49037314 |
| 13 | 76829562 | rs1929743 | C | T | 0.083720631 | 0.019031847 | 9.06E-06 | 6330 | LachnospiraceaeNC2004group | 0.3121 | 0.003047711 | 19.34487262 |
| 13 | 47512191 | rs12863463 | A | G | -0.156364942 | 0.03453716 | 6.04E-06 | 5516 | LachnospiraceaeNC2004group | 0.0547 | 0.00370229 | 20.49028662 |
| 17 | 21157406 | rs117467633 | C | T | -0.169687489 | 0.038316857 | 9.13E-06 | 5077 | LachnospiraceaeNC2004group | 0.0626 | 0.003848029 | 19.60418464 |
| 6 | 22725908 | rs12208226 | A | C | -0.154739126 | 0.034037233 | 9.75E-06 | 5620 | LachnospiraceaeNC2004group | 0.1064 | 0.003664049 | 20.66032713 |
| 16 | 1100633 | rs9932954 | G | A | -0.056186355 | 0.011598415 | 1.25E-06 | 15222 | LachnospiraceaeND3007group | 0.4284 | 0.001539302 | 23.46428806 |
| 4 | 7678735 | rs13110238 | G | C | -0.063697828 | 0.01420413 | 4.48E-06 | 15019 | LachnospiraceaeND3007group | 0.2445 | 0.001337204 | 20.10768701 |
| 10 | 14137663 | rs72776675 | C | T | -0.064706741 | 0.014797022 | 8.72E-06 | 15302 | LachnospiraceaeND3007group | 0.175 | 0.00124813 | 19.12025384 |
| 3 | 172950553 | rs2861203 | A | G | 0.057230395 | 0.012727879 | 7.37E-06 | 15305 | LachnospiraceaeND3007group | 0.3191 | 0.001319273 | 20.21550649 |
| 11 | 3922055 | rs12362320 | C | G | 0.057316768 | 0.011546623 | 8.04E-07 | 16764 | LachnospiraceaeNK4A136group | 0.336 | 0.001467702 | 24.63778272 |
| 1 | 54578401 | rs954878 | G | A | -0.052065624 | 0.010908221 | 1.78E-06 | 17227 | LachnospiraceaeNK4A136group | 0.4503 | 0.00132072 | 22.7794852 |
| 8 | 4842929 | rs7832116 | G | A | -0.071475594 | 0.015170307 | 3.57E-06 | 17219 | LachnospiraceaeNK4A136group | 0.1461 | 0.001287536 | 22.19608311 |
| 3 | 190415145 | rs7616165 | T | G | -0.230541982 | 0.048346456 | 2.77E-06 | 4094 | LachnospiraceaeNK4A136group | 0.0368 | 0.005523539 | 22.7278596 |
| 3 | 161749981 | rs76193507 | G | A | -0.229730157 | 0.049977561 | 2.93E-06 | 3578 | LachnospiraceaeNK4A136group | 0.0567 | 0.005870681 | 21.11752782 |
| 17 | 35240097 | rs11263806 | G | A | -0.052461533 | 0.011675801 | 5.07E-06 | 17236 | LachnospiraceaeNK4A136group | 0.2783 | 0.00116994 | 20.18636735 |
| 19 | 51256120 | rs73044693 | G | A | -0.107579587 | 0.022988374 | 3.57E-06 | 12773 | LachnospiraceaeNK4A136group | 0.0666 | 0.001711616 | 21.89652704 |
| 5 | 6116659 | rs160061 | G | A | 0.051383089 | 0.010809407 | 2.12E-06 | 17234 | LachnospiraceaeNK4A136group | 0.4682 | 0.001309428 | 22.59364267 |
| 10 | 62175024 | rs7073658 | G | T | -0.049962145 | 0.010969284 | 5.27E-06 | 17236 | LachnospiraceaeNK4A136group | 0.4046 | 0.001202172 | 20.74317239 |
| 14 | 100177681 | rs68104925 | C | T | -0.054910353 | 0.011538497 | 2.37E-06 | 17227 | LachnospiraceaeNK4A136group | 0.3708 | 0.001312894 | 22.64433583 |
| 19 | 21806125 | rs12611395 | G | A | -0.090253198 | 0.019965236 | 5.83E-06 | 16486 | LachnospiraceaeNK4A136group | 0.0895 | 0.001238007 | 20.43259876 |
| 17 | 30016653 | rs2880566 | C | T | 0.059957809 | 0.013469261 | 5.61E-06 | 17232 | LachnospiraceaeNK4A136group | 0.1879 | 0.001148601 | 19.81315122 |
| 8 | 84735080 | rs28540839 | C | A | 0.050828488 | 0.011059105 | 9.34E-06 | 16764 | LachnospiraceaeNK4A136group | 0.4871 | 0.00125849 | 21.12139729 |
| 5 | 90289997 | rs59805249 | C | T | 0.09361625 | 0.020798243 | 9.45E-06 | 15195 | LachnospiraceaeNK4A136group | 0.0905 | 0.001331588 | 20.25778703 |
| 7 | 8497922 | rs10952110 | T | G | 0.048770868 | 0.010961435 | 9.08E-06 | 16764 | LachnospiraceaeNK4A136group | 0.4483 | 0.001179494 | 19.79403333 |
| 3 | 55181375 | rs4955932 | C | T | -0.049228303 | 0.010938782 | 7.05E-06 | 17236 | LachnospiraceaeNK4A136group | 0.3956 | 0.001173668 | 20.25076536 |
| 3 | 42568440 | rs437876 | C | T | 0.078463676 | 0.014476767 | 7.17E-08 | 10150 | LachnospiraceaeUCG001 | 0.3728 | 0.002885845 | 29.3703115 |
| 3 | 148269083 | rs985416 | T | C | 0.097025845 | 0.018180694 | 1.46E-07 | 9917 | LachnospiraceaeUCG001 | 0.2058 | 0.002863704 | 28.47517137 |
| 1 | 82384407 | rs2050911 | A | G | 0.075146621 | 0.015393431 | 1.11E-06 | 10148 | LachnospiraceaeUCG001 | 0.3042 | 0.002342874 | 23.82662222 |
| 9 | 7273249 | rs10815577 | G | C | -0.06837061 | 0.014369051 | 1.72E-06 | 10149 | LachnospiraceaeUCG001 | 0.3867 | 0.002225829 | 22.63586787 |
| 9 | 14477851 | rs573933 | C | T | -0.107898495 | 0.023234631 | 3.11E-06 | 10150 | LachnospiraceaeUCG001 | 0.1024 | 0.002120174 | 21.56123554 |
| 1 | 53773248 | rs78848836 | G | A | -0.118867977 | 0.025975321 | 3.38E-06 | 9506 | LachnospiraceaeUCG001 | 0.0835 | 0.002198135 | 20.93709875 |
| 6 | 145006780 | rs9403580 | T | C | 0.107800527 | 0.022977722 | 3.47E-06 | 10095 | LachnospiraceaeUCG001 | 0.1093 | 0.002175583 | 22.00603429 |
| 17 | 36155074 | rs7213933 | A | T | -0.081626876 | 0.018380006 | 9.02E-06 | 9961 | LachnospiraceaeUCG001 | 0.1948 | 0.001976119 | 19.71913343 |
| 19 | 13962924 | rs8104225 | G | A | 0.089209817 | 0.019766483 | 8.04E-06 | 9908 | LachnospiraceaeUCG001 | 0.1491 | 0.002051581 | 20.36473641 |
| 16 | 79092359 | rs74034332 | A | G | 0.168045475 | 0.038263689 | 3.33E-06 | 6004 | LachnospiraceaeUCG001 | 0.0457 | 0.003202185 | 19.28125379 |
| 7 | 96965504 | rs62496417 | G | T | -0.074850403 | 0.016568056 | 5.88E-06 | 10150 | LachnospiraceaeUCG001 | 0.2714 | 0.002006812 | 20.40608035 |
| 12 | 56256262 | rs2371284 | C | T | -0.076189302 | 0.017012675 | 7.56E-06 | 9961 | LachnospiraceaeUCG001 | 0.2227 | 0.002009399 | 20.05190104 |
| 1 | 166199362 | rs12131224 | T | C | 0.117095415 | 0.025909955 | 7.40E-06 | 9676 | LachnospiraceaeUCG001 | 0.1163 | 0.002106371 | 20.42004522 |
| 14 | 21455973 | rs4981345 | C | T | -0.068197297 | 0.014983138 | 6.09E-06 | 9961 | LachnospiraceaeUCG001 | 0.3509 | 0.002075504 | 20.71293056 |
| 1 | 108102470 | rs79476906 | A | T | -0.087088012 | 0.019700842 | 8.27E-06 | 9917 | LachnospiraceaeUCG001 | 0.1441 | 0.001966581 | 19.53707643 |
| 8 | 56839500 | rs7341608 | C | T | -0.078475713 | 0.017764768 | 9.48E-06 | 9917 | LachnospiraceaeUCG001 | 0.1899 | 0.001963891 | 19.51029513 |
| 1 | 240359089 | rs12747809 | A | G | -0.062197002 | 0.012568463 | 8.65E-07 | 14464 | LachnospiraceaeUCG004 | 0.34 | 0.001690252 | 24.48581236 |
| 2 | 49904589 | rs2882478 | A | G | -0.057709061 | 0.011833839 | 1.21E-06 | 14466 | LachnospiraceaeUCG004 | 0.4553 | 0.00164125 | 23.7780721 |
| 7 | 71303007 | rs12673420 | A | G | 0.055435065 | 0.011837515 | 2.98E-06 | 14463 | LachnospiraceaeUCG004 | 0.4414 | 0.00151402 | 21.92744179 |
| 6 | 81318836 | rs2444793 | T | C | -0.054243345 | 0.011818754 | 4.77E-06 | 14463 | LachnospiraceaeUCG004 | 0.4702 | 0.001454317 | 21.0615096 |
| 4 | 183247601 | rs2726805 | G | A | 0.054839706 | 0.01208444 | 6.30E-06 | 14466 | LachnospiraceaeUCG004 | 0.4085 | 0.001421579 | 20.59098596 |
| 3 | 70592215 | rs11128180 | G | A | 0.06482876 | 0.014012621 | 4.52E-06 | 14466 | LachnospiraceaeUCG004 | 0.2237 | 0.001477427 | 21.40112789 |
| 14 | 40536481 | rs12894272 | G | A | 0.057979201 | 0.012522898 | 4.34E-06 | 14463 | LachnospiraceaeUCG004 | 0.3002 | 0.001479903 | 21.43259117 |
| 6 | 14817584 | rs233486 | G | A | -0.079908769 | 0.017765984 | 6.28E-06 | 13710 | LachnospiraceaeUCG004 | 0.1243 | 0.00147344 | 20.22772248 |
| 12 | 24572337 | rs35182105 | G | A | -0.109724303 | 0.024221006 | 4.87E-06 | 13170 | LachnospiraceaeUCG004 | 0.0726 | 0.00155582 | 20.51896048 |
| 1 | 65990422 | rs6656451 | T | C | -0.054359986 | 0.01194699 | 5.57E-06 | 14458 | LachnospiraceaeUCG004 | 0.4394 | 0.001429921 | 20.70053729 |
| 1 | 105287469 | rs12072562 | C | T | 0.133103727 | 0.030356565 | 7.07E-06 | 9176 | LachnospiraceaeUCG004 | 0.0517 | 0.002090802 | 19.22120071 |
| 13 | 51292902 | rs2706242 | C | G | -0.09013993 | 0.019971219 | 9.84E-06 | 14026 | LachnospiraceaeUCG004 | 0.0934 | 0.001450311 | 20.36870146 |
| 3 | 154242406 | rs7629954 | G | A | 0.108440892 | 0.023824987 | 5.77E-06 | 12568 | LachnospiraceaeUCG004 | 0.0686 | 0.001645657 | 20.71341482 |
| 11 | 74391775 | rs10793103 | T | C | 0.097434405 | 0.018127922 | 9.35E-08 | 7014 | LachnospiraceaeUCG008 | 0.3042 | 0.004101832 | 28.88050603 |
| 3 | 100496680 | rs9873555 | C | G | -0.121161831 | 0.023346742 | 2.41E-07 | 6577 | LachnospiraceaeUCG008 | 0.1571 | 0.004078274 | 26.92445925 |
| 2 | 168628871 | rs13024781 | C | T | -0.079876878 | 0.016884555 | 2.29E-06 | 7016 | LachnospiraceaeUCG008 | 0.503 | 0.00317973 | 22.37376803 |
| 11 | 19572557 | rs10741777 | C | T | -0.097384219 | 0.019479136 | 7.69E-07 | 7014 | LachnospiraceaeUCG008 | 0.2614 | 0.003550808 | 24.98699003 |
| 16 | 84992314 | rs955844 | C | A | 0.112064162 | 0.022836934 | 1.81E-06 | 6863 | LachnospiraceaeUCG008 | 0.1998 | 0.003496413 | 24.073062 |
| 4 | 114220647 | rs67078837 | C | T | -0.08458483 | 0.017069269 | 7.68E-07 | 7016 | LachnospiraceaeUCG008 | 0.4583 | 0.003487773 | 24.54885808 |
| 2 | 228100140 | rs62277846 | T | C | 0.102292484 | 0.021231902 | 1.59E-06 | 7016 | LachnospiraceaeUCG008 | 0.2356 | 0.003297506 | 23.20522831 |
| 1 | 90818872 | rs10801803 | A | G | -0.117029089 | 0.024308454 | 1.40E-06 | 7017 | LachnospiraceaeUCG008 | 0.162 | 0.003292224 | 23.17123402 |
| 12 | 129539818 | rs61944774 | G | A | 0.179838121 | 0.039379746 | 6.34E-06 | 6000 | LachnospiraceaeUCG008 | 0.0577 | 0.003463853 | 20.84840848 |
| 15 | 31697284 | rs75356640 | A | G | 0.136523454 | 0.030312828 | 9.83E-06 | 6423 | LachnospiraceaeUCG008 | 0.0885 | 0.003148142 | 20.27805896 |
| 8 | 23071054 | rs57254474 | A | G | 0.08865115 | 0.019904502 | 6.92E-06 | 7017 | LachnospiraceaeUCG008 | 0.2406 | 0.002818958 | 19.83089479 |
| 6 | 81214803 | rs57091572 | G | A | -0.110442734 | 0.023580145 | 2.86E-06 | 6950 | LachnospiraceaeUCG008 | 0.1402 | 0.003146501 | 21.930896 |
| 10 | 83848397 | rs11192447 | G | A | 0.126592141 | 0.024345002 | 4.69E-07 | 12695 | LachnospiraceaeUCG010 | 0.0746 | 0.002125383 | 27.0349422 |
| 21 | 44079875 | rs9981767 | C | A | 0.065506222 | 0.01319942 | 9.96E-07 | 12816 | LachnospiraceaeUCG010 | 0.3161 | 0.00191809 | 24.62564425 |
| 14 | 29872341 | rs74315802 | T | G | 0.086726244 | 0.018347717 | 3.19E-06 | 12715 | LachnospiraceaeUCG010 | 0.1213 | 0.001754116 | 22.3392561 |
| 9 | 92267239 | rs12346653 | T | C | 0.065768558 | 0.013956927 | 2.70E-06 | 12816 | LachnospiraceaeUCG010 | 0.2744 | 0.001729628 | 22.20185443 |
| 19 | 42586245 | rs10414815 | C | T | 0.104529604 | 0.023025257 | 4.24E-06 | 11564 | LachnospiraceaeUCG010 | 0.1193 | 0.00177905 | 20.60603959 |
| 1 | 224897491 | rs72761829 | T | A | 0.111883594 | 0.023889286 | 2.58E-06 | 11488 | LachnospiraceaeUCG010 | 0.0716 | 0.001905696 | 21.93061741 |
| 2 | 185862591 | rs72894957 | A | G | 0.222262976 | 0.048642511 | 5.68E-06 | 4316 | LachnospiraceaeUCG010 | 0.0477 | 0.00481421 | 20.86896832 |
| 5 | 8146371 | rs336138 | T | G | 0.077954271 | 0.017186539 | 7.48E-06 | 13236 | LachnospiraceaeUCG010 | 0.163 | 0.001551928 | 20.57014374 |
| 7 | 81410263 | rs4576377 | C | A | -0.057200131 | 0.012704307 | 7.63E-06 | 13225 | LachnospiraceaeUCG010 | 0.3837 | 0.001530493 | 20.26872396 |
| 21 | 33193567 | rs2833528 | T | C | -0.056214807 | 0.012776299 | 9.92E-06 | 13233 | LachnospiraceaeUCG010 | 0.3608 | 0.001460825 | 19.35644925 |
| 9 | 85576178 | rs2153460 | T | A | -0.068403784 | 0.015656159 | 9.17E-06 | 13236 | LachnospiraceaeUCG010 | 0.1839 | 0.001440148 | 19.08640652 |
| 6 | 84772845 | rs17730011 | A | G | -0.070235733 | 0.015706022 | 7.85E-06 | 13226 | LachnospiraceaeUCG010 | 0.2187 | 0.001509729 | 19.99484952 |
| 19 | 6928017 | rs921925 | C | A | 0.098507745 | 0.020322897 | 9.72E-07 | 6952 | Lactobacillus | 0.2624 | 0.003368174 | 23.48792131 |
| 1 | 18501459 | rs16861661 | A | G | -0.183146977 | 0.038148013 | 1.28E-06 | 5325 | Lactobacillus | 0.0497 | 0.004309831 | 23.04053248 |
| 8 | 69010103 | rs768253 | G | T | -0.079195015 | 0.017179101 | 4.25E-06 | 6958 | Lactobacillus | 0.3907 | 0.00304499 | 21.24564657 |
| 2 | 28903275 | rs11674854 | T | C | -0.085268613 | 0.017650364 | 1.59E-06 | 6655 | Lactobacillus | 0.4274 | 0.003494642 | 23.33138548 |
| 16 | 73890910 | rs328312 | A | T | 0.081509206 | 0.016944121 | 1.41E-06 | 6950 | Lactobacillus | 0.4473 | 0.003318538 | 23.13397502 |
| 20 | 54192992 | rs6092149 | T | A | -0.080134426 | 0.01714896 | 3.29E-06 | 6906 | Lactobacillus | 0.4433 | 0.003151847 | 21.82915349 |
| 6 | 151027636 | rs75127669 | A | C | 0.13978419 | 0.031041446 | 6.83E-06 | 6840 | Lactobacillus | 0.0706 | 0.002955905 | 20.27240237 |
| 3 | 173151665 | rs77478751 | G | A | -0.219885755 | 0.047575911 | 7.33E-06 | 3669 | Lactobacillus | 0.0537 | 0.005788301 | 21.34927652 |
| 2 | 135755629 | rs1530559 | A | G | 0.080400057 | 0.017820739 | 4.93E-06 | 6655 | Lactobacillus | 0.5676 | 0.003049207 | 20.34842239 |
| 4 | 109896462 | rs62314653 | A | C | 0.187691889 | 0.039458456 | 2.24E-06 | 5944 | Lactobacillus | 0.0626 | 0.003792119 | 22.61854594 |
| 13 | 23834968 | rs7399658 | A | G | -0.107133701 | 0.022188263 | 3.12E-06 | 6613 | Lactobacillus | 0.2157 | 0.003513005 | 23.30634944 |
| 2 | 199312684 | rs12693845 | T | C | -0.080544718 | 0.017742876 | 8.96E-06 | 6958 | Lactobacillus | 0.3569 | 0.002952958 | 20.6016133 |
| 18 | 64699550 | rs34757988 | C | G | 0.122293892 | 0.022897605 | 8.95E-08 | 4055 | Lactococcus | 0.326 | 0.006985446 | 28.51117772 |
| 22 | 31532568 | rs757872 | C | G | 0.140799135 | 0.027581978 | 4.37E-07 | 3792 | Lactococcus | 0.2058 | 0.006825062 | 26.04474096 |
| 17 | 2699935 | rs123059 | C | T | -0.136709792 | 0.02746936 | 1.27E-06 | 3760 | Lactococcus | 0.2217 | 0.006544294 | 24.75546566 |
| 12 | 113161438 | rs4766997 | T | C | 0.114599246 | 0.023839024 | 2.06E-06 | 4053 | Lactococcus | 0.2962 | 0.005669448 | 23.09788506 |
| 19 | 28767353 | rs10417872 | G | T | 0.118305772 | 0.024521975 | 1.29E-06 | 4056 | Lactococcus | 0.2584 | 0.005705818 | 23.26412828 |
| 1 | 116887742 | rs6674304 | T | C | 0.200757712 | 0.044212138 | 6.18E-06 | 3869 | Lactococcus | 0.0686 | 0.005300959 | 20.60804935 |
| 13 | 113472525 | rs7992246 | C | T | 0.104232064 | 0.023078643 | 4.45E-06 | 4057 | Lactococcus | 0.3469 | 0.005002638 | 20.38768905 |
| 10 | 71518643 | rs55910161 | T | C | 0.146425945 | 0.030736694 | 2.36E-06 | 3836 | Lactococcus | 0.1352 | 0.005881409 | 22.6827269 |
| 2 | 31266049 | rs12621813 | A | G | 0.108422776 | 0.023997845 | 6.61E-06 | 4057 | Lactococcus | 0.2823 | 0.005006243 | 20.40245457 |
| 2 | 54114864 | rs2293361 | T | C | -0.199221089 | 0.043096892 | 1.40E-06 | 3787 | Lactococcus | 0.0646 | 0.005610993 | 21.35744466 |
| 7 | 14650221 | rs17168302 | A | G | 0.191856546 | 0.042476093 | 6.29E-06 | 4023 | Lactococcus | 0.0656 | 0.005045651 | 20.39145241 |
| 11 | 45826561 | rs61884471 | A | G | 0.124426313 | 0.024843116 | 1.01E-06 | 11559 | Marvinbryantia | 0.0845 | 0.002165462 | 25.08055773 |
| 10 | 12516924 | rs2724813 | G | A | -0.084076924 | 0.01675508 | 6.28E-07 | 11610 | Marvinbryantia | 0.2008 | 0.002164151 | 25.1759447 |
| 1 | 58441860 | rs1187983 | T | C | -0.093545581 | 0.019317443 | 2.02E-06 | 11896 | Marvinbryantia | 0.1223 | 0.001967393 | 23.44629677 |
| 6 | 132881664 | rs2842896 | T | C | -0.0649396 | 0.013114646 | 7.25E-07 | 11709 | Marvinbryantia | 0.5278 | 0.00208967 | 24.51499748 |
| 16 | 5938448 | rs11645029 | C | G | -0.060618043 | 0.013155125 | 4.15E-06 | 11709 | Marvinbryantia | 0.4523 | 0.001810119 | 21.22949419 |
| 18 | 46255637 | rs12963345 | C | G | -0.05968101 | 0.013228853 | 6.60E-06 | 11709 | Marvinbryantia | 0.4583 | 0.001735217 | 20.34950148 |
| 3 | 166039765 | rs2863363 | G | A | 0.063486062 | 0.013632279 | 3.11E-06 | 11950 | Marvinbryantia | 0.3678 | 0.001811609 | 21.68438643 |
| 11 | 79518683 | rs72948274 | C | A | -0.126353989 | 0.02722118 | 3.26E-06 | 11561 | Marvinbryantia | 0.0577 | 0.001860201 | 21.54214186 |
| 19 | 4542097 | rs146541147 | A | G | 0.118845391 | 0.02684231 | 6.86E-06 | 10778 | Marvinbryantia | 0.0706 | 0.001815505 | 19.59946993 |
| 13 | 86079090 | rs11620597 | C | T | 0.119479349 | 0.027168773 | 7.80E-06 | 11031 | Marvinbryantia | 0.0527 | 0.001750129 | 19.33601205 |
| 14 | 21577837 | rs8006832 | T | G | -0.095241547 | 0.021669867 | 6.58E-06 | 11412 | Marvinbryantia | 0.1113 | 0.001689834 | 19.3136456 |
| 1 | 211400180 | rs3125832 | C | A | 0.067932264 | 0.015011987 | 5.03E-06 | 11709 | Marvinbryantia | 0.2624 | 0.001745811 | 20.47394941 |
| 13 | 41963791 | rs76029318 | C | T | 0.22284888 | 0.045431939 | 1.08E-06 | 3387 | Methanobrevibacter | 0.0646 | 0.00705357 | 24.0459445 |
| 2 | 125440268 | rs10202904 | G | T | -0.112811092 | 0.023910624 | 3.09E-06 | 3583 | Methanobrevibacter | 0.4672 | 0.006174264 | 22.24740088 |
| 4 | 104418307 | rs894996 | A | C | 0.214212565 | 0.045604463 | 3.82E-06 | 3479 | Methanobrevibacter | 0.0716 | 0.006301954 | 22.05085639 |
| 11 | 89349287 | rs11018665 | T | A | 0.113013025 | 0.025434251 | 7.03E-06 | 3586 | Methanobrevibacter | 0.341 | 0.005475507 | 19.73225968 |
| 10 | 112266046 | rs1334944 | C | T | 0.115196507 | 0.025549236 | 7.61E-06 | 3586 | Methanobrevibacter | 0.2853 | 0.005637121 | 20.3179781 |
| 3 | 15053083 | rs6776814 | C | T | -0.188956311 | 0.041990543 | 8.05E-06 | 3283 | Methanobrevibacter | 0.0895 | 0.006130253 | 20.23742126 |
| 15 | 31516919 | rs4779844 | C | G | 0.109638988 | 0.024797947 | 9.28E-06 | 3585 | Methanobrevibacter | 0.3241 | 0.005423102 | 19.5369264 |
| 19 | 52925820 | rs4802933 | G | A | -0.135628431 | 0.030813872 | 9.74E-06 | 3586 | Methanobrevibacter | 0.1779 | 0.005373517 | 19.36273207 |
| 15 | 58063881 | rs503751 | G | C | 0.061829433 | 0.011888659 | 2.08E-07 | 14218 | Odoribacter | 0.5199 | 0.00189872 | 27.04355147 |
| 12 | 67655943 | rs77779484 | A | G | -0.133489405 | 0.026852607 | 6.56E-07 | 11935 | Odoribacter | 0.0586 | 0.002066331 | 24.70858583 |
| 19 | 49523088 | rs10423795 | T | C | 0.055069483 | 0.012116411 | 6.58E-06 | 14135 | Odoribacter | 0.4066 | 0.001459298 | 20.65439845 |
| 4 | 11907559 | rs6856150 | A | G | 0.088192184 | 0.019414623 | 6.06E-06 | 14597 | Odoribacter | 0.1183 | 0.001411644 | 20.63206809 |
| 8 | 1266824 | rs10093869 | G | A | -0.057779486 | 0.012538929 | 3.67E-06 | 14218 | Odoribacter | 0.3757 | 0.001491214 | 21.23075212 |
| 17 | 46763962 | rs4793970 | G | A | -0.057632938 | 0.012915412 | 6.03E-06 | 14687 | Odoribacter | 0.3012 | 0.001353953 | 19.90975195 |
| 8 | 69677480 | rs74553962 | G | T | 0.121448819 | 0.026410563 | 9.49E-06 | 11803 | Odoribacter | 0.0626 | 0.001788387 | 21.14256517 |
| 14 | 70944224 | rs28417404 | G | A | -0.072689645 | 0.016137195 | 3.68E-06 | 14698 | Odoribacter | 0.169 | 0.001378579 | 20.28756278 |
| 11 | 6848360 | rs16918425 | T | A | 0.099978081 | 0.022378451 | 8.85E-06 | 13689 | Odoribacter | 0.0746 | 0.001455945 | 19.95657831 |
| 2 | 150077190 | rs1035588 | G | A | -0.108148115 | 0.023684753 | 4.86E-06 | 3739 | Olsenella | 0.3827 | 0.00554536 | 20.83856609 |
| 19 | 4925018 | rs62112538 | T | C | -0.199430349 | 0.040703259 | 1.19E-06 | 3630 | Olsenella | 0.0805 | 0.006569841 | 23.99301297 |
| 1 | 247642270 | rs35225860 | G | A | -0.223603655 | 0.048239131 | 3.87E-06 | 3630 | Olsenella | 0.0567 | 0.005884224 | 21.47432431 |
| 9 | 21884425 | rs72691585 | A | C | -0.249078856 | 0.052081416 | 2.95E-06 | 3078 | Olsenella | 0.0686 | 0.007376057 | 22.85734995 |
| 6 | 10422207 | rs9460691 | A | C | 0.119965803 | 0.026864494 | 7.28E-06 | 3739 | Olsenella | 0.2167 | 0.005305081 | 19.93082274 |
| 10 | 10777085 | rs17148768 | A | G | 0.140434164 | 0.029560175 | 2.20E-06 | 3648 | Olsenella | 0.2117 | 0.006148909 | 22.55762568 |
| 4 | 174810498 | rs61090148 | G | A | -0.104782706 | 0.023134258 | 6.44E-06 | 3739 | Olsenella | 0.4225 | 0.005456778 | 20.50386427 |
| 1 | 179629191 | rs7540303 | T | C | 0.108043794 | 0.023637952 | 5.32E-06 | 3739 | Olsenella | 0.4215 | 0.005556542 | 20.88082192 |
| 1 | 231960352 | rs2759329 | A | G | -0.111131408 | 0.02372184 | 3.43E-06 | 3739 | Olsenella | 0.34 | 0.005835521 | 21.93534698 |
| 20 | 19969568 | rs6046522 | T | C | 0.12293822 | 0.026992758 | 4.48E-06 | 3705 | Olsenella | 0.2863 | 0.005567579 | 20.73217318 |
| 17 | 59632873 | rs8066522 | A | G | -0.106528718 | 0.02403789 | 9.70E-06 | 3739 | Olsenella | 0.3469 | 0.005225285 | 19.62945927 |
| 1 | 184942671 | rs234108 | G | A | 0.074955279 | 0.015263201 | 9.16E-07 | 8694 | Oscillibacter | 0.4801 | 0.002766244 | 24.11089102 |
| 14 | 32270129 | rs36095275 | T | C | -0.07523683 | 0.015686113 | 1.40E-06 | 8694 | Oscillibacter | 0.3618 | 0.002639143 | 23.0001334 |
| 14 | 21479605 | rs11627628 | C | T | 0.143960728 | 0.029022314 | 1.01E-06 | 8486 | Oscillibacter | 0.0726 | 0.002891103 | 24.59923724 |
| 6 | 28580593 | rs9393920 | G | A | -0.074466242 | 0.015108116 | 9.92E-07 | 8930 | Oscillibacter | 0.4354 | 0.002713107 | 24.28851548 |
| 22 | 44834707 | rs133832 | C | A | -0.079552688 | 0.016241059 | 1.15E-06 | 8926 | Oscillibacter | 0.3131 | 0.002680763 | 23.9874296 |
| 4 | 3656291 | rs12649930 | G | T | 0.121588701 | 0.025961173 | 4.09E-06 | 8794 | Oscillibacter | 0.0944 | 0.002488109 | 21.93001953 |
| 10 | 116469806 | rs75453768 | T | G | 0.122120078 | 0.026862946 | 5.35E-06 | 8256 | Oscillibacter | 0.0865 | 0.002496959 | 20.66149079 |
| 2 | 179457147 | rs16866406 | G | A | 0.098876509 | 0.02087954 | 3.08E-06 | 8930 | Oscillibacter | 0.1392 | 0.002504977 | 22.42059458 |
| 20 | 49507892 | rs761240 | G | T | -0.176639573 | 0.038881196 | 2.04E-06 | 5185 | Oscillibacter | 0.0666 | 0.003964813 | 20.63142482 |
| 8 | 21598077 | rs4506202 | G | A | -0.071132334 | 0.01522611 | 3.21E-06 | 8694 | Oscillibacter | 0.5159 | 0.002504077 | 21.82007689 |
| 11 | 79013843 | rs61883564 | G | A | -0.1013514 | 0.022101565 | 3.39E-06 | 8930 | Oscillibacter | 0.1243 | 0.002349311 | 21.02404068 |
| 9 | 1798324 | rs16934185 | G | A | -0.129567532 | 0.028156999 | 4.38E-06 | 8492 | Oscillibacter | 0.1044 | 0.002487299 | 21.1698282 |
| 11 | 25973451 | rs12417956 | G | C | 0.078543098 | 0.017407454 | 6.03E-06 | 8928 | Oscillibacter | 0.2445 | 0.002275109 | 20.35392863 |
| 6 | 36931994 | rs6901560 | G | C | 0.085503217 | 0.018693038 | 6.21E-06 | 8525 | Oscillibacter | 0.1988 | 0.002448196 | 20.91718455 |
| 20 | 18003390 | rs62206502 | A | C | -0.068142274 | 0.015134881 | 6.60E-06 | 8883 | Oscillibacter | 0.4592 | 0.002276804 | 20.26643881 |
| 12 | 82366390 | rs137917150 | A | T | -0.174992153 | 0.038839403 | 4.62E-06 | 5733 | Oscillibacter | 0.0616 | 0.003528378 | 20.29273208 |
| 8 | 11116314 | rs11990279 | C | T | -0.082493414 | 0.018045748 | 4.94E-06 | 8528 | Oscillibacter | 0.2396 | 0.002444436 | 20.89233141 |
| 6 | 18093691 | rs12206468 | A | G | -0.133015789 | 0.026973335 | 1.04E-06 | 9554 | Oscillospira | 0.0636 | 0.002538913 | 24.31342677 |
| 3 | 172373615 | rs73038677 | A | T | -0.083210036 | 0.016866557 | 1.09E-06 | 10195 | Oscillospira | 0.1819 | 0.002381638 | 24.33398969 |
| 14 | 28151415 | rs1954532 | C | T | -0.082624583 | 0.017525166 | 2.27E-06 | 9841 | Oscillospira | 0.2087 | 0.002253592 | 22.22316923 |
| 4 | 89483300 | rs28889936 | C | A | 0.114050943 | 0.025283847 | 3.37E-06 | 9758 | Oscillospira | 0.1083 | 0.002080875 | 20.34334865 |
| 1 | 76803093 | rs751183 | C | T | -0.07743224 | 0.017221553 | 6.85E-06 | 9841 | Oscillospira | 0.2455 | 0.002050069 | 20.21206888 |
| 17 | 14881860 | rs8076323 | G | A | 0.07153922 | 0.015653872 | 5.61E-06 | 10193 | Oscillospira | 0.2575 | 0.002044814 | 20.88140158 |
| 6 | 56160483 | rs72866977 | C | A | -0.130569033 | 0.028167414 | 5.63E-06 | 9098 | Oscillospira | 0.0696 | 0.002356221 | 21.48280332 |
| 6 | 170474630 | rs62422654 | T | C | 0.089845572 | 0.019808815 | 6.47E-06 | 9769 | Oscillospira | 0.1471 | 0.002101419 | 20.56778078 |
| 16 | 89792856 | rs12925026 | C | T | 0.135590113 | 0.030677984 | 9.31E-06 | 8200 | Oscillospira | 0.0726 | 0.002376595 | 19.52974048 |
| 3 | 171947435 | rs4428215 | A | G | 0.130292636 | 0.0242237 | 7.51E-08 | 4655 | Oxalobacter | 0.2604 | 0.006176584 | 28.91825908 |
| 9 | 87514407 | rs736744 | T | C | 0.117882083 | 0.021126239 | 2.57E-08 | 4655 | Oxalobacter | 0.4155 | 0.006644104 | 31.12179208 |
| 22 | 37421469 | rs6000536 | T | C | -0.130991749 | 0.025380356 | 2.06E-07 | 4654 | Oxalobacter | 0.2107 | 0.005690985 | 26.62599115 |
| 4 | 189935314 | rs36057338 | T | G | 0.207847028 | 0.042143922 | 8.80E-07 | 4244 | Oxalobacter | 0.0755 | 0.005698499 | 24.31157111 |
| 20 | 59502888 | rs6071435 | A | T | -0.105512319 | 0.021488964 | 1.07E-06 | 4635 | Oxalobacter | 0.3638 | 0.005174549 | 24.09838559 |
| 9 | 19682558 | rs12002250 | C | A | 0.217122385 | 0.046631729 | 1.42E-06 | 4297 | Oxalobacter | 0.0596 | 0.005019902 | 21.66925456 |
| 6 | 38550301 | rs1569853 | C | T | -0.138077533 | 0.029698126 | 3.65E-06 | 4492 | Oxalobacter | 0.1382 | 0.004789203 | 21.60700277 |
| 12 | 96819204 | rs11108500 | G | A | -0.199099074 | 0.04273266 | 3.74E-06 | 4303 | Oxalobacter | 0.0765 | 0.005019516 | 21.69785072 |
| 8 | 20902693 | rs10464997 | A | G | 0.137690928 | 0.029480352 | 3.30E-06 | 4650 | Oxalobacter | 0.1531 | 0.004669386 | 21.8051209 |
| 15 | 93941937 | rs111966731 | C | T | 0.213114454 | 0.047162014 | 7.30E-06 | 3931 | Oxalobacter | 0.0716 | 0.00516759 | 20.40892435 |
| 8 | 115560689 | rs6993398 | A | G | 0.127217146 | 0.027885461 | 7.13E-06 | 4656 | Oxalobacter | 0.1531 | 0.004450262 | 20.80410533 |
| 11 | 126582578 | rs3862635 | T | C | -0.172142003 | 0.039402609 | 9.19E-06 | 4469 | Oxalobacter | 0.0785 | 0.004252678 | 19.07784623 |
| 9 | 2217340 | rs60884758 | T | C | -0.07026666 | 0.014224884 | 5.71E-07 | 17293 | Parabacteroides | 0.165 | 0.001409024 | 24.39781297 |
| 5 | 169360155 | rs3860755 | C | G | 0.055878381 | 0.011662454 | 1.71E-06 | 17294 | Parabacteroides | 0.3082 | 0.001325672 | 22.9539552 |
| 6 | 47368687 | rs4236095 | A | G | 0.076196085 | 0.015704151 | 1.93E-06 | 16383 | Parabacteroides | 0.1252 | 0.001434892 | 23.53874893 |
| 3 | 191572365 | rs115602804 | A | G | 0.10308013 | 0.02227358 | 1.93E-06 | 16394 | Parabacteroides | 0.0666 | 0.001304722 | 21.4149415 |
| 3 | 52354847 | rs114567323 | C | T | 0.186477784 | 0.040519936 | 5.65E-06 | 5859 | Parabacteroides | 0.0467 | 0.003601854 | 21.17231867 |
| 12 | 56396953 | rs7298818 | T | C | 0.088883151 | 0.02009019 | 8.54E-06 | 16510 | Parabacteroides | 0.0765 | 0.001184157 | 19.57123344 |
| 1 | 85585609 | rs6657302 | C | T | -0.104519671 | 0.022552116 | 9.76E-06 | 16552 | Parabacteroides | 0.0517 | 0.001296008 | 21.4767716 |
| 6 | 165969505 | rs11965579 | C | G | 0.16282395 | 0.038186321 | 8.87E-06 | 3890 | Parabacteroides | 0.0348 | 0.00465207 | 18.1717846 |
| 16 | 7045276 | rs17141986 | C | G | 0.050030286 | 0.011145925 | 7.27E-06 | 16825 | Parabacteroides | 0.4274 | 0.001196076 | 20.14568802 |
| 2 | 38037203 | rs72893646 | T | A | -0.071559422 | 0.015666777 | 8.83E-06 | 16508 | Parabacteroides | 0.1282 | 0.001262209 | 20.86035679 |
| 5 | 174606261 | rs2081023 | G | A | -0.122560702 | 0.023650724 | 2.64E-07 | 7178 | Paraprevotella | 0.1024 | 0.003727249 | 26.84680291 |
| 17 | 69135631 | rs9900242 | G | A | -0.085296669 | 0.017521167 | 1.14E-06 | 6900 | Paraprevotella | 0.3608 | 0.003422945 | 23.69257058 |
| 13 | 86162420 | rs9602779 | C | A | -0.10669184 | 0.02202635 | 6.93E-07 | 7177 | Paraprevotella | 0.173 | 0.003258494 | 23.45612664 |
| 3 | 149298792 | rs140997932 | C | T | -0.162375604 | 0.035417814 | 2.11E-06 | 6187 | Paraprevotella | 0.0646 | 0.003385673 | 21.01152844 |
| 11 | 114526679 | rs145020347 | G | A | -0.124650032 | 0.026232336 | 4.03E-06 | 6859 | Paraprevotella | 0.1392 | 0.003281125 | 22.57273593 |
| 12 | 114131978 | rs4767113 | T | C | 0.088246646 | 0.018381962 | 2.14E-06 | 6900 | Paraprevotella | 0.2873 | 0.003329012 | 23.0402279 |
| 1 | 195971173 | rs3008582 | C | T | 0.105720143 | 0.022724571 | 4.36E-06 | 7175 | Paraprevotella | 0.1322 | 0.00300742 | 21.63729719 |
| 11 | 41079703 | rs4756632 | T | G | -0.138905488 | 0.028989683 | 3.82E-06 | 7179 | Paraprevotella | 0.0934 | 0.003187875 | 22.95254638 |
| 12 | 25249743 | rs10842464 | C | T | -0.075821491 | 0.017256778 | 6.60E-06 | 7178 | Paraprevotella | 0.4185 | 0.002682226 | 19.29941644 |
| 7 | 81759508 | rs3801748 | A | G | 0.077972078 | 0.01716912 | 5.20E-06 | 7179 | Paraprevotella | 0.3459 | 0.002864655 | 20.61869383 |
| 18 | 69623804 | rs7240324 | G | T | -0.102290408 | 0.022694788 | 5.96E-06 | 6900 | Paraprevotella | 0.1909 | 0.002935565 | 20.30914612 |
| 12 | 71978728 | rs17109926 | G | A | -0.098833506 | 0.021617178 | 6.75E-06 | 7179 | Paraprevotella | 0.1879 | 0.002903246 | 20.89726338 |
| 6 | 83459314 | rs17785622 | G | A | 0.248065378 | 0.052430396 | 1.93E-06 | 3288 | Paraprevotella | 0.0596 | 0.006762191 | 22.37184383 |
| 20 | 25599776 | rs2387977 | C | T | -0.068177776 | 0.013475556 | 5.38E-07 | 11345 | Parasutterella | 0.4583 | 0.002251174 | 25.59267774 |
| 2 | 72235444 | rs7572229 | A | G | 0.066272974 | 0.013273581 | 6.32E-07 | 11383 | Parasutterella | 0.4901 | 0.002185194 | 24.92415277 |
| 4 | 15565736 | rs35414597 | A | T | -0.068494619 | 0.01421433 | 1.51E-06 | 11386 | Parasutterella | 0.339 | 0.002035186 | 23.21580867 |
| 4 | 65556925 | rs6828768 | T | C | 0.063685185 | 0.013264516 | 1.78E-06 | 11386 | Parasutterella | 0.5 | 0.002020433 | 23.04717468 |
| 10 | 44293839 | rs10899911 | G | A | -0.071710162 | 0.014815144 | 1.15E-06 | 11386 | Parasutterella | 0.2724 | 0.002053458 | 23.42466209 |
| 12 | 5275540 | rs7303158 | T | C | 0.064685912 | 0.013425592 | 1.33E-06 | 11386 | Parasutterella | 0.4423 | 0.002034683 | 23.2100571 |
| 2 | 178954708 | rs78383039 | C | T | -0.146314647 | 0.029711928 | 1.57E-06 | 9174 | Parasutterella | 0.0666 | 0.002636384 | 24.24483522 |
| 8 | 114804024 | rs35055552 | C | T | 0.109554176 | 0.023543268 | 3.35E-06 | 9970 | Parasutterella | 0.0775 | 0.00216714 | 21.64897032 |
| 6 | 137615592 | rs2090816 | C | A | 0.084096728 | 0.017731494 | 2.90E-06 | 11385 | Parasutterella | 0.1571 | 0.001971866 | 22.49009275 |
| 12 | 126498824 | rs1403396 | T | A | -0.075672871 | 0.015903462 | 2.76E-06 | 11378 | Parasutterella | 0.2147 | 0.001985948 | 22.63709682 |
| 17 | 14692867 | rs55877868 | C | A | -0.10445781 | 0.022808932 | 2.87E-06 | 10588 | Parasutterella | 0.0964 | 0.001976963 | 20.96958703 |
| 15 | 67316307 | rs8039785 | G | T | 0.061834541 | 0.013300041 | 3.62E-06 | 11383 | Parasutterella | 0.4523 | 0.00189529 | 21.61125486 |
| 8 | 16674526 | rs823424 | A | G | -0.071344819 | 0.015695821 | 4.95E-06 | 11386 | Parasutterella | 0.2097 | 0.001811333 | 20.65763 |
| 12 | 53260710 | rs7311004 | C | T | -0.061752212 | 0.01364384 | 5.92E-06 | 11384 | Parasutterella | 0.3757 | 0.001796206 | 20.48120403 |
| 3 | 156550415 | rs62273907 | G | A | 0.229468131 | 0.050226185 | 5.88E-06 | 3691 | Parasutterella | 0.0497 | 0.0056233 | 20.86166611 |
| 3 | 193896709 | rs6809952 | A | G | -0.068495728 | 0.015089156 | 8.13E-06 | 11077 | Parasutterella | 0.2734 | 0.001856811 | 20.60244054 |
| 3 | 30163689 | rs11715853 | A | G | -0.066295189 | 0.014611398 | 6.23E-06 | 11386 | Parasutterella | 0.3141 | 0.001804782 | 20.58278662 |
| 3 | 17793504 | rs75754569 | G | C | 0.181434144 | 0.031942874 | 1.10E-08 | 5481 | Peptococcus | 0.1103 | 0.005851692 | 32.25013709 |
| 13 | 91182556 | rs77681628 | T | C | 0.200307214 | 0.038732756 | 2.69E-07 | 4842 | Peptococcus | 0.0586 | 0.005493124 | 26.73356971 |
| 4 | 35358594 | rs10031059 | C | T | -0.121166531 | 0.022584403 | 1.24E-07 | 5526 | Peptococcus | 0.2207 | 0.005181804 | 28.7733827 |
| 6 | 99020289 | rs6918730 | A | G | 0.135310568 | 0.028974201 | 1.15E-06 | 5656 | Peptococcus | 0.0974 | 0.003841136 | 21.8015269 |
| 14 | 57985112 | rs413827 | A | G | 0.11022947 | 0.023752348 | 3.30E-06 | 5482 | Peptococcus | 0.2127 | 0.003913276 | 21.52900044 |
| 2 | 33691809 | rs2054133 | A | G | 0.089543257 | 0.018833153 | 2.14E-06 | 5657 | Peptococcus | 0.4742 | 0.003980169 | 22.59779761 |
| 10 | 78716030 | rs11001941 | A | G | -0.195611127 | 0.039221681 | 1.33E-06 | 5607 | Peptococcus | 0.0676 | 0.004416536 | 24.86450169 |
| 9 | 104595848 | rs7033353 | G | T | 0.090152001 | 0.018995009 | 2.22E-06 | 5657 | Peptococcus | 0.4195 | 0.003966064 | 22.5173951 |
| 22 | 50973113 | rs5770862 | C | T | 0.162017845 | 0.035681339 | 3.22E-06 | 5431 | Peptococcus | 0.0666 | 0.003781969 | 20.61025917 |
| 15 | 75073172 | rs34282744 | C | G | 0.19179739 | 0.03998324 | 1.84E-06 | 5257 | Peptococcus | 0.0616 | 0.004358074 | 23.0019235 |
| 11 | 968356 | rs72850165 | C | T | -0.134304018 | 0.030042318 | 5.74E-06 | 5627 | Peptococcus | 0.1083 | 0.003539113 | 19.97821717 |
| 11 | 4019788 | rs11030569 | T | A | -0.174020126 | 0.037413336 | 3.13E-06 | 5329 | Peptococcus | 0.0775 | 0.004043345 | 21.62634164 |
| 6 | 162239191 | rs7766680 | C | G | 0.09768317 | 0.021415492 | 3.51E-06 | 5655 | Peptococcus | 0.2485 | 0.003665691 | 20.79839081 |
| 1 | 75971700 | rs74592222 | A | G | 0.137957011 | 0.030296488 | 8.55E-06 | 5329 | Peptococcus | 0.1173 | 0.003875884 | 20.7271675 |
| 20 | 43118017 | rs36121075 | G | A | -0.140670082 | 0.030627993 | 6.99E-06 | 5301 | Peptococcus | 0.1282 | 0.003963545 | 21.08640368 |
| 1 | 216223782 | rs12069354 | T | C | 0.167629782 | 0.037950049 | 9.28E-06 | 5352 | Peptococcus | 0.0666 | 0.003632296 | 19.50362553 |
| 12 | 53098742 | rs75882962 | C | T | 0.096866054 | 0.019057501 | 3.19E-07 | 10710 | Phascolarctobacterium | 0.167 | 0.002406442 | 25.83034395 |
| 4 | 183252836 | rs56157888 | C | A | 0.095486383 | 0.019397634 | 1.09E-06 | 10031 | Phascolarctobacterium | 0.17 | 0.002409868 | 24.22695255 |
| 3 | 168339417 | rs56069061 | A | G | -0.111305825 | 0.02306943 | 1.87E-06 | 11007 | Phascolarctobacterium | 0.1014 | 0.002110453 | 23.27464998 |
| 3 | 186553389 | rs74540770 | A | G | -0.121014393 | 0.025862072 | 3.60E-06 | 9403 | Phascolarctobacterium | 0.0646 | 0.002323116 | 21.89046718 |
| 17 | 73584441 | rs76124218 | G | C | -0.159360781 | 0.03445316 | 2.67E-06 | 7314 | Phascolarctobacterium | 0.0517 | 0.00291663 | 21.388779 |
| 1 | 203102563 | rs6427992 | C | G | -0.065245772 | 0.013750826 | 2.09E-06 | 11113 | Phascolarctobacterium | 0.3926 | 0.002021794 | 22.50966365 |
| 2 | 174436533 | rs12618201 | G | A | 0.06416571 | 0.013820192 | 3.38E-06 | 11113 | Phascolarctobacterium | 0.3469 | 0.001935998 | 21.55259957 |
| 22 | 47841258 | rs74847270 | G | A | -0.104889022 | 0.023099793 | 5.73E-06 | 11098 | Phascolarctobacterium | 0.0875 | 0.001854356 | 20.61415956 |
| 8 | 102434276 | rs1264476 | G | T | 0.076733542 | 0.016604148 | 4.30E-06 | 11104 | Phascolarctobacterium | 0.1998 | 0.001919656 | 21.35300813 |
| 14 | 21358155 | rs28525131 | A | G | -0.118657158 | 0.026904831 | 8.23E-06 | 10457 | Phascolarctobacterium | 0.0944 | 0.001856578 | 19.44662626 |
| 13 | 71376322 | rs7982713 | A | G | 0.072692152 | 0.016320086 | 9.72E-06 | 10710 | Phascolarctobacterium | 0.2286 | 0.001849 | 19.83577026 |
| 4 | 17051156 | rs11929846 | C | T | -0.069719918 | 0.015796922 | 8.88E-06 | 11114 | Phascolarctobacterium | 0.2097 | 0.001749596 | 19.47558999 |
| 3 | 60474379 | rs430270 | C | A | 0.13915522 | 0.029705527 | 2.87E-06 | 3318 | Prevotella7 | 0.2008 | 0.0065703 | 21.93121099 |
| 2 | 12101762 | rs57404562 | A | C | 0.15548647 | 0.031605691 | 6.22E-07 | 3487 | Prevotella7 | 0.1799 | 0.006892849 | 24.18830631 |
| 18 | 71442664 | rs9959718 | A | G | 0.133012021 | 0.027536133 | 1.90E-06 | 3489 | Prevotella7 | 0.2495 | 0.006643243 | 23.31990973 |
| 22 | 24613952 | rs9608249 | G | A | -0.158213329 | 0.033631703 | 2.07E-06 | 3482 | Prevotella7 | 0.171 | 0.006315506 | 22.11764588 |
| 2 | 242066314 | rs2240542 | T | C | 0.120849888 | 0.026177616 | 4.84E-06 | 3489 | Prevotella7 | 0.2575 | 0.006071368 | 21.30018142 |
| 10 | 133000320 | rs2918132 | T | C | -0.11464837 | 0.025491556 | 6.42E-06 | 3318 | Prevotella7 | 0.3678 | 0.006059365 | 20.21534722 |
| 11 | 19943115 | rs16937247 | C | G | 0.146097858 | 0.035152923 | 9.64E-06 | 3486 | Prevotella7 | 0.1402 | 0.004930499 | 17.26297219 |
| 16 | 47237609 | rs118038478 | G | A | 0.205675553 | 0.046891721 | 7.85E-06 | 3295 | Prevotella7 | 0.0755 | 0.005804826 | 19.22690168 |
| 1 | 3343802 | rs12124567 | G | A | -0.121281313 | 0.027501731 | 9.49E-06 | 3283 | Prevotella7 | 0.2624 | 0.005888867 | 19.43582884 |
| 6 | 91019654 | rs12195431 | C | T | 0.196527815 | 0.044232188 | 8.73E-06 | 3489 | Prevotella7 | 0.0785 | 0.005626261 | 19.7297775 |
| 1 | 29736216 | rs9426434 | C | T | -0.123657205 | 0.027851446 | 9.72E-06 | 3318 | Prevotella7 | 0.2734 | 0.005906014 | 19.70069338 |
| 11 | 39821773 | rs79263163 | C | A | -0.143981995 | 0.031500978 | 7.51E-06 | 3489 | Prevotella7 | 0.1849 | 0.00595216 | 20.87946119 |
| 19 | 639161 | rs111509883 | C | T | 0.171130632 | 0.03476241 | 1.24E-06 | 6960 | Prevotella9 | 0.0845 | 0.0034699 | 24.22763017 |
| 8 | 19115604 | rs2683313 | G | A | -0.072457582 | 0.015160279 | 1.69E-06 | 10260 | Prevotella9 | 0.3082 | 0.002221468 | 22.83855426 |
| 22 | 44236460 | rs117271932 | G | A | 0.20808857 | 0.044039277 | 2.82E-06 | 4878 | Prevotella9 | 0.0467 | 0.004556077 | 22.31711168 |
| 9 | 109009495 | rs10512344 | G | C | 0.247372806 | 0.054408651 | 3.19E-06 | 3063 | Prevotella9 | 0.0328 | 0.006703474 | 20.65781248 |
| 1 | 118852817 | rs9428102 | G | A | -0.07788445 | 0.017606807 | 4.62E-06 | 10264 | Prevotella9 | 0.2366 | 0.001902815 | 19.56391156 |
| 20 | 21470713 | rs746764 | C | T | -0.091577152 | 0.019324484 | 2.04E-06 | 9385 | Prevotella9 | 0.173 | 0.002387186 | 22.45256181 |
| 15 | 38513730 | rs16966465 | C | G | 0.074334818 | 0.01652906 | 9.33E-06 | 10261 | Prevotella9 | 0.2346 | 0.001967177 | 20.22104397 |
| 10 | 12497581 | rs2104588 | C | T | 0.105558491 | 0.023773023 | 8.13E-06 | 9268 | Prevotella9 | 0.1193 | 0.002122798 | 19.71168656 |
| 10 | 122819400 | rs11199734 | T | A | 0.077020901 | 0.016946218 | 7.00E-06 | 10263 | Prevotella9 | 0.2177 | 0.002008741 | 20.65318189 |
| 18 | 19993452 | rs7232121 | C | G | 0.067129188 | 0.014422959 | 3.76E-06 | 9790 | Prevotella9 | 0.4791 | 0.00220786 | 21.65834783 |
| 2 | 11232685 | rs11685699 | T | C | -0.141357386 | 0.029566474 | 2.03E-06 | 8342 | Prevotella9 | 0.0825 | 0.002732621 | 22.85250522 |
| 22 | 26152738 | rs9613013 | A | G | 0.091759081 | 0.020269664 | 6.10E-06 | 9790 | Prevotella9 | 0.1451 | 0.002088883 | 20.48878803 |
| 1 | 14161251 | rs2495052 | G | A | 0.083897846 | 0.018852315 | 8.97E-06 | 9790 | Prevotella9 | 0.1789 | 0.002018886 | 19.80083521 |
| 2 | 91954502 | rs72815774 | C | T | -0.176185788 | 0.039306766 | 8.78E-06 | 5728 | Prevotella9 | 0.0706 | 0.003495292 | 20.08424213 |
| 17 | 59421778 | rs4968431 | T | G | 0.064011283 | 0.014416226 | 8.58E-06 | 10271 | Prevotella9 | 0.3638 | 0.001915862 | 19.71175599 |
| 12 | 1794470 | rs7976209 | C | T | -0.087078586 | 0.019795052 | 7.28E-06 | 10263 | Prevotella9 | 0.167 | 0.001881989 | 19.34749793 |
| 4 | 161115744 | rs12648235 | C | T | 0.078590196 | 0.017767553 | 7.39E-06 | 10271 | Prevotella9 | 0.1571 | 0.001901263 | 19.56126499 |
| 5 | 63736025 | rs1304512 | A | G | 0.076159345 | 0.016588486 | 5.29E-06 | 9790 | Prevotella9 | 0.2187 | 0.002148405 | 21.07386477 |
| 18 | 71992502 | rs7237249 | T | C | -0.082376407 | 0.018212594 | 8.93E-06 | 10271 | Prevotella9 | 0.1352 | 0.001987857 | 20.45396435 |
| 9 | 112657543 | rs2900503 | T | G | -0.172329153 | 0.032667127 | 1.55E-07 | 3320 | RikenellaceaeRC9gutgroup | 0.171 | 0.008312514 | 27.81210926 |
| 4 | 149562638 | rs17582787 | G | A | -0.157739395 | 0.033992444 | 3.55E-06 | 3320 | RikenellaceaeRC9gutgroup | 0.1292 | 0.006444211 | 21.52057445 |
| 11 | 102429543 | rs7113155 | C | G | 0.114422634 | 0.024779825 | 5.26E-06 | 3334 | RikenellaceaeRC9gutgroup | 0.4523 | 0.006354673 | 21.3091839 |
| 4 | 84698188 | rs4270579 | A | G | -0.118021261 | 0.027104126 | 5.46E-06 | 3331 | RikenellaceaeRC9gutgroup | 0.3042 | 0.005659916 | 18.94911179 |
| 10 | 135010198 | rs2998141 | C | T | -0.136346427 | 0.029307262 | 4.42E-06 | 3330 | RikenellaceaeRC9gutgroup | 0.2217 | 0.006457719 | 21.63097541 |
| 7 | 73334987 | rs4717843 | T | G | -0.119376102 | 0.026054834 | 4.72E-06 | 3220 | RikenellaceaeRC9gutgroup | 0.3618 | 0.006477094 | 20.97917154 |
| 1 | 164800873 | rs9887954 | A | G | -0.114849386 | 0.024901293 | 4.81E-06 | 3330 | RikenellaceaeRC9gutgroup | 0.4364 | 0.006347517 | 21.25948065 |
| 16 | 76605358 | rs7193937 | C | G | 0.12415332 | 0.027741031 | 6.19E-06 | 3220 | RikenellaceaeRC9gutgroup | 0.2893 | 0.006181908 | 20.0171229 |
| 6 | 165668732 | rs80309088 | A | G | 0.174049957 | 0.038342318 | 4.56E-06 | 3170 | RikenellaceaeRC9gutgroup | 0.1292 | 0.006458296 | 20.5928773 |
| 4 | 163830516 | rs12501673 | G | A | 0.116395371 | 0.026225344 | 6.29E-06 | 3334 | RikenellaceaeRC9gutgroup | 0.325 | 0.005873609 | 19.68649666 |
| 4 | 155865671 | rs17032291 | C | T | -0.169578595 | 0.036825126 | 6.61E-06 | 3206 | RikenellaceaeRC9gutgroup | 0.1412 | 0.006570931 | 21.19251897 |
| 19 | 1970021 | rs2074881 | C | T | -0.142235956 | 0.032390968 | 9.45E-06 | 3315 | RikenellaceaeRC9gutgroup | 0.1789 | 0.005783204 | 19.27120318 |
| 5 | 31406285 | rs7712231 | G | A | 0.156283835 | 0.035046199 | 7.97E-06 | 3334 | RikenellaceaeRC9gutgroup | 0.165 | 0.005929226 | 19.87401751 |
| 10 | 17019559 | rs61841503 | A | G | 0.092887978 | 0.017145406 | 4.00E-08 | 13568 | Romboutsia | 0.1372 | 0.002158585 | 29.34671251 |
| 7 | 5319387 | rs10279978 | G | A | -0.062220227 | 0.012767535 | 1.17E-06 | 14143 | Romboutsia | 0.3479 | 0.001676405 | 23.74584769 |
| 1 | 171903885 | rs16843578 | T | C | -0.087527594 | 0.019677862 | 5.08E-06 | 14186 | Romboutsia | 0.0825 | 0.001392736 | 19.78212483 |
| 13 | 88725540 | rs77702691 | G | A | -0.094405983 | 0.020854443 | 7.37E-06 | 12487 | Romboutsia | 0.1024 | 0.001638444 | 20.48954267 |
| 2 | 16066705 | rs75987356 | A | G | -0.129578364 | 0.028045444 | 6.71E-06 | 10466 | Romboutsia | 0.0686 | 0.002035517 | 21.34309244 |
| 8 | 5088054 | rs75200530 | G | T | -0.190644379 | 0.042106493 | 5.07E-06 | 5246 | Romboutsia | 0.0467 | 0.003892493 | 20.49199618 |
| 13 | 32720756 | rs9567264 | T | C | 0.058001807 | 0.01274947 | 5.76E-06 | 14180 | Romboutsia | 0.3201 | 0.001457435 | 20.69367051 |
| 7 | 78136725 | rs34302036 | G | A | 0.055026128 | 0.012083475 | 5.88E-06 | 14185 | Romboutsia | 0.4145 | 0.00145979 | 20.734471 |
| 11 | 133834657 | rs7109293 | G | A | 0.091911164 | 0.020573834 | 6.98E-06 | 14141 | Romboutsia | 0.0924 | 0.001409332 | 19.95467401 |
| 5 | 57229635 | rs114398731 | C | G | -0.13103857 | 0.029381546 | 7.96E-06 | 10326 | Romboutsia | 0.0358 | 0.001922565 | 19.88679894 |
| 7 | 106406824 | rs28603357 | C | T | -0.214910996 | 0.047473928 | 8.52E-06 | 3693 | Romboutsia | 0.0636 | 0.005518541 | 20.48196726 |
| 11 | 99665050 | rs11221428 | C | T | -0.072677936 | 0.015826331 | 6.49E-06 | 14187 | Romboutsia | 0.1551 | 0.001484257 | 21.08547822 |
| 6 | 135411737 | rs9389266 | G | T | 0.072281496 | 0.016235341 | 9.38E-06 | 14187 | Romboutsia | 0.169 | 0.001395194 | 19.81847444 |
| 7 | 145760298 | rs62504452 | G | A | -0.071004771 | 0.015665426 | 4.66E-06 | 14178 | Romboutsia | 0.166 | 0.001446928 | 20.54136965 |
| 16 | 8307948 | rs2034589 | C | G | 0.062976455 | 0.012348898 | 5.01E-07 | 17846 | Roseburia | 0.2276 | 0.001455213 | 26.00466803 |
| 4 | 119349 | rs116270582 | A | T | -0.153843918 | 0.032932399 | 1.20E-06 | 9099 | Roseburia | 0.0487 | 0.002392654 | 21.81817628 |
| 12 | 50650057 | rs2160994 | C | T | 0.055068659 | 0.011248106 | 9.70E-07 | 17444 | Roseburia | 0.3797 | 0.00137217 | 23.96626646 |
| 11 | 12009569 | rs16910295 | C | T | -0.098043368 | 0.020956968 | 2.91E-06 | 16648 | Roseburia | 0.0606 | 0.001312946 | 21.88403367 |
| 1 | 95178974 | rs12740451 | C | T | 0.069752736 | 0.015360737 | 7.34E-06 | 17837 | Roseburia | 0.1252 | 0.001154715 | 20.61814952 |
| 3 | 57116228 | rs6445851 | A | G | -0.04973359 | 0.010815826 | 3.53E-06 | 17851 | Roseburia | 0.4006 | 0.001183051 | 21.14129623 |
| 13 | 103117486 | rs9300744 | T | C | -0.058845175 | 0.012622789 | 4.75E-06 | 17854 | Roseburia | 0.2386 | 0.001215758 | 21.73013311 |
| 5 | 89598914 | rs2943022 | C | T | 0.049378559 | 0.010676336 | 4.11E-06 | 17854 | Roseburia | 0.4602 | 0.001196677 | 21.38866661 |
| 6 | 12774611 | rs6930661 | T | C | -0.096158735 | 0.020497475 | 2.48E-06 | 16945 | Roseburia | 0.0755 | 0.001297094 | 22.0052085 |
| 10 | 16051097 | rs4748237 | C | G | 0.048832628 | 0.010640433 | 4.67E-06 | 17851 | Roseburia | 0.4046 | 0.001178493 | 21.05973722 |
| 1 | 145655256 | rs147990086 | G | A | -0.057893629 | 0.013239999 | 8.93E-06 | 17840 | Roseburia | 0.1909 | 0.001070596 | 19.11775899 |
| 5 | 125076494 | rs329182 | C | T | 0.069032724 | 0.015288408 | 5.90E-06 | 17854 | Roseburia | 0.1571 | 0.001140655 | 20.3862259 |
| 1 | 194183650 | rs302266 | C | T | -0.077731153 | 0.01729852 | 8.13E-06 | 17056 | Roseburia | 0.1203 | 0.001182446 | 20.18930584 |
| 6 | 166170836 | rs75326254 | T | C | -0.104627797 | 0.023089702 | 7.50E-06 | 15893 | Roseburia | 0.0636 | 0.001290301 | 20.53065778 |
| 15 | 86512809 | rs55858165 | C | A | 0.17928439 | 0.040495413 | 9.99E-06 | 5447 | Roseburia | 0.0775 | 0.003585551 | 19.59357979 |
| 15 | 37859065 | rs57466170 | T | C | 0.074140517 | 0.017160095 | 8.30E-06 | 17333 | Roseburia | 0.0865 | 0.001075798 | 18.66473095 |
| 5 | 144135655 | rs78753150 | C | A | 0.096873855 | 0.021406676 | 9.98E-06 | 16303 | Roseburia | 0.0586 | 0.00125459 | 20.47676815 |
| 21 | 19151940 | rs243585 | G | C | -0.058593933 | 0.012101692 | 1.33E-06 | 16906 | Ruminiclostridium5 | 0.3012 | 0.001384747 | 23.44022091 |
| 12 | 1891043 | rs2286384 | C | G | -0.051881398 | 0.010748225 | 1.44E-06 | 17394 | Ruminiclostridium5 | 0.501 | 0.001337732 | 23.29699579 |
| 20 | 41177956 | rs79968837 | G | A | -0.095031084 | 0.019350746 | 1.15E-06 | 16602 | Ruminiclostridium5 | 0.0686 | 0.001450591 | 24.11479667 |
| 10 | 12835085 | rs2482038 | A | C | 0.051899121 | 0.010877683 | 1.70E-06 | 17396 | Ruminiclostridium5 | 0.4384 | 0.001306863 | 22.76131285 |
| 5 | 32477664 | rs113753996 | C | T | 0.082068892 | 0.017446254 | 3.99E-06 | 16680 | Ruminiclostridium5 | 0.1372 | 0.001324893 | 22.1258865 |
| 6 | 50405920 | rs1492620 | C | T | -0.083050484 | 0.018007297 | 3.53E-06 | 17397 | Ruminiclostridium5 | 0.0805 | 0.001221187 | 21.26852427 |
| 8 | 131316030 | rs2791343 | C | T | 0.051717025 | 0.011336879 | 5.54E-06 | 17392 | Ruminiclostridium5 | 0.34 | 0.001195119 | 20.80798708 |
| 10 | 35262588 | rs10827477 | G | A | -0.054738653 | 0.011516344 | 2.19E-06 | 17391 | Ruminiclostridium5 | 0.3151 | 0.00129739 | 22.58962536 |
| 20 | 60293505 | rs6121460 | A | G | 0.09329552 | 0.019920597 | 2.64E-06 | 17318 | Ruminiclostridium5 | 0.0805 | 0.001264939 | 21.93141867 |
| 1 | 41878680 | rs2801960 | G | C | 0.052097504 | 0.011511124 | 6.21E-06 | 17387 | Ruminiclostridium5 | 0.3181 | 0.001176691 | 20.48087019 |
| 16 | 86769909 | rs8053158 | G | A | -0.074054329 | 0.015915101 | 5.90E-06 | 17397 | Ruminiclostridium5 | 0.1203 | 0.00124299 | 21.64871671 |
| 13 | 108775162 | rs1223978 | C | T | 0.048416888 | 0.010825421 | 8.16E-06 | 17381 | Ruminiclostridium5 | 0.4801 | 0.001149555 | 20.0011127 |
| 2 | 103755970 | rs73002572 | C | G | 0.181547526 | 0.041407845 | 8.82E-06 | 3761 | Ruminiclostridium5 | 0.0547 | 0.005085083 | 19.21252341 |
| 21 | 33811886 | rs2833828 | A | G | 0.048961687 | 0.010870209 | 6.82E-06 | 17397 | Ruminiclostridium5 | 0.4533 | 0.001164814 | 20.28556174 |
| 3 | 55243531 | rs4955951 | G | A | -0.07137494 | 0.01658264 | 9.96E-06 | 17397 | Ruminiclostridium5 | 0.0924 | 0.00106377 | 18.52397614 |
| 14 | 20954661 | rs1756364 | T | G | 0.099918929 | 0.019716857 | 2.54E-07 | 12933 | Ruminiclostridium6 | 0.1213 | 0.001981798 | 25.67751634 |
| 14 | 56938952 | rs71414120 | G | T | 0.200894087 | 0.040643398 | 1.08E-06 | 5567 | Ruminiclostridium6 | 0.0765 | 0.004369495 | 24.42295693 |
| 16 | 72136154 | rs61060922 | G | T | 0.159129692 | 0.032228344 | 1.09E-06 | 8955 | Ruminiclostridium6 | 0.0447 | 0.002715064 | 24.374149 |
| 1 | 240503826 | rs79968172 | A | G | 0.116139597 | 0.02430834 | 1.66E-06 | 12802 | Ruminiclostridium6 | 0.0557 | 0.001779912 | 22.82349631 |
| 11 | 40289063 | rs77193512 | G | A | 0.073653265 | 0.015316349 | 1.30E-06 | 13380 | Ruminiclostridium6 | 0.167 | 0.001725311 | 23.12110609 |
| 8 | 79766499 | rs11992182 | C | A | 0.06252993 | 0.013787562 | 4.65E-06 | 13381 | Ruminiclostridium6 | 0.2883 | 0.001534777 | 20.56534606 |
| 18 | 76018244 | rs72991535 | G | T | 0.135603894 | 0.029517443 | 4.95E-06 | 9642 | Ruminiclostridium6 | 0.0596 | 0.002184088 | 21.10069575 |
| 11 | 86179076 | rs663262 | C | T | -0.134980385 | 0.03107145 | 3.39E-06 | 7876 | Ruminiclostridium6 | 0.0517 | 0.002390414 | 18.86722321 |
| 13 | 111703249 | rs9555756 | C | A | -0.080413824 | 0.017669194 | 7.10E-06 | 13369 | Ruminiclostridium6 | 0.1243 | 0.00154688 | 20.70918397 |
| 2 | 5548605 | rs792058 | A | G | 0.055423396 | 0.012542318 | 8.58E-06 | 12933 | Ruminiclostridium6 | 0.4274 | 0.001507565 | 19.52376093 |
| 10 | 132651293 | rs10829821 | C | T | -0.097605954 | 0.021607241 | 3.47E-06 | 12802 | Ruminiclostridium6 | 0.0855 | 0.001591419 | 20.40262978 |
| 19 | 10115515 | rs67479537 | C | T | 0.118994191 | 0.026487011 | 9.30E-06 | 11613 | Ruminiclostridium6 | 0.0795 | 0.00173495 | 20.1795187 |
| 15 | 61047117 | rs1871858 | G | C | -0.105421541 | 0.023727442 | 9.12E-06 | 12818 | Ruminiclostridium6 | 0.0795 | 0.001537688 | 19.73736157 |
| 10 | 128214403 | rs116969552 | G | A | -0.166822503 | 0.037668056 | 9.16E-06 | 6824 | Ruminiclostridium6 | 0.0358 | 0.002866008 | 19.60810183 |
| 19 | 49209339 | rs2548459 | T | C | 0.055486587 | 0.012275456 | 6.40E-06 | 13377 | Ruminiclostridium6 | 0.4702 | 0.001525033 | 20.42847002 |
| 7 | 101271282 | rs73176030 | C | T | 0.058665044 | 0.013207973 | 7.29E-06 | 13378 | Ruminiclostridium6 | 0.3181 | 0.0014725 | 19.72520555 |
| 4 | 36478011 | rs35362464 | A | C | 0.072007723 | 0.016538673 | 8.99E-06 | 13308 | Ruminiclostridium6 | 0.1511 | 0.001422414 | 18.95359416 |
| 3 | 157841287 | rs57665991 | G | C | -0.064150707 | 0.012333861 | 2.07E-07 | 16725 | Ruminiclostridium9 | 0.2386 | 0.001614867 | 27.04910063 |
| 1 | 247710285 | rs12040548 | T | G | 0.057042151 | 0.012235891 | 3.15E-06 | 16721 | Ruminiclostridium9 | 0.3111 | 0.001298058 | 21.73044481 |
| 7 | 47013498 | rs115044523 | A | G | -0.098002558 | 0.020261641 | 2.37E-06 | 16107 | Ruminiclostridium9 | 0.0915 | 0.001450376 | 23.39223203 |
| 11 | 68886460 | rs12419854 | A | T | -0.072797471 | 0.015559424 | 3.18E-06 | 16027 | Ruminiclostridium9 | 0.1779 | 0.001363957 | 21.88725664 |
| 20 | 2210530 | rs6082461 | C | A | 0.058642458 | 0.013100394 | 4.87E-06 | 16650 | Ruminiclostridium9 | 0.2068 | 0.00120204 | 20.03565039 |
| 9 | 74214419 | rs113048721 | G | C | 0.060073481 | 0.013273731 | 4.10E-06 | 16719 | Ruminiclostridium9 | 0.2386 | 0.001223595 | 20.47989109 |
| 19 | 34371198 | rs918449 | G | A | -0.095090817 | 0.019717682 | 2.56E-06 | 16725 | Ruminiclostridium9 | 0.0696 | 0.00138866 | 23.25484764 |
| 6 | 128568828 | rs73592673 | T | A | -0.08162902 | 0.016951466 | 2.14E-06 | 15493 | Ruminiclostridium9 | 0.1471 | 0.001494478 | 23.18561346 |
| 7 | 155701010 | rs79082720 | G | C | 0.092857633 | 0.020487143 | 6.47E-06 | 16094 | Ruminiclostridium9 | 0.0954 | 0.001274836 | 20.54085088 |
| 8 | 14697544 | rs74303178 | C | T | 0.053274162 | 0.011927361 | 7.92E-06 | 16706 | Ruminiclostridium9 | 0.3201 | 0.001192762 | 19.9476862 |
| 2 | 204706865 | rs13033315 | A | T | 0.051119464 | 0.011141773 | 5.68E-06 | 16724 | Ruminiclostridium9 | 0.4503 | 0.001257122 | 21.0480571 |
| 3 | 29179983 | rs9809789 | T | C | -0.071745357 | 0.015967315 | 8.72E-06 | 16025 | Ruminiclostridium9 | 0.168 | 0.001258285 | 20.18689962 |
| 13 | 90441578 | rs9522712 | C | T | 0.069972768 | 0.015493905 | 4.66E-06 | 16725 | Ruminiclostridium9 | 0.1451 | 0.001217982 | 20.39315377 |
| 6 | 67655320 | rs78191726 | C | T | 0.094499761 | 0.02102058 | 7.58E-06 | 16039 | Ruminiclostridium9 | 0.0905 | 0.001258484 | 20.20774529 |
| 12 | 10702414 | rs7137760 | T | C | 0.050797017 | 0.011218236 | 7.07E-06 | 16722 | Ruminiclostridium9 | 0.4145 | 0.001224636 | 20.50101193 |
| 22 | 17793012 | rs5994253 | G | A | -0.081130217 | 0.015762089 | 2.35E-07 | 15288 | RuminococcaceaeNK4A214group | 0.1461 | 0.001729956 | 26.48993606 |
| 22 | 49796014 | rs136761 | A | G | -0.058752097 | 0.01191547 | 8.15E-07 | 14822 | RuminococcaceaeNK4A214group | 0.4294 | 0.001637592 | 24.30892738 |
| 1 | 157338888 | rs11586410 | A | G | -0.086342178 | 0.016993683 | 3.66E-07 | 14822 | RuminococcaceaeNK4A214group | 0.1342 | 0.001738635 | 25.81144823 |
| 12 | 78373555 | rs34576931 | C | G | -0.087331196 | 0.019488596 | 4.72E-06 | 14678 | RuminococcaceaeNK4A214group | 0.0915 | 0.001366209 | 20.07791047 |
| 2 | 235430299 | rs114244418 | G | C | -0.17526622 | 0.037277927 | 3.59E-06 | 7123 | RuminococcaceaeNK4A214group | 0.0408 | 0.003093744 | 22.09891647 |
| 20 | 18030357 | rs4814689 | T | C | -0.108300291 | 0.023070832 | 4.55E-06 | 14009 | RuminococcaceaeNK4A214group | 0.0666 | 0.001570518 | 22.03285185 |
| 7 | 132042135 | rs73158814 | G | C | -0.10925732 | 0.022745845 | 2.20E-06 | 13077 | RuminococcaceaeNK4A214group | 0.0736 | 0.001761259 | 23.06909352 |
| 2 | 239087912 | rs12731 | G | A | -0.052768427 | 0.01150534 | 4.87E-06 | 15288 | RuminococcaceaeNK4A214group | 0.4513 | 0.001374047 | 21.03257942 |
| 2 | 141897042 | rs7573569 | C | T | 0.107738536 | 0.023363816 | 3.23E-06 | 14822 | RuminococcaceaeNK4A214group | 0.0716 | 0.001432601 | 21.26160089 |
| 3 | 26509287 | rs147475196 | G | A | -0.133768553 | 0.029519585 | 4.72E-06 | 9917 | RuminococcaceaeNK4A214group | 0.0775 | 0.002066374 | 20.53052082 |
| 16 | 24073806 | rs62027366 | C | T | 0.061538935 | 0.013761184 | 6.58E-06 | 14822 | RuminococcaceaeNK4A214group | 0.2296 | 0.001347398 | 19.99538251 |
| 4 | 159932022 | rs12642039 | C | T | -0.055300297 | 0.011939746 | 3.43E-06 | 15276 | RuminococcaceaeNK4A214group | 0.3658 | 0.001402315 | 21.44904293 |
| 5 | 35288938 | rs35559912 | C | T | -0.092507085 | 0.020367383 | 4.89E-06 | 13976 | RuminococcaceaeNK4A214group | 0.0656 | 0.00147386 | 20.62611295 |
| 3 | 84725039 | rs13087692 | G | T | 0.057462316 | 0.012593957 | 8.69E-06 | 15288 | RuminococcaceaeNK4A214group | 0.3052 | 0.001359879 | 20.81541221 |
| 1 | 100248591 | rs6681678 | T | C | -0.100174165 | 0.024000023 | 9.05E-06 | 13031 | RuminococcaceaeNK4A214group | 0.0497 | 0.00133515 | 17.41893007 |
| 5 | 123846318 | rs11241747 | T | C | 0.053385843 | 0.012003462 | 6.59E-06 | 14822 | RuminococcaceaeNK4A214group | 0.3907 | 0.001332764 | 19.77791644 |
| 15 | 80713184 | rs77564310 | C | A | -0.071309265 | 0.014085525 | 3.29E-07 | 17000 | RuminococcaceaeUCG002 | 0.1789 | 0.001505368 | 25.62682101 |
| 12 | 47384118 | rs55793120 | C | T | 0.137396059 | 0.027413962 | 4.81E-07 | 11669 | RuminococcaceaeUCG002 | 0.0507 | 0.002148015 | 25.11484381 |
| 1 | 14732458 | rs10927423 | A | C | -0.071361729 | 0.014771224 | 8.50E-07 | 17096 | RuminococcaceaeUCG002 | 0.1829 | 0.001363361 | 23.33710324 |
| 4 | 12207874 | rs67746927 | G | C | -0.054237005 | 0.011035718 | 9.17E-07 | 16651 | RuminococcaceaeUCG002 | 0.4414 | 0.001448506 | 24.15116348 |
| 11 | 111712942 | rs116974815 | A | C | -0.189730541 | 0.039656625 | 2.03E-06 | 5819 | RuminococcaceaeUCG002 | 0.0577 | 0.003918227 | 22.88198499 |
| 14 | 77502546 | rs7155595 | A | C | 0.056992918 | 0.011698636 | 1.15E-06 | 17084 | RuminococcaceaeUCG002 | 0.3091 | 0.001387327 | 23.73124331 |
| 11 | 86335459 | rs7120052 | C | A | 0.062479604 | 0.013552314 | 1.97E-06 | 17097 | RuminococcaceaeUCG002 | 0.1759 | 0.001241624 | 21.25195166 |
| 1 | 227563126 | rs10916131 | T | C | -0.069333027 | 0.014675325 | 2.87E-06 | 17097 | RuminococcaceaeUCG002 | 0.1382 | 0.001303822 | 22.31793668 |
| 1 | 238938497 | rs79016051 | T | C | -0.088774672 | 0.018942419 | 2.34E-06 | 16937 | RuminococcaceaeUCG002 | 0.0905 | 0.001295114 | 21.96119729 |
| 19 | 54475808 | rs12463378 | G | A | -0.052206066 | 0.011208649 | 2.96E-06 | 16994 | RuminococcaceaeUCG002 | 0.4175 | 0.001274928 | 21.69123493 |
| 5 | 123822114 | rs11750293 | T | G | -0.057830385 | 0.012051229 | 1.76E-06 | 17094 | RuminococcaceaeUCG002 | 0.2813 | 0.001345306 | 23.0249482 |
| 1 | 190171640 | rs2265670 | G | C | -0.051240397 | 0.010928696 | 2.99E-06 | 17096 | RuminococcaceaeUCG002 | 0.4583 | 0.001284209 | 21.9804934 |
| 10 | 73820548 | rs15256 | T | C | 0.073237587 | 0.016833997 | 9.46E-06 | 17095 | RuminococcaceaeUCG002 | 0.0865 | 0.001105971 | 18.92529999 |
| 4 | 23325040 | rs882348 | G | A | -0.079994585 | 0.017861803 | 5.45E-06 | 17097 | RuminococcaceaeUCG002 | 0.1064 | 0.001171768 | 20.05486802 |
| 2 | 45178830 | rs72874194 | C | G | -0.077028853 | 0.016724954 | 3.47E-06 | 16651 | RuminococcaceaeUCG002 | 0.0994 | 0.001272283 | 21.20922721 |
| 3 | 24045453 | rs6793778 | T | C | -0.055870572 | 0.012525794 | 9.81E-06 | 17094 | RuminococcaceaeUCG002 | 0.2624 | 0.001162537 | 19.89321187 |
| 12 | 129451330 | rs7342369 | A | C | -0.052780292 | 0.011602332 | 5.66E-06 | 16651 | RuminococcaceaeUCG002 | 0.3231 | 0.00124129 | 20.69192357 |
| 9 | 20131746 | rs10964441 | A | G | -0.149060086 | 0.034485997 | 7.45E-06 | 5346 | RuminococcaceaeUCG002 | 0.0527 | 0.003482516 | 18.67560407 |
| 11 | 43344751 | rs11607472 | G | A | -0.0780229 | 0.017632484 | 7.19E-06 | 16282 | RuminococcaceaeUCG002 | 0.1074 | 0.001201123 | 19.57779808 |
| 19 | 43637890 | rs7249614 | G | A | -0.049277826 | 0.011080679 | 9.07E-06 | 16651 | RuminococcaceaeUCG002 | 0.3847 | 0.001186355 | 19.77507785 |
| 5 | 141014951 | rs76847269 | G | A | 0.163508017 | 0.035615077 | 5.17E-06 | 7654 | RuminococcaceaeUCG002 | 0.0567 | 0.002746171 | 21.07156643 |
| 2 | 120757853 | rs6542556 | G | A | 0.050974014 | 0.011406021 | 7.86E-06 | 17094 | RuminococcaceaeUCG002 | 0.3449 | 0.001167021 | 19.97001989 |
| 13 | 87920450 | rs57079348 | G | T | -0.07657295 | 0.01728195 | 7.22E-06 | 16372 | RuminococcaceaeUCG002 | 0.1213 | 0.001197686 | 19.62963529 |
| 15 | 90780618 | rs56030423 | A | G | -0.098287388 | 0.021634426 | 6.30E-06 | 14937 | RuminococcaceaeUCG002 | 0.0885 | 0.001379881 | 20.63700563 |
| 14 | 73589896 | rs362417 | C | G | -0.054862993 | 0.01209839 | 7.80E-06 | 17097 | RuminococcaceaeUCG002 | 0.2664 | 0.001201329 | 20.56141497 |
| 9 | 114066670 | rs113147300 | G | A | -0.075841986 | 0.016456212 | 7.69E-06 | 16370 | RuminococcaceaeUCG002 | 0.1322 | 0.001295829 | 21.23765652 |
| 7 | 51541468 | rs73341549 | C | T | -0.169857156 | 0.031876785 | 1.51E-07 | 6744 | RuminococcaceaeUCG003 | 0.0507 | 0.004192533 | 28.3850621 |
| 2 | 205238716 | rs6759615 | G | A | 0.10252284 | 0.020020394 | 7.86E-07 | 14410 | RuminococcaceaeUCG003 | 0.0696 | 0.001816529 | 26.22018414 |
| 19 | 49209851 | rs646327 | A | G | 0.058669624 | 0.01183695 | 7.83E-07 | 14406 | RuminococcaceaeUCG003 | 0.4682 | 0.001702408 | 24.56330036 |
| 12 | 75496463 | rs11613919 | T | G | 0.072760434 | 0.015571648 | 1.63E-06 | 14309 | RuminococcaceaeUCG003 | 0.2127 | 0.001523526 | 21.83034917 |
| 9 | 134416970 | rs11243416 | C | T | -0.092565265 | 0.019120311 | 1.67E-06 | 14004 | RuminococcaceaeUCG003 | 0.1014 | 0.001670813 | 23.43387875 |
| 15 | 35071718 | rs16959793 | C | A | -0.062527033 | 0.013125865 | 2.22E-06 | 14412 | RuminococcaceaeUCG003 | 0.2803 | 0.001572071 | 22.68921699 |
| 8 | 82026852 | rs4452755 | C | A | -0.06344632 | 0.01347408 | 3.29E-06 | 14406 | RuminococcaceaeUCG003 | 0.3052 | 0.00153675 | 22.16942056 |
| 2 | 37905976 | rs10490280 | T | C | -0.067209365 | 0.014330937 | 4.16E-06 | 14004 | RuminococcaceaeUCG003 | 0.2177 | 0.001568111 | 21.99117068 |
| 6 | 105781538 | rs4532474 | A | G | 0.076923106 | 0.017044961 | 4.82E-06 | 14412 | RuminococcaceaeUCG003 | 0.1491 | 0.001411185 | 20.36391657 |
| 18 | 46485154 | rs4629039 | A | T | 0.055374213 | 0.012265944 | 6.54E-06 | 14404 | RuminococcaceaeUCG003 | 0.34 | 0.001412915 | 20.37759159 |
| 7 | 97348436 | rs2523124 | C | T | -0.054662483 | 0.012082341 | 5.78E-06 | 14409 | RuminococcaceaeUCG003 | 0.3976 | 0.00141849 | 20.46521281 |
| 1 | 13794594 | rs3013089 | A | G | -0.05514734 | 0.012034599 | 4.38E-06 | 14408 | RuminococcaceaeUCG003 | 0.3847 | 0.001455291 | 20.9954708 |
| 3 | 41982393 | rs78720113 | G | A | -0.115348392 | 0.02496006 | 7.59E-06 | 13078 | RuminococcaceaeUCG003 | 0.0696 | 0.001630354 | 21.35332011 |
| 22 | 25292719 | rs139730 | C | G | -0.057765528 | 0.013064482 | 9.72E-06 | 14004 | RuminococcaceaeUCG003 | 0.2863 | 0.001394104 | 19.54749253 |
| 3 | 54427795 | rs6769553 | G | A | 0.084974255 | 0.015745058 | 7.91E-08 | 9501 | RuminococcaceaeUCG004 | 0.2843 | 0.003056241 | 29.12022833 |
| 1 | 103732214 | rs12125734 | T | G | 0.133971622 | 0.02574165 | 2.09E-07 | 8938 | RuminococcaceaeUCG004 | 0.0865 | 0.003021331 | 27.08043711 |
| 3 | 169966722 | rs511258 | A | G | -0.075747368 | 0.016247466 | 4.52E-06 | 9500 | RuminococcaceaeUCG004 | 0.2634 | 0.002282694 | 21.7306328 |
| 7 | 24791998 | rs2248146 | C | T | 0.068945165 | 0.015364919 | 8.20E-06 | 9502 | RuminococcaceaeUCG004 | 0.3221 | 0.002114525 | 20.13055055 |
| 9 | 7317307 | rs10976229 | G | T | 0.095975994 | 0.021455386 | 7.04E-06 | 9116 | RuminococcaceaeUCG004 | 0.1412 | 0.002190262 | 20.0058625 |
| 2 | 239846337 | rs7569771 | G | A | -0.075914029 | 0.01703081 | 8.12E-06 | 9502 | RuminococcaceaeUCG004 | 0.2167 | 0.002086658 | 19.86470126 |
| 8 | 139253064 | rs872501 | A | G | 0.116124682 | 0.025946001 | 5.81E-06 | 8473 | RuminococcaceaeUCG004 | 0.1272 | 0.002358552 | 20.02652996 |
| 1 | 18676399 | rs550351 | C | A | 0.078587023 | 0.018003041 | 9.43E-06 | 9498 | RuminococcaceaeUCG004 | 0.4404 | 0.0020022 | 19.05103147 |
| 6 | 2119329 | rs3800154 | C | A | -0.079795901 | 0.017763932 | 6.12E-06 | 9406 | RuminococcaceaeUCG004 | 0.2207 | 0.002140658 | 20.17393407 |
| 6 | 132887921 | rs11961899 | A | G | -0.070782539 | 0.01613814 | 9.18E-06 | 9501 | RuminococcaceaeUCG004 | 0.2714 | 0.00202068 | 19.23330392 |
| 3 | 197683751 | rs9818949 | T | G | 0.085992665 | 0.018867146 | 5.39E-06 | 9116 | RuminococcaceaeUCG004 | 0.2187 | 0.002273618 | 20.76897712 |
| 11 | 15771251 | rs7123615 | G | C | -0.07861572 | 0.017959154 | 7.09E-06 | 9502 | RuminococcaceaeUCG004 | 0.169 | 0.002012598 | 19.15824014 |
| 7 | 18026604 | rs10950694 | C | T | 0.057762846 | 0.011412014 | 4.30E-07 | 15821 | RuminococcaceaeUCG005 | 0.4235 | 0.001616724 | 25.61637395 |
| 20 | 16312851 | rs34781347 | A | G | 0.188684782 | 0.03864836 | 6.05E-07 | 6061 | RuminococcaceaeUCG005 | 0.0596 | 0.003917083 | 23.82694012 |
| 11 | 27747671 | rs12288512 | G | A | 0.066635035 | 0.014435565 | 3.10E-06 | 15473 | RuminococcaceaeUCG005 | 0.1799 | 0.001375199 | 21.30500165 |
| 5 | 32755085 | rs60081663 | G | C | 0.158081538 | 0.031958601 | 9.28E-07 | 9342 | RuminococcaceaeUCG005 | 0.0527 | 0.002612227 | 24.46210315 |
| 2 | 198122357 | rs114279581 | G | A | -0.146604207 | 0.031603425 | 3.22E-06 | 8465 | RuminococcaceaeUCG005 | 0.0477 | 0.002535683 | 21.5140387 |
| 18 | 22575871 | rs12458218 | C | T | 0.067732182 | 0.014452491 | 2.41E-06 | 16099 | RuminococcaceaeUCG005 | 0.1769 | 0.001362429 | 21.96093295 |
| 8 | 3099727 | rs35166120 | G | C | -0.068615325 | 0.014625203 | 3.75E-06 | 16092 | RuminococcaceaeUCG005 | 0.1849 | 0.00136595 | 22.00819665 |
| 1 | 187906034 | rs7555878 | G | A | 0.058667737 | 0.012527736 | 2.81E-06 | 16100 | RuminococcaceaeUCG005 | 0.2803 | 0.001360305 | 21.92802444 |
| 17 | 7034575 | rs394449 | T | A | 0.069290871 | 0.014871206 | 2.60E-06 | 15728 | RuminococcaceaeUCG005 | 0.1789 | 0.001378438 | 21.70723241 |
| 14 | 94630731 | rs10873449 | C | T | 0.06548432 | 0.01439735 | 4.11E-06 | 16098 | RuminococcaceaeUCG005 | 0.169 | 0.001283453 | 20.68500286 |
| 10 | 62662781 | rs2893871 | A | G | -0.073643982 | 0.015547986 | 3.54E-06 | 16103 | RuminococcaceaeUCG005 | 0.1521 | 0.001391281 | 22.43222308 |
| 10 | 4240906 | rs72776570 | A | C | 0.087067007 | 0.019717899 | 5.36E-06 | 15376 | RuminococcaceaeUCG005 | 0.0825 | 0.001266462 | 19.49527898 |
| 15 | 68705697 | rs898577 | C | T | -0.12301218 | 0.028667054 | 7.46E-06 | 9991 | RuminococcaceaeUCG005 | 0.0547 | 0.001839592 | 18.40954926 |
| 5 | 154003091 | rs7449320 | A | C | 0.059916334 | 0.013083635 | 4.81E-06 | 16095 | RuminococcaceaeUCG005 | 0.2207 | 0.001301299 | 20.96909279 |
| 4 | 7302344 | rs10937802 | A | G | 0.075581108 | 0.016822622 | 8.17E-06 | 15821 | RuminococcaceaeUCG005 | 0.0974 | 0.001274241 | 20.18293109 |
| 2 | 239509030 | rs7586445 | A | G | 0.07823492 | 0.017645763 | 8.81E-06 | 15099 | RuminococcaceaeUCG005 | 0.1262 | 0.001300191 | 19.65453886 |
| 12 | 47384118 | rs55793120 | C | T | 0.121547709 | 0.0279546 | 7.37E-06 | 11224 | RuminococcaceaeUCG005 | 0.0507 | 0.001681545 | 18.90207971 |
| 14 | 39306809 | rs8009993 | C | G | -0.135936179 | 0.024485659 | 4.42E-08 | 7389 | RuminococcaceaeUCG009 | 0.1292 | 0.004153872 | 30.81264241 |
| 17 | 32786955 | rs1550196 | A | G | 0.130841508 | 0.026247787 | 1.13E-06 | 7389 | RuminococcaceaeUCG009 | 0.1173 | 0.003351676 | 24.84209035 |
| 6 | 78106701 | rs4708333 | G | T | -0.084032963 | 0.017475702 | 1.56E-06 | 7511 | RuminococcaceaeUCG009 | 0.3022 | 0.003069004 | 23.11609389 |
| 1 | 39983022 | rs61779334 | C | G | -0.138137216 | 0.029207242 | 1.94E-06 | 6865 | RuminococcaceaeUCG009 | 0.1044 | 0.003247783 | 22.36216023 |
| 1 | 202299085 | rs4079028 | T | C | 0.091562303 | 0.019936034 | 3.28E-06 | 7480 | RuminococcaceaeUCG009 | 0.1938 | 0.002812103 | 21.08821208 |
| 12 | 12970792 | rs2058609 | G | A | 0.081646419 | 0.017471971 | 3.12E-06 | 7510 | RuminococcaceaeUCG009 | 0.3141 | 0.002899276 | 21.83105814 |
| 4 | 7426937 | rs12508214 | T | C | -0.077464871 | 0.016893699 | 4.75E-06 | 7511 | RuminococcaceaeUCG009 | 0.3509 | 0.002791569 | 21.02056842 |
| 13 | 106650093 | rs9558661 | C | T | -0.089782261 | 0.020073862 | 7.01E-06 | 7480 | RuminococcaceaeUCG009 | 0.1869 | 0.002667213 | 19.99876071 |
| 16 | 1979831 | rs758191 | G | T | 0.177019514 | 0.037511304 | 9.01E-06 | 5829 | RuminococcaceaeUCG009 | 0.0636 | 0.003805992 | 22.26224337 |
| 2 | 75512668 | rs2192926 | G | A | -0.089048031 | 0.019298901 | 4.88E-06 | 7511 | RuminococcaceaeUCG009 | 0.2366 | 0.002826548 | 21.28471403 |
| 6 | 51803869 | rs113006825 | C | T | -0.092889294 | 0.020803474 | 7.98E-06 | 7165 | RuminococcaceaeUCG009 | 0.1799 | 0.002774832 | 19.93142533 |
| 2 | 66396776 | rs138460696 | G | A | 0.13927429 | 0.031579435 | 9.81E-06 | 6865 | RuminococcaceaeUCG009 | 0.1064 | 0.002825296 | 19.44494224 |
| 7 | 32001710 | rs6952765 | A | G | 0.073209862 | 0.016677682 | 8.13E-06 | 7511 | RuminococcaceaeUCG009 | 0.4612 | 0.002558923 | 19.26425203 |
| 19 | 52399401 | rs78410648 | G | A | 0.120988897 | 0.027731677 | 9.67E-06 | 7510 | RuminococcaceaeUCG009 | 0.0974 | 0.00252813 | 19.02931192 |
| 15 | 36607072 | rs35506912 | C | G | -0.069374287 | 0.014792554 | 3.21E-06 | 12793 | RuminococcaceaeUCG010 | 0.2495 | 0.001716297 | 21.99089242 |
| 9 | 135968557 | rs682403 | G | A | -0.058816059 | 0.01246713 | 2.37E-06 | 12864 | RuminococcaceaeUCG010 | 0.4761 | 0.001727158 | 22.25314126 |
| 7 | 16349864 | rs6958419 | T | C | -0.058571831 | 0.012499376 | 2.84E-06 | 12870 | RuminococcaceaeUCG010 | 0.4891 | 0.001703264 | 21.95500043 |
| 6 | 104728618 | rs2820282 | C | A | -0.059230421 | 0.012591704 | 2.85E-06 | 12865 | RuminococcaceaeUCG010 | 0.4523 | 0.001716977 | 22.12346275 |
| 16 | 5233941 | rs12597105 | A | G | 0.067085456 | 0.014441482 | 4.87E-06 | 12415 | RuminococcaceaeUCG010 | 0.2773 | 0.001735131 | 21.57561723 |
| 11 | 130676996 | rs7935775 | T | A | -0.063135075 | 0.013795347 | 4.99E-06 | 12864 | RuminococcaceaeUCG010 | 0.3002 | 0.001625525 | 20.94154104 |
| 4 | 31057482 | rs73218807 | A | G | -0.166210686 | 0.036793834 | 6.43E-06 | 6714 | RuminococcaceaeUCG010 | 0.0577 | 0.00303018 | 20.40038686 |
| 4 | 40707798 | rs7441445 | T | C | -0.056949974 | 0.012650232 | 6.80E-06 | 12852 | RuminococcaceaeUCG010 | 0.4354 | 0.001574472 | 20.26387297 |
| 6 | 105073676 | rs1416041 | C | A | -0.182339224 | 0.033989816 | 7.04E-08 | 3632 | RuminococcaceaeUCG011 | 0.1342 | 0.007861206 | 28.76228275 |
| 1 | 179370499 | rs12724320 | T | C | -0.120880657 | 0.024922917 | 1.52E-06 | 3632 | RuminococcaceaeUCG011 | 0.2753 | 0.006435262 | 23.51130161 |
| 12 | 29634919 | rs79113084 | T | C | -0.152165548 | 0.031751932 | 2.06E-06 | 3610 | RuminococcaceaeUCG011 | 0.1551 | 0.006321658 | 22.95364651 |
| 1 | 219901055 | rs9729514 | G | A | 0.18493353 | 0.039461636 | 2.37E-06 | 3632 | RuminococcaceaeUCG011 | 0.1024 | 0.00601059 | 21.95037492 |
| 7 | 111763988 | rs2729556 | T | C | -0.109096996 | 0.023369889 | 3.19E-06 | 3632 | RuminococcaceaeUCG011 | 0.499 | 0.005964422 | 21.78076187 |
| 7 | 11222521 | rs10274562 | T | C | 0.11091724 | 0.024455551 | 6.50E-06 | 3632 | RuminococcaceaeUCG011 | 0.3519 | 0.005631766 | 20.55909469 |
| 3 | 76205107 | rs4490371 | C | T | -0.111816319 | 0.024896269 | 7.75E-06 | 3610 | RuminococcaceaeUCG011 | 0.331 | 0.005556671 | 20.16049401 |
| 3 | 185469491 | rs12636310 | A | G | 0.132725381 | 0.028203654 | 2.81E-06 | 3632 | RuminococcaceaeUCG011 | 0.2177 | 0.00606054 | 22.13390243 |
| 10 | 2219930 | rs12781711 | T | C | -0.065612917 | 0.011747657 | 2.55E-08 | 16772 | RuminococcaceaeUCG013 | 0.3131 | 0.001856454 | 31.19064591 |
| 5 | 141395140 | rs12189346 | A | G | 0.068496125 | 0.014558037 | 1.68E-06 | 16470 | RuminococcaceaeUCG013 | 0.163 | 0.001342299 | 22.13469924 |
| 12 | 30245557 | rs75088940 | C | T | -0.094296811 | 0.02007125 | 2.55E-06 | 15638 | RuminococcaceaeUCG013 | 0.0934 | 0.001409456 | 22.06935396 |
| 10 | 19780463 | rs16918863 | C | A | 0.111491182 | 0.024015687 | 4.16E-06 | 12643 | RuminococcaceaeUCG013 | 0.0606 | 0.001701771 | 21.54876025 |
| 3 | 9534657 | rs76973485 | T | G | 0.194975714 | 0.041821342 | 3.35E-06 | 5299 | RuminococcaceaeUCG013 | 0.0368 | 0.004085013 | 21.72706983 |
| 3 | 197049996 | rs12485353 | A | G | -0.060790269 | 0.013084699 | 4.19E-06 | 16771 | RuminococcaceaeUCG013 | 0.1789 | 0.001285355 | 21.58186088 |
| 13 | 76583319 | rs9565219 | A | T | -0.052459177 | 0.011770144 | 8.73E-06 | 16772 | RuminococcaceaeUCG013 | 0.337 | 0.001182988 | 19.86220997 |
| 16 | 10863459 | rs1729063 | C | G | -0.053341105 | 0.01207565 | 9.64E-06 | 16470 | RuminococcaceaeUCG013 | 0.328 | 0.001183301 | 19.50968396 |
| 7 | 32530997 | rs7784330 | A | G | -0.049832949 | 0.011206613 | 8.16E-06 | 16770 | RuminococcaceaeUCG013 | 0.3887 | 0.001177713 | 19.77118204 |
| 1 | 9361576 | rs11581881 | T | C | 0.066121072 | 0.014473649 | 4.73E-06 | 15730 | RuminococcaceaeUCG013 | 0.1809 | 0.00132501 | 20.86740724 |
| 9 | 136620584 | rs2428106 | G | C | -0.0490863 | 0.010990264 | 8.38E-06 | 16768 | RuminococcaceaeUCG013 | 0.4394 | 0.001188247 | 19.94584798 |
| 8 | 40311885 | rs2730183 | A | G | -0.048869155 | 0.010990742 | 8.44E-06 | 16772 | RuminococcaceaeUCG013 | 0.4642 | 0.001177387 | 19.76805032 |
| 5 | 3628882 | rs9313055 | C | T | 0.105087207 | 0.023446005 | 9.55E-06 | 15764 | RuminococcaceaeUCG013 | 0.0686 | 0.001272748 | 20.08662097 |
| 9 | 13609119 | rs12336782 | C | T | -0.085604658 | 0.018931084 | 8.60E-06 | 16772 | RuminococcaceaeUCG013 | 0.0815 | 0.001217671 | 20.44523123 |
| 10 | 115650278 | rs4385846 | T | G | 0.05984045 | 0.013180685 | 6.46E-06 | 16772 | RuminococcaceaeUCG013 | 0.2068 | 0.001227426 | 20.60923836 |
| 5 | 26110626 | rs115777838 | C | T | -0.188349525 | 0.038664295 | 4.62E-07 | 5674 | RuminococcaceaeUCG014 | 0.0537 | 0.004164923 | 23.72224406 |
| 2 | 57205854 | rs72809222 | C | T | 0.067177506 | 0.013983796 | 2.41E-06 | 13922 | RuminococcaceaeUCG014 | 0.2515 | 0.001654919 | 23.07465459 |
| 3 | 101334609 | rs12638134 | G | T | 0.058254795 | 0.011965732 | 1.21E-06 | 14403 | RuminococcaceaeUCG014 | 0.3897 | 0.001642925 | 23.69870024 |
| 9 | 14363769 | rs56105232 | A | G | 0.139275689 | 0.029913177 | 2.91E-06 | 9190 | RuminococcaceaeUCG014 | 0.0726 | 0.002353351 | 21.67359889 |
| 2 | 134854659 | rs995642 | T | C | 0.060047967 | 0.012641687 | 1.90E-06 | 14402 | RuminococcaceaeUCG014 | 0.326 | 0.00156417 | 22.55933073 |
| 5 | 36435597 | rs10941294 | T | C | -0.122057405 | 0.026001669 | 2.40E-06 | 12615 | RuminococcaceaeUCG014 | 0.0686 | 0.001743735 | 22.03215315 |
| 18 | 4084336 | rs79640386 | A | T | -0.110863476 | 0.024809787 | 8.74E-06 | 13692 | RuminococcaceaeUCG014 | 0.0646 | 0.001456234 | 19.9649155 |
| 2 | 119467367 | rs439810 | C | G | -0.057712693 | 0.012667538 | 7.04E-06 | 14403 | RuminococcaceaeUCG014 | 0.2972 | 0.001439063 | 20.75381336 |
| 1 | 237422989 | rs10495392 | T | C | -0.082485954 | 0.018719441 | 9.96E-06 | 13902 | RuminococcaceaeUCG014 | 0.1193 | 0.001394732 | 19.41385491 |
| 7 | 109564034 | rs62478832 | A | T | -0.058128986 | 0.012903031 | 6.04E-06 | 14331 | RuminococcaceaeUCG014 | 0.2883 | 0.0014142 | 20.29277388 |
| 11 | 126024042 | rs61898819 | T | A | 0.060786464 | 0.01387607 | 9.92E-06 | 14325 | RuminococcaceaeUCG014 | 0.2107 | 0.001337842 | 19.18757866 |
| 10 | 119928413 | rs853612 | G | A | -0.05282572 | 0.011936668 | 9.75E-06 | 14403 | RuminococcaceaeUCG014 | 0.4026 | 0.001357943 | 19.58232731 |
| 3 | 123829361 | rs73186226 | A | G | -0.099385194 | 0.02168186 | 6.72E-06 | 13760 | RuminococcaceaeUCG014 | 0.0875 | 0.001524647 | 21.00811923 |
| 8 | 3636548 | rs34402072 | T | C | -0.068802318 | 0.015608285 | 9.80E-06 | 13863 | RuminococcaceaeUCG014 | 0.1918 | 0.001399685 | 19.42822468 |
| 1 | 15322582 | rs74060145 | G | C | -0.115766761 | 0.025419855 | 8.71E-06 | 13011 | RuminococcaceaeUCG014 | 0.0646 | 0.001591546 | 20.73742692 |
| 16 | 87759750 | rs77627087 | G | C | 0.067561994 | 0.015010339 | 7.43E-06 | 14390 | RuminococcaceaeUCG014 | 0.1859 | 0.001405892 | 20.25645931 |
| 19 | 49175964 | rs17296933 | G | C | -0.08295439 | 0.018505763 | 7.34E-06 | 13177 | RuminococcaceaeUCG014 | 0.1272 | 0.001522602 | 20.09087468 |
| 11 | 131659000 | rs10791168 | G | A | -0.066462357 | 0.015006876 | 9.76E-06 | 14403 | RuminococcaceaeUCG014 | 0.1839 | 0.001359962 | 19.61149071 |
| 11 | 45688595 | rs10769159 | C | G | -0.064027674 | 0.011005922 | 5.29E-09 | 16603 | Ruminococcus1 | 0.5338 | 0.002034284 | 33.83999128 |
| 11 | 114023573 | rs7117576 | G | A | 0.082950453 | 0.017089133 | 6.48E-07 | 16606 | Ruminococcus1 | 0.0885 | 0.001416826 | 23.55836442 |
| 17 | 71256018 | rs17781867 | T | C | 0.099927121 | 0.021172816 | 1.96E-06 | 14349 | Ruminococcus1 | 0.0924 | 0.001549938 | 22.27147494 |
| 2 | 10533921 | rs7583465 | T | C | 0.052765423 | 0.011261892 | 2.56E-06 | 16588 | Ruminococcus1 | 0.3767 | 0.001321624 | 21.94945681 |
| 2 | 65925212 | rs78613526 | A | G | 0.167496641 | 0.036763917 | 5.11E-06 | 7266 | Ruminococcus1 | 0.0427 | 0.002848619 | 20.7514803 |
| 5 | 15944587 | rs78572139 | A | G | 0.1250377 | 0.027941129 | 5.23E-06 | 11667 | Ruminococcus1 | 0.0706 | 0.001713523 | 20.02256021 |
| 1 | 12778598 | rs3000856 | A | T | -0.070999702 | 0.016276599 | 9.28E-06 | 16126 | Ruminococcus1 | 0.1342 | 0.001178547 | 19.02531563 |
| 15 | 55439182 | rs6493760 | T | C | 0.053526195 | 0.011588457 | 3.38E-06 | 16126 | Ruminococcus1 | 0.3966 | 0.001321237 | 21.33180582 |
| 8 | 144596903 | rs11783695 | T | G | -0.07341143 | 0.016139812 | 4.73E-06 | 16606 | Ruminococcus1 | 0.1511 | 0.001244299 | 20.68607696 |
| 10 | 65870347 | rs10995816 | G | C | -0.07620021 | 0.017468886 | 8.38E-06 | 16597 | Ruminococcus1 | 0.1123 | 0.00114513 | 19.02521541 |
| 2 | 181882879 | rs10167839 | G | A | 0.051950897 | 0.011630648 | 8.09E-06 | 16605 | Ruminococcus1 | 0.3439 | 0.001200101 | 19.94922088 |
| 1 | 241789757 | rs3819978 | T | C | -0.115039796 | 0.026013681 | 8.74E-06 | 12769 | Ruminococcus1 | 0.0586 | 0.001529224 | 19.55350282 |
| 2 | 119481372 | rs4849717 | A | T | 0.132763838 | 0.029981613 | 8.83E-06 | 10484 | Ruminococcus1 | 0.0527 | 0.001866857 | 19.60499626 |
| 20 | 13258628 | rs6105066 | C | T | -0.060705188 | 0.01342499 | 5.06E-06 | 16049 | Ruminococcus1 | 0.2495 | 0.001272397 | 20.44417423 |
| 3 | 106664363 | rs78120384 | G | A | -0.192815497 | 0.039204507 | 3.31E-07 | 6082 | Ruminococcus2 | 0.0726 | 0.003961336 | 24.18070986 |
| 2 | 238103439 | rs12986628 | T | C | 0.066593727 | 0.014016808 | 2.14E-06 | 14714 | Ruminococcus2 | 0.2127 | 0.001531694 | 22.56884683 |
| 3 | 173040924 | rs7635831 | A | G | 0.061834357 | 0.012903108 | 1.98E-06 | 14924 | Ruminococcus2 | 0.2932 | 0.001536448 | 22.96216338 |
| 13 | 62067781 | rs2997412 | G | A | -0.056843902 | 0.012232461 | 4.22E-06 | 15336 | Ruminococcus2 | 0.2803 | 0.001406102 | 21.59153065 |
| 2 | 182656416 | rs2368224 | G | T | 0.199647633 | 0.043845344 | 3.63E-06 | 3026 | Ruminococcus2 | 0.0477 | 0.006805293 | 20.72021391 |
| 7 | 16804578 | rs4400279 | G | A | 0.05461138 | 0.012007747 | 5.80E-06 | 15333 | Ruminococcus2 | 0.3638 | 0.001347196 | 20.68171672 |
| 9 | 137557890 | rs58681734 | G | A | 0.07238752 | 0.016078702 | 4.18E-06 | 15338 | Ruminococcus2 | 0.1511 | 0.001319724 | 20.26603525 |
| 7 | 151435722 | rs1819812 | T | G | 0.084235642 | 0.018489341 | 5.28E-06 | 14868 | Ruminococcus2 | 0.1163 | 0.001394089 | 20.75345819 |
| 1 | 108074445 | rs12406309 | C | A | -0.063192979 | 0.014227548 | 9.79E-06 | 15339 | Ruminococcus2 | 0.1909 | 0.001284465 | 19.72517629 |
| 18 | 862477 | rs2846589 | T | G | 0.052209321 | 0.011638161 | 7.59E-06 | 14924 | Ruminococcus2 | 0.4543 | 0.001346657 | 20.12190857 |
| 9 | 79919385 | rs55707116 | A | C | 0.08655215 | 0.01891409 | 8.01E-06 | 15336 | Ruminococcus2 | 0.1024 | 0.001363578 | 20.93766084 |
| 11 | 12148588 | rs75140805 | G | T | 0.083682495 | 0.017644509 | 3.95E-06 | 14249 | Ruminococcus2 | 0.1581 | 0.001576089 | 22.48998406 |
| 18 | 33456427 | rs4799823 | T | C | 0.083700126 | 0.018226258 | 5.40E-06 | 14650 | Ruminococcus2 | 0.1392 | 0.001437457 | 21.08617956 |
| 4 | 24655463 | rs61791565 | C | T | -0.052350302 | 0.011711882 | 6.79E-06 | 15319 | Ruminococcus2 | 0.3688 | 0.001302532 | 19.97690947 |
| 4 | 4268322 | rs7693984 | A | G | -0.102821803 | 0.023508663 | 9.42E-06 | 14154 | Ruminococcus2 | 0.0825 | 0.001349736 | 19.12727981 |
| 10 | 29201860 | rs2047242 | G | A | -0.06760301 | 0.013373773 | 2.46E-07 | 13369 | Ruminococcusgauvreauiigroup | 0.2972 | 0.001907638 | 25.54813323 |
| 16 | 2767894 | rs71386687 | G | T | 0.121036908 | 0.02385996 | 2.91E-07 | 11340 | Ruminococcusgauvreauiigroup | 0.0596 | 0.002264116 | 25.72880429 |
| 3 | 112372317 | rs9870933 | G | A | 0.062164279 | 0.012607163 | 8.49E-07 | 13382 | Ruminococcusgauvreauiigroup | 0.4085 | 0.001813584 | 24.30984417 |
| 12 | 61119266 | rs1391597 | T | C | 0.059028381 | 0.012487351 | 1.86E-06 | 13382 | Ruminococcusgauvreauiigroup | 0.3807 | 0.001667 | 22.3416986 |
| 5 | 166419552 | rs431418 | G | A | -0.094737043 | 0.02101571 | 5.54E-06 | 13289 | Ruminococcusgauvreauiigroup | 0.1193 | 0.001526847 | 20.31824295 |
| 2 | 191954852 | rs10931481 | A | G | 0.061009638 | 0.013048446 | 3.38E-06 | 13382 | Ruminococcusgauvreauiigroup | 0.3111 | 0.001630983 | 21.85820101 |
| 15 | 85563483 | rs289410 | A | G | -0.065492577 | 0.013910104 | 2.27E-06 | 13380 | Ruminococcusgauvreauiigroup | 0.2684 | 0.001654049 | 22.16452764 |
| 1 | 20552072 | rs2105937 | G | A | 0.058021543 | 0.012780587 | 5.10E-06 | 13364 | Ruminococcusgauvreauiigroup | 0.334 | 0.001539825 | 20.60686703 |
| 8 | 137090850 | rs2166943 | C | A | 0.056697379 | 0.012349673 | 5.28E-06 | 13377 | Ruminococcusgauvreauiigroup | 0.4384 | 0.001573159 | 21.0741527 |
| 7 | 153487396 | rs12539819 | T | C | 0.110653536 | 0.02406836 | 4.49E-06 | 11860 | Ruminococcusgauvreauiigroup | 0.0954 | 0.001779015 | 21.13315593 |
| 1 | 161880482 | rs12079579 | G | A | 0.095532379 | 0.021343619 | 5.04E-06 | 12986 | Ruminococcusgauvreauiigroup | 0.0726 | 0.001540353 | 20.03079368 |
| 4 | 18824860 | rs73802842 | A | C | 0.07368081 | 0.016966478 | 7.48E-06 | 13382 | Ruminococcusgauvreauiigroup | 0.1431 | 0.00140732 | 18.85647281 |
| 5 | 112627773 | rs13188803 | A | T | 0.070941873 | 0.015685519 | 7.28E-06 | 12834 | Ruminococcusgauvreauiigroup | 0.1879 | 0.001591306 | 20.45218918 |
| 5 | 18662686 | rs13163520 | A | G | -0.127377481 | 0.023388325 | 5.61E-08 | 6326 | Ruminococcusgnavusgroup | 0.1809 | 0.004666877 | 29.65170882 |
| 2 | 122096018 | rs934940 | C | A | -0.105044905 | 0.022958834 | 2.74E-06 | 6309 | Ruminococcusgnavusgroup | 0.1849 | 0.003307129 | 20.927272 |
| 3 | 66701616 | rs9872758 | C | T | 0.084923254 | 0.017664313 | 1.66E-06 | 6398 | Ruminococcusgnavusgroup | 0.4771 | 0.00359956 | 23.10595686 |
| 8 | 129224680 | rs2909242 | A | C | -0.090996476 | 0.018351012 | 7.41E-07 | 6401 | Ruminococcusgnavusgroup | 0.3857 | 0.003826628 | 24.58065121 |
| 9 | 135838591 | rs3124783 | G | A | -0.115989721 | 0.02490991 | 2.67E-06 | 6401 | Ruminococcusgnavusgroup | 0.1203 | 0.003375812 | 21.67499314 |
| 2 | 144700007 | rs62167033 | C | T | 0.185289033 | 0.039629529 | 3.50E-06 | 5194 | Ruminococcusgnavusgroup | 0.0706 | 0.004191173 | 21.85215861 |
| 2 | 36259341 | rs12989336 | A | G | -0.084686061 | 0.01879361 | 7.12E-06 | 6401 | Ruminococcusgnavusgroup | 0.3052 | 0.003162133 | 20.29867622 |
| 1 | 115168097 | rs12136548 | T | C | 0.090194566 | 0.019647574 | 3.10E-06 | 6401 | Ruminococcusgnavusgroup | 0.2913 | 0.003281463 | 21.06721605 |
| 3 | 146290310 | rs78399089 | C | T | 0.144444798 | 0.032661769 | 6.63E-06 | 5944 | Ruminococcusgnavusgroup | 0.0825 | 0.003279585 | 19.55141756 |
| 10 | 6512581 | rs11597105 | G | A | 0.114672834 | 0.025065269 | 6.95E-06 | 6328 | Ruminococcusgnavusgroup | 0.1123 | 0.003296672 | 20.92372753 |
| 16 | 433078 | rs11864644 | C | T | -0.139803729 | 0.031825335 | 5.01E-06 | 6241 | Ruminococcusgnavusgroup | 0.0706 | 0.003082454 | 19.29089424 |
| 4 | 190110710 | rs4388134 | T | C | -0.090499668 | 0.020354415 | 9.12E-06 | 6399 | Ruminococcusgnavusgroup | 0.2455 | 0.003079818 | 19.76245923 |
| 19 | 49218060 | rs35866622 | C | T | -0.061202384 | 0.010942125 | 2.21E-08 | 17240 | Ruminococcustorquesgroup | 0.4404 | 0.001811376 | 31.28116815 |
| 12 | 56494998 | rs773123 | A | T | 0.082400465 | 0.017386159 | 1.59E-06 | 17011 | Ruminococcustorquesgroup | 0.1123 | 0.001318709 | 22.45954423 |
| 10 | 4639762 | rs10904297 | G | A | -0.167804385 | 0.038981237 | 2.69E-06 | 3143 | Ruminococcustorquesgroup | 0.0298 | 0.005861354 | 18.51905963 |
| 17 | 14532347 | rs8080469 | A | G | 0.049072662 | 0.010703625 | 3.50E-06 | 17798 | Ruminococcustorquesgroup | 0.5189 | 0.001179597 | 21.01689891 |
| 8 | 76769843 | rs77034621 | G | T | -0.151552842 | 0.033587775 | 6.07E-06 | 8567 | Ruminococcustorquesgroup | 0.0527 | 0.002370861 | 20.35468082 |
| 7 | 54781314 | rs73130967 | T | A | 0.077037483 | 0.016826423 | 3.71E-06 | 17011 | Ruminococcustorquesgroup | 0.1123 | 0.00123071 | 20.95894866 |
| 15 | 25326425 | rs1972694 | A | T | -0.061409435 | 0.013732055 | 8.93E-06 | 17353 | Ruminococcustorquesgroup | 0.1839 | 0.00115113 | 19.99627319 |
| 14 | 26130404 | rs12434631 | G | A | 0.074696994 | 0.015340053 | 2.77E-06 | 17353 | Ruminococcustorquesgroup | 0.1402 | 0.001364536 | 23.70841125 |
| 11 | 132663147 | rs4073731 | C | T | 0.065194801 | 0.014222118 | 4.05E-06 | 17793 | Ruminococcustorquesgroup | 0.1759 | 0.001179602 | 21.01108761 |
| 5 | 56952803 | rs13154778 | A | T | 0.056335821 | 0.012952644 | 7.16E-06 | 17795 | Ruminococcustorquesgroup | 0.1859 | 0.001061923 | 18.91488065 |
| 6 | 67262102 | rs1475330 | C | T | 0.052349242 | 0.011820049 | 8.13E-06 | 17353 | Ruminococcustorquesgroup | 0.3012 | 0.00112906 | 19.61246373 |
| 9 | 27225258 | rs10967781 | A | C | 0.050799379 | 0.011332229 | 8.37E-06 | 17791 | Ruminococcustorquesgroup | 0.3181 | 0.001128224 | 20.09265256 |
| 22 | 21358653 | rs8141465 | G | A | 0.048077756 | 0.010744211 | 9.65E-06 | 17791 | Ruminococcustorquesgroup | 0.5427 | 0.001124218 | 20.02121966 |
| 2 | 174308394 | rs13417181 | C | T | 0.166509314 | 0.033752138 | 7.62E-07 | 3047 | Sellimonas | 0.2157 | 0.007924046 | 24.32144302 |
| 2 | 213214174 | rs2371572 | C | A | 0.127348939 | 0.025086238 | 4.46E-07 | 3154 | Sellimonas | 0.5487 | 0.008104456 | 25.75396561 |
| 3 | 42008186 | rs113379006 | C | T | -0.162758788 | 0.035702495 | 7.21E-06 | 3154 | Sellimonas | 0.168 | 0.006546028 | 20.76903338 |
| 11 | 79332972 | rs2187447 | C | A | 0.243457117 | 0.052769543 | 3.98E-06 | 3028 | Sellimonas | 0.0606 | 0.006980399 | 21.27116767 |
| 15 | 52105282 | rs2016057 | C | A | -0.125877526 | 0.02561532 | 1.03E-06 | 3154 | Sellimonas | 0.3907 | 0.007598408 | 24.13355765 |
| 1 | 247579710 | rs72553859 | C | G | -0.150458067 | 0.033262641 | 5.38E-06 | 3154 | Sellimonas | 0.175 | 0.006445366 | 20.44758447 |
| 7 | 111501524 | rs56203279 | C | T | -0.124045002 | 0.026911497 | 3.72E-06 | 3154 | Sellimonas | 0.33 | 0.006691222 | 21.23280434 |
| 6 | 93730393 | rs553697 | C | T | -0.153767275 | 0.033909892 | 6.13E-06 | 3047 | Sellimonas | 0.162 | 0.006703191 | 20.54896106 |
| 12 | 100709313 | rs7968030 | T | A | -0.127110283 | 0.028061737 | 5.56E-06 | 3095 | Sellimonas | 0.2833 | 0.006585701 | 20.50461127 |
| 2 | 180278564 | rs4600608 | G | A | -0.137151754 | 0.03017017 | 4.95E-06 | 3095 | Sellimonas | 0.2386 | 0.006632792 | 20.65220786 |
| 7 | 106250164 | rs41816 | G | A | 0.132201227 | 0.029108726 | 8.39E-06 | 3154 | Sellimonas | 0.2515 | 0.006497286 | 20.61337522 |
| 9 | 34332382 | rs11787826 | A | C | 0.081327043 | 0.017115415 | 2.63E-06 | 6923 | Senegalimassilia | 0.4284 | 0.003250773 | 22.57197644 |
| 17 | 74949284 | rs72887800 | A | T | -0.082246156 | 0.017572066 | 2.42E-06 | 6923 | Senegalimassilia | 0.3777 | 0.003154417 | 21.90080444 |
| 17 | 48379615 | rs7225245 | A | G | 0.079173009 | 0.017042101 | 4.18E-06 | 6923 | Senegalimassilia | 0.4583 | 0.003107863 | 21.57657776 |
| 13 | 19489214 | rs57512504 | A | T | 0.081949442 | 0.017224097 | 2.03E-06 | 6923 | Senegalimassilia | 0.4612 | 0.00325917 | 22.63047069 |
| 5 | 127861692 | rs10036909 | T | C | 0.185519381 | 0.040088092 | 8.05E-06 | 4996 | Senegalimassilia | 0.0696 | 0.004268425 | 21.40789267 |
| 2 | 220831701 | rs13383270 | C | G | 0.077467918 | 0.017084039 | 6.04E-06 | 6923 | Senegalimassilia | 0.4195 | 0.002961286 | 20.55593269 |
| 2 | 206654123 | rs1990708 | C | A | -0.109619487 | 0.024778524 | 8.91E-06 | 6923 | Senegalimassilia | 0.1262 | 0.00281906 | 19.56587067 |
| 14 | 34431996 | rs2017373 | T | C | 0.078225822 | 0.017684094 | 9.50E-06 | 6923 | Senegalimassilia | 0.3738 | 0.002818477 | 19.56181518 |
| 17 | 74267010 | rs8901 | T | C | 0.093459378 | 0.018681296 | 6.07E-07 | 6063 | Slackia | 0.3708 | 0.004111072 | 25.02006717 |
| 7 | 13523683 | rs4492265 | G | A | -0.0905757 | 0.019165844 | 2.41E-06 | 6063 | Slackia | 0.3101 | 0.003670144 | 22.32668304 |
| 8 | 96946291 | rs16894137 | T | C | -0.12279207 | 0.026304664 | 2.71E-06 | 6063 | Slackia | 0.1431 | 0.003581207 | 21.78370955 |
| 7 | 78282725 | rs112764253 | A | T | 0.194713531 | 0.041159784 | 3.40E-06 | 5634 | Slackia | 0.0408 | 0.003956467 | 22.37133419 |
| 15 | 34041896 | rs12440440 | G | A | 0.090193357 | 0.019058248 | 2.63E-06 | 6063 | Slackia | 0.3091 | 0.003680394 | 22.38926759 |
| 7 | 99451715 | rs35156985 | C | T | -0.155708877 | 0.034808468 | 8.06E-06 | 5965 | Slackia | 0.0636 | 0.003343428 | 20.00374443 |
| 19 | 4555786 | rs10409783 | G | A | 0.095082845 | 0.021123946 | 7.70E-06 | 5941 | Slackia | 0.2833 | 0.003398726 | 20.25387078 |
| 16 | 7460475 | rs13339230 | G | C | 0.147054846 | 0.033063509 | 7.42E-06 | 6063 | Slackia | 0.0885 | 0.00325206 | 19.775047 |
| 4 | 57167507 | rs58767323 | C | G | -0.102833438 | 0.022707713 | 4.60E-06 | 6063 | Slackia | 0.1899 | 0.003371071 | 20.50116997 |
| 12 | 100584014 | rs11110281 | C | T | -0.137518884 | 0.022739814 | 2.58E-09 | 15120 | Streptococcus | 0.0676 | 0.002412959 | 36.56734357 |
| 17 | 61298020 | rs4968759 | G | A | -0.051510944 | 0.011206836 | 3.78E-06 | 16384 | Streptococcus | 0.4264 | 0.001287815 | 21.12418676 |
| 7 | 46774896 | rs11764382 | G | A | -0.06953447 | 0.014367132 | 1.29E-06 | 16387 | Streptococcus | 0.1909 | 0.001427382 | 23.42109251 |
| 15 | 41663393 | rs72739637 | G | A | 0.095994182 | 0.019321261 | 1.03E-06 | 14920 | Streptococcus | 0.1064 | 0.001651704 | 24.68088187 |
| 11 | 131649446 | rs1918540 | A | G | 0.059639038 | 0.012814811 | 2.44E-06 | 16373 | Streptococcus | 0.2525 | 0.001321096 | 21.65627772 |
| 8 | 10199548 | rs17708276 | G | A | -0.079395519 | 0.017062753 | 3.04E-06 | 16278 | Streptococcus | 0.1282 | 0.001328359 | 21.64912918 |
| 3 | 94103591 | rs11720390 | A | G | 0.107023904 | 0.022812145 | 3.59E-06 | 15220 | Streptococcus | 0.0746 | 0.001444066 | 22.00757791 |
| 5 | 169689604 | rs395407 | C | G | -0.079278061 | 0.01736973 | 4.37E-06 | 15864 | Streptococcus | 0.1421 | 0.001311406 | 20.82884432 |
| 9 | 93556174 | rs10448310 | G | A | -0.051793473 | 0.011132356 | 3.31E-06 | 16384 | Streptococcus | 0.4304 | 0.001319419 | 21.64327094 |
| 10 | 28588269 | rs7916711 | G | A | 0.102890941 | 0.021736227 | 2.72E-06 | 15294 | Streptococcus | 0.0646 | 0.001462949 | 22.40418491 |
| 10 | 8102404 | rs71481756 | G | T | 0.09310478 | 0.020794918 | 6.51E-06 | 14526 | Streptococcus | 0.0994 | 0.001378112 | 20.0433229 |
| 14 | 97526750 | rs2370083 | T | G | -0.081683584 | 0.018585095 | 9.75E-06 | 16277 | Streptococcus | 0.0885 | 0.001185361 | 19.31464041 |
| 4 | 53657577 | rs10028567 | T | C | -0.092116652 | 0.019188114 | 7.30E-06 | 16311 | Streptococcus | 0.0815 | 0.00141097 | 23.04403021 |
| 3 | 132058723 | rs6806351 | C | T | -0.063382863 | 0.013664741 | 4.94E-06 | 15973 | Streptococcus | 0.2087 | 0.001345147 | 21.51228287 |
| 4 | 37452858 | rs57646748 | A | G | -0.090769573 | 0.020034438 | 5.48E-06 | 15939 | Streptococcus | 0.0676 | 0.001286193 | 20.52446031 |
| 17 | 80576624 | rs9903102 | A | C | -0.070948345 | 0.015527498 | 4.18E-06 | 16384 | Streptococcus | 0.164 | 0.001272649 | 20.87510049 |
| 16 | 73391982 | rs6563952 | C | G | 0.082734414 | 0.018003533 | 5.82E-06 | 16387 | Streptococcus | 0.0924 | 0.001287058 | 21.11562414 |
| 3 | 62327558 | rs12638227 | C | G | -0.055728761 | 0.010855202 | 2.48E-07 | 17165 | Subdoligranulum | 0.4652 | 0.001533108 | 26.35313939 |
| 5 | 141857415 | rs10065321 | C | T | -0.051282785 | 0.010810523 | 2.10E-06 | 17589 | Subdoligranulum | 0.3678 | 0.001277772 | 22.50092728 |
| 2 | 218215847 | rs4347804 | G | A | 0.166063165 | 0.035748326 | 2.18E-06 | 4312 | Subdoligranulum | 0.0417 | 0.004979531 | 21.56918459 |
| 5 | 4671383 | rs6555306 | C | T | -0.074077668 | 0.015546287 | 2.81E-06 | 17591 | Subdoligranulum | 0.1223 | 0.001289052 | 22.70240589 |
| 4 | 48990885 | rs3761728 | G | T | -0.054345782 | 0.011886608 | 3.87E-06 | 17570 | Subdoligranulum | 0.2425 | 0.001188304 | 20.90096699 |
| 10 | 125472937 | rs2114677 | T | C | -0.104176347 | 0.023082878 | 2.72E-06 | 15566 | Subdoligranulum | 0.0557 | 0.001306813 | 20.36585023 |
| 19 | 29644694 | rs75158211 | C | T | -0.07234304 | 0.015933044 | 7.52E-06 | 17165 | Subdoligranulum | 0.1113 | 0.001199585 | 20.61319998 |
| 6 | 153694029 | rs2171249 | T | C | 0.10674366 | 0.023321252 | 4.51E-06 | 14679 | Subdoligranulum | 0.0736 | 0.001425163 | 20.94697401 |
| 14 | 86933898 | rs35940633 | A | G | -0.051100832 | 0.011001029 | 4.22E-06 | 17582 | Subdoligranulum | 0.3628 | 0.001225712 | 21.57446081 |
| 5 | 117799448 | rs76528319 | T | G | -0.143278588 | 0.031066225 | 7.41E-06 | 8468 | Subdoligranulum | 0.0398 | 0.00250562 | 21.26586361 |
| 2 | 232408743 | rs1667315 | A | G | 0.0485017 | 0.010745207 | 6.72E-06 | 17586 | Subdoligranulum | 0.4374 | 0.001157216 | 20.37205927 |
| 3 | 183716408 | rs76664262 | A | T | 0.083407827 | 0.018535669 | 4.87E-06 | 16712 | Subdoligranulum | 0.0934 | 0.00121016 | 20.24627315 |
| 2 | 200288718 | rs10497836 | T | C | -0.052406498 | 0.011869733 | 8.38E-06 | 17579 | Subdoligranulum | 0.2803 | 0.001107676 | 19.49121659 |
| 16 | 13684415 | rs16962433 | T | A | 0.085599862 | 0.018921315 | 7.65E-06 | 15857 | Subdoligranulum | 0.1083 | 0.001289028 | 20.46391363 |
| 5 | 58499183 | rs13173038 | G | A | -0.071807564 | 0.015162698 | 2.73E-06 | 13245 | Sutterella | 0.2207 | 0.001690444 | 22.42445133 |
| 13 | 58689340 | rs2321387 | A | G | -0.059287622 | 0.012450922 | 1.87E-06 | 13252 | Sutterella | 0.5298 | 0.001708052 | 22.67041526 |
| 16 | 85038065 | rs7499539 | G | A | 0.061747829 | 0.013099845 | 2.36E-06 | 13254 | Sutterella | 0.3231 | 0.001673542 | 22.21495915 |
| 7 | 150126751 | rs62501473 | A | G | 0.069423259 | 0.014941649 | 5.52E-06 | 12762 | Sutterella | 0.2286 | 0.00168873 | 21.58464247 |
| 1 | 63278549 | rs143438747 | C | T | -0.145792693 | 0.030686706 | 3.28E-06 | 9368 | Sutterella | 0.0547 | 0.002403693 | 22.56722917 |
| 6 | 18583878 | rs9350083 | G | T | -0.059306886 | 0.013392944 | 8.23E-06 | 13254 | Sutterella | 0.2992 | 0.001477301 | 19.60616261 |
| 3 | 70588939 | rs7638039 | C | T | 0.064562374 | 0.014388651 | 8.66E-06 | 13236 | Sutterella | 0.2326 | 0.001518805 | 20.1304372 |
| 10 | 102520687 | rs11591622 | G | T | -0.0688382 | 0.015137492 | 6.50E-06 | 12604 | Sutterella | 0.2157 | 0.001638064 | 20.67674778 |
| 7 | 111285025 | rs2613606 | T | C | -0.055679377 | 0.012411747 | 7.20E-06 | 13249 | Sutterella | 0.3936 | 0.001516633 | 20.12136057 |
| 6 | 82386821 | rs1145877 | G | A | -0.073548024 | 0.016241333 | 7.20E-06 | 13251 | Sutterella | 0.159 | 0.001545178 | 20.50374215 |
| 11 | 111695709 | rs607327 | T | C | 0.057833353 | 0.012905264 | 6.63E-06 | 13251 | Sutterella | 0.3767 | 0.001513271 | 20.07971 |
| 6 | 147936781 | rs2050185 | A | G | 0.057513842 | 0.012876636 | 7.97E-06 | 13243 | Sutterella | 0.3777 | 0.00150418 | 19.94684971 |
| 11 | 8938819 | rs1883097 | T | C | 0.226398815 | 0.045464179 | 4.16E-07 | 4417 | Terrisporobacter | 0.0557 | 0.005582784 | 24.78636842 |
| 3 | 144148905 | rs2569953 | C | A | -0.077559072 | 0.01746408 | 8.95E-06 | 6637 | Terrisporobacter | 0.4195 | 0.002962875 | 19.71709248 |
| 16 | 15920394 | rs7184125 | C | T | 0.091256006 | 0.020549332 | 8.48E-06 | 6232 | Terrisporobacter | 0.2326 | 0.003154481 | 19.71460607 |
| 19 | 17026940 | rs2872237 | A | C | -0.08145049 | 0.017594312 | 3.97E-06 | 6632 | Terrisporobacter | 0.4344 | 0.003221048 | 21.42455726 |
| 11 | 20621043 | rs58405430 | T | G | 0.134617158 | 0.030117736 | 7.94E-06 | 6176 | Terrisporobacter | 0.1153 | 0.003224381 | 19.97172299 |
| 9 | 117245252 | rs7034891 | C | G | -0.079920945 | 0.01737936 | 4.54E-06 | 6629 | Terrisporobacter | 0.4841 | 0.003179966 | 21.14085902 |
| 2 | 63366962 | rs149744580 | G | A | 0.169882696 | 0.031547837 | 7.01E-08 | 8459 | Turicibacter | 0.0527 | 0.003416283 | 28.99054798 |
| 5 | 95717619 | rs4869133 | A | G | 0.131186095 | 0.027196968 | 2.55E-06 | 8256 | Turicibacter | 0.0885 | 0.002810236 | 23.2610547 |
| 17 | 43117476 | rs12603364 | C | T | 0.110860918 | 0.022559841 | 8.67E-07 | 8923 | Turicibacter | 0.1074 | 0.002698984 | 24.14279336 |
| 12 | 12242455 | rs11054680 | C | T | -0.104751216 | 0.02269975 | 2.31E-06 | 8928 | Turicibacter | 0.0964 | 0.002379508 | 21.29014885 |
| 7 | 130972179 | rs55756211 | C | T | -0.115115214 | 0.024070764 | 2.81E-06 | 8941 | Turicibacter | 0.1163 | 0.002551468 | 22.86591204 |
| 11 | 1681862 | rs61265175 | C | G | -0.085859068 | 0.018577761 | 4.14E-06 | 8941 | Turicibacter | 0.2326 | 0.002383215 | 21.35445272 |
| 16 | 49826299 | rs7199484 | A | G | -0.07314282 | 0.016017166 | 5.77E-06 | 8948 | Turicibacter | 0.332 | 0.002325065 | 20.84850677 |
| 16 | 10181248 | rs11649454 | C | G | 0.095089088 | 0.020343331 | 3.27E-06 | 8943 | Turicibacter | 0.1392 | 0.002437105 | 21.84339263 |
| 21 | 36929643 | rs2834977 | C | T | -0.095999549 | 0.020826107 | 3.96E-06 | 8946 | Turicibacter | 0.173 | 0.002369534 | 21.24345182 |
| 8 | 26027842 | rs2952020 | A | G | -0.075901896 | 0.016576375 | 5.63E-06 | 8949 | Turicibacter | 0.2227 | 0.002337412 | 20.96182379 |
| 19 | 11864932 | rs11666533 | T | C | -0.111689111 | 0.024843618 | 7.37E-06 | 8256 | Turicibacter | 0.0795 | 0.002442083 | 20.20629898 |
| 15 | 92605098 | rs4247078 | G | C | 0.071037672 | 0.015522123 | 5.46E-06 | 8948 | Turicibacter | 0.338 | 0.002335253 | 20.94006942 |
| 5 | 170048048 | rs2221441 | C | G | 0.071036413 | 0.015343033 | 3.46E-06 | 8720 | Turicibacter | 0.4056 | 0.002452205 | 21.43087834 |
| 6 | 126111881 | rs3734633 | A | G | -0.120956846 | 0.026830036 | 5.32E-06 | 8626 | Turicibacter | 0.0974 | 0.002350644 | 20.31971791 |
| 19 | 1030320 | rs67476743 | G | T | 0.132163631 | 0.02220797 | 3.74E-09 | 4948 | Tyzzerella3 | 0.2853 | 0.007106881 | 35.40223127 |
| 5 | 16388259 | rs17706273 | C | T | -0.140368365 | 0.027470793 | 5.88E-07 | 5395 | Tyzzerella3 | 0.1521 | 0.004816238 | 26.09967325 |
| 2 | 57810406 | rs7561370 | C | T | 0.131341092 | 0.028629245 | 1.52E-06 | 5396 | Tyzzerella3 | 0.1223 | 0.003885249 | 21.0387754 |
| 17 | 3738781 | rs55799124 | G | A | -0.114350138 | 0.023860417 | 1.34E-06 | 5362 | Tyzzerella3 | 0.2068 | 0.004265149 | 22.95912357 |
| 7 | 82547827 | rs17809157 | T | A | -0.163819473 | 0.033638641 | 1.54E-06 | 4621 | Tyzzerella3 | 0.0994 | 0.005106166 | 23.70643014 |
| 9 | 33113322 | rs7019909 | C | T | 0.144155503 | 0.030162645 | 1.76E-06 | 5396 | Tyzzerella3 | 0.1014 | 0.00421519 | 22.83298103 |
| 13 | 35023691 | rs75091807 | T | G | -0.184966239 | 0.038302914 | 1.71E-06 | 4621 | Tyzzerella3 | 0.0905 | 0.005021104 | 23.30951774 |
| 14 | 89595945 | rs4904512 | C | T | -0.117150935 | 0.025030561 | 3.09E-06 | 5396 | Tyzzerella3 | 0.1988 | 0.004043141 | 21.89723849 |
| 13 | 81593016 | rs7333521 | C | T | -0.20719145 | 0.045312058 | 4.88E-06 | 4335 | Tyzzerella3 | 0.0427 | 0.004799957 | 20.89852652 |
| 10 | 46098286 | rs112102233 | G | A | -0.216348445 | 0.04775752 | 6.18E-06 | 3857 | Tyzzerella3 | 0.0666 | 0.005292608 | 20.51156327 |
| 6 | 102680257 | rs1232220 | T | G | -0.143869315 | 0.031828041 | 7.91E-06 | 5000 | Tyzzerella3 | 0.1262 | 0.004069822 | 20.42409341 |
| 12 | 77390329 | rs191093 | A | G | 0.15900811 | 0.035330566 | 6.76E-06 | 4621 | Tyzzerella3 | 0.0795 | 0.004364173 | 20.24647189 |
| 11 | 87588698 | rs10898797 | T | C | 0.122380253 | 0.027468252 | 8.85E-06 | 5387 | Tyzzerella3 | 0.1571 | 0.00367127 | 19.84263672 |
| 6 | 5755626 | rs6920448 | T | C | -0.141080632 | 0.030544578 | 4.15E-06 | 5010 | Tyzzerella3 | 0.1153 | 0.004240172 | 21.32520142 |
| 11 | 44302154 | rs2013594 | C | T | -0.072069819 | 0.01551532 | 3.42E-06 | 9283 | Veillonella | 0.3579 | 0.002318936 | 21.57206895 |
| 8 | 13253770 | rs12679709 | G | C | -0.079349857 | 0.016481776 | 1.78E-06 | 9290 | Veillonella | 0.3211 | 0.002488779 | 23.17345432 |
| 22 | 45604800 | rs742016 | G | A | -0.068857084 | 0.01497691 | 4.66E-06 | 9279 | Veillonella | 0.4235 | 0.002272812 | 21.13290521 |
| 21 | 30010673 | rs1882878 | G | A | -0.07689658 | 0.016390829 | 2.98E-06 | 9282 | Veillonella | 0.3062 | 0.002365604 | 22.00486138 |
| 3 | 125544032 | rs7645873 | T | A | 0.076138533 | 0.016422757 | 3.12E-06 | 9286 | Veillonella | 0.2833 | 0.00230932 | 21.48935347 |
| 11 | 2956783 | rs55807413 | G | A | 0.107329355 | 0.023763343 | 5.51E-06 | 8610 | Veillonella | 0.0974 | 0.002363693 | 20.39487642 |
| 5 | 119914285 | rs62376424 | T | C | -0.076221631 | 0.016349923 | 3.65E-06 | 8999 | Veillonella | 0.334 | 0.002409257 | 21.7284386 |
| 14 | 106939916 | rs7359080 | A | C | -0.135390734 | 0.030351973 | 7.40E-06 | 6189 | Veillonella | 0.1272 | 0.003204716 | 19.8913229 |
| 9 | 89306305 | rs11141494 | A | G | -0.078342138 | 0.017439254 | 9.75E-06 | 9291 | Veillonella | 0.1948 | 0.002167356 | 20.17630006 |
| 1 | 178945043 | rs6656807 | G | A | 0.070306243 | 0.015395397 | 5.50E-06 | 8999 | Veillonella | 0.4245 | 0.002312099 | 20.85016462 |
| 12 | 129984555 | rs11614532 | C | G | 0.074453782 | 0.016521422 | 7.13E-06 | 8411 | Veillonella | 0.3052 | 0.002408705 | 20.3037095 |
| 11 | 9312796 | rs56349194 | G | A | -0.158501176 | 0.031523075 | 6.26E-07 | 3266 | Victivallis | 0.16 | 0.007681442 | 25.26630822 |
| 9 | 136309841 | rs592514 | A | T | -0.181448867 | 0.039167751 | 2.60E-06 | 3241 | Victivallis | 0.1282 | 0.006578183 | 21.44782086 |
| 14 | 96071125 | rs911666 | C | T | -0.118575703 | 0.026314576 | 7.65E-06 | 3267 | Victivallis | 0.3052 | 0.006176731 | 20.29236695 |
| 12 | 102514184 | rs4764863 | A | G | 0.121560633 | 0.024605824 | 8.22E-07 | 3266 | Victivallis | 0.5 | 0.00741755 | 24.39181144 |
| 4 | 9608445 | rs12512543 | C | A | -0.1780361 | 0.037434441 | 2.54E-06 | 3239 | Victivallis | 0.1213 | 0.0069349 | 22.60503595 |
| 7 | 106378229 | rs342302 | G | A | -0.152754045 | 0.035157217 | 8.16E-06 | 3265 | Victivallis | 0.159 | 0.005748697 | 18.86645659 |
| 6 | 131630319 | rs4895919 | C | T | -0.116917513 | 0.024751549 | 2.75E-06 | 3254 | Victivallis | 0.4821 | 0.006810343 | 22.29910037 |
| 2 | 38049069 | rs11899949 | A | G | 0.130567821 | 0.027623889 | 2.77E-06 | 3258 | Victivallis | 0.2773 | 0.006810573 | 22.32728885 |
| 3 | 57723877 | rs6445926 | C | G | 0.117030674 | 0.024976353 | 2.96E-06 | 3267 | Victivallis | 0.3966 | 0.006675493 | 21.94195965 |
| 13 | 72876881 | rs173120 | C | T | 0.133809734 | 0.029012714 | 7.65E-06 | 3263 | Victivallis | 0.2455 | 0.006476788 | 21.25849108 |
| 5 | 180579462 | rs2546432 | C | T | -0.110803155 | 0.024965483 | 9.93E-06 | 3266 | Victivallis | 0.4662 | 0.005995103 | 19.68603568 |
| 21 | 40405606 | rs1882775 | G | A | -0.138270295 | 0.031272974 | 8.73E-06 | 3258 | Victivallis | 0.2237 | 0.005964448 | 19.5367683 |
