# Supplementary material for: Intestinal microbiota and tuberculosis: Insights from Mendelian randomization
Source: Medicine (Baltimore). 2024 Jul 5;103(27):e38762. doi: 10.1097/MD.0000000000038762 (PMC11250452; doi:10.1097/MD.0000000000038762)
Supplement: Supplementary file 2 [file medi-103-e38762-s002.docx]

| Exposure | outcome | method | Q | Q df | Q pval |
| --- | --- | --- | --- | --- | --- |
| LachnospiraceaeUCG010 | RTB1 | MR Egger | 8.739 | 8.000 | 0.365 |
| LachnospiraceaeUCG010 | RTB1 | Inverse variance weighted | 8.748 | 9.000 | 0.461 |
| LachnospiraceaeUCG010 | RTB2 | MR Egger | 6.584 | 8.000 | 0.582 |
| LachnospiraceaeUCG010 | RTB2 | Inverse variance weighted | 6.604 | 9.000 | 0.678 |

**Table S2**  The heterogeneity analysis of the relationship between Lachnospiraceae UCG010and RTB1,RTB2.
