# Supplementary material for: Intestinal microbiota and tuberculosis: Insights from Mendelian randomization
Source: Medicine (Baltimore). 2024 Jul 5;103(27):e38762. doi: 10.1097/MD.0000000000038762 (PMC11250452; doi:10.1097/MD.0000000000038762)
Supplement: Supplementary file 3 [file medi-103-e38762-s003.docx]

| Exposure | Outcome | MR PRESSO | | MR-egger intercept | |
| --- | --- | --- | --- | --- | --- |
|  |  | RSSobs | pval | Intercept | pval |
| LachnospiraceaeUCG010 | RTB1 | 10.655 | 0.511 | 0.004 | 0.927 |
| LachnospiraceaeUCG010 | RTB2 | 7.927 | 0.742 | -0.011 | 0.890 |

**Table S3** The pleiotropy analysis of the relationship between Lachnospiraceae UCG010 and RTB1,RTB2.
